# Functional screening reveals genetic dependencies and diverging cell cycle control in atypical teratoid rhabdoid tumors

**DOI:** 10.1186/s13059-024-03438-w

**Published:** 2024-12-02

**Authors:** Daniel J. Merk, Foteini Tsiami, Sophie Hirsch, Bianca Walter, Lara A. Haeusser, Jens D. Maile, Aaron Stahl, Mohamed A. Jarboui, Anna Lechado-Terradas, Franziska Klose, Sepideh Babaei, Jakob Admard, Nicolas Casadei, Cristiana Roggia, Michael Spohn, Jens Schittenhelm, Stephan Singer, Ulrich Schüller, Federica Piccioni, Nicole S. Persky, Manfred Claassen, Marcos Tatagiba, Philipp J. Kahle, David E. Root, Markus Templin, Ghazaleh Tabatabai

**Affiliations:** 1 Department of Neurology and Interdisciplinary Neuro-Oncology, Hertie Institute for Clinical Brain Research, University Hospital Tübingen, Eberhard Karls University Tübingen, Tübingen, 72076 Germany; 2 NMI Natural and Medical Sciences Institute at the University of Tübingen, Reutlingen, 72770 Germany; 3 Institute for Ophtalmic Research, Department for Ophtalmology, University Hospital Tübingen, Eberhard Karls University Tübingen, Tübingen, 72076, Germany; 4 Core Facility for Medical Bioanalytics, Institute for Ophtalmic Research, University Hospital Tübingen, Eberhard Karls University Tübingen, Tübingen, 72076 Germany; 5 Laboratory of Functional Neurogenetics, Department of Neurodegeneration, Hertie Institute for Clinical Brain Research and German Center for Neurodegenerative Diseases, Tübingen, 72076 Germany; 6 Interfaculty Institute of Biochemistry, University of Tübingen, Tübingen, 72076 Germany; 7 Internal Medicine I, University Hospital Tübingen, Eberhard Karls University Tübingen, Tübingen, 72076 Germany; 8 Institute of Medical Genetics and Applied Genomics, University Hospital Tübingen, Eberhard Karls University Tübingen, Tübingen, 72076 Germany; 9 NGS Competence Center Tübingen, Eberhard Karls University Tübingen, Tübingen, 72076 Germany; 10 Research Institute Children’s Cancer Center, Hamburg, 20251 Germany; 11 Bioinformatics Core Facility, University Medical Center Hamburg-Eppendorf, Hamburg, 20251 Germany; 12 Institute of Neuropathology, Department of Pathology and Neuropathology, University Hospital Tübingen, Eberhard Karls University Tübingen, Tübingen, 72076 Germany; 13 Institute of General and Molecular Pathology, Department of Pathology and Neuropathology, University Hospital Tübingen, Eberhard Karls University Tübingen, Tübingen, 72076 Germany; 14 Department of Pediatric Hematology and Oncology, University Hospital Hamburg-Eppendorf, Hamburg, 20246 Germany; 15 Institute of Neuropathology, University Hospital Hamburg-Eppendorf, Hamburg, 20246 Germany; 16 Genetic Perturbation Platform, Broad Institute of MIT and Harvard, MA Cambridge, 02142 USA; 17 Merck Research Laboratories, MA Cambridge, 02141 USA; 18 Department of Computer Science, Eberhard Karls University Tübingen, Tübingen, 72076 Germany; 19 Department of Neurosurgery, University Hospital Tübingen, Eberhard Karls University Tübingen, Tübingen, 72076 Germany; 20 Cluster of Excellence iFIT (EXC 2180) “Image Guided and Functionally Instructed Tumor Therapies”, Eberhard Karls University Tübingen, Tübingen, 72076 Germany; 21 German Consortium for Translational Cancer Research (DKTK), Partner Site Tübingen, German Cancer Research Center (DKFZ), Heidelberg, 69120 Germany; 22 Cluster of Excellence (EXC 2064) “Machine learning”, Eberhard Karls University Tübingen, Tübingen, 72076 Germany; 23 Center for Neuro-Oncology, Comprehensive Cancer Center Tübingen Stuttgart, University Hospital Tübingen, Eberhard Karls University Tübingen, Tübingen, 72076 Germany

**Keywords:** Functional screening, CRISPR-Cas9, Genetic dependencies, Rhabdoid tumors, CDK4/6 inhibitors, AMBRA1, Tumor suppressor

## Abstract

**Background:**

Atypical teratoid rhabdoid tumors (ATRT) are incurable high-grade pediatric brain tumors. Despite intensive research efforts, the prognosis for ATRT patients under currently established treatment protocols is poor. While novel therapeutic strategies are urgently needed, the generation of molecular-driven treatment concepts is a challenge mainly due to the absence of actionable genetic alterations.

**Results:**

We here use a functional genomics approach to identify genetic dependencies in ATRT, validate selected hits using a functionally instructed small molecule drug library, and observe preferential activity in ATRT cells without subgroup-specific selectivity. CDK4/6 inhibitors are among the most potent drugs and display anti-tumor efficacy due to mutual exclusive dependency on *CDK4* or *CDK6*. Chemogenetic interactor screens reveal a broad spectrum of G1 phase cell cycle regulators that differentially enable cell cycle progression and modulate response to CDK4/6 inhibition in ATRT cells. In this regard, we find that the ubiquitin ligase substrate receptor AMBRA1 acts as a context-specific inhibitor of cell cycle progression by regulating key components of mitosis including aurora kinases.

**Conclusions:**

Our data provide a comprehensive resource of genetic and chemical dependencies in ATRTs, which will inform further preclinical evaluation of novel targeted therapies for this tumor entity. Furthermore, this study reveals a unique mechanism of cell cycle inhibition as the basis for tumor suppressive functions of AMBRA1.

**Supplementary Information:**

The online version contains supplementary material available at 10.1186/s13059-024-03438-w.

## Background

ATRT are highly malignant embryonal brain tumors that account for up to 50% of all central nervous system neoplasms in the first year of life [1–3]. ATRT preferentially occur in infants and young children, and isolated cases have been described in adults [4]. Loss-of-function alterations of *SMARCB1*, and rarely *SMARCA4*, serve as diagnostic molecular markers. The presence of alterations in these two components of the SWI/SNF chromatin remodelling complex suggests a major role for epigenetic dysregulation during initiation and progression of ATRT [5–8]. From a clinical perspective, however, the loss of a tumor suppressor gene (i.e., *SMARCB1*) cannot be directly exploited as a therapeutic drug target. Conventional next-generation sequencing studies have not detected any recurrent genetic alterations in druggable oncogenes [9, 10]. In spite of their consistent homogeneity on the genetic level, ATRT display a remarkable epigenetic and transcriptional heterogeneity [9, 11]. In fact, ATRT are currently segregated into three molecular subgroups primarily based on DNA methylation patterns: ATRT-SHH (formerly group 1), ATRT-TYR (group 2A), and ATRT-MYC (group 2B) [12]. Furthermore, additional sub-categorization within subgroups [13] and additional grouping of *SMARCA4*-mutated cases [14] has been suggested recently. While these distinct molecular profiles correlate with distinct clinical features (e.g., patient age, tumor location), they currently do not serve as predictive signatures for molecular-driven therapeutic strategies. Thus, novel approaches are needed to reveal potentially druggable vulnerabilities in ATRT.

To address this research gap, genome-scale perturbation screens serve as a powerful methodological approach to dissect genetic dependencies, context-dependent functional networks, and chemogenetic interactors in cancer cells on a single gene level [15, 16]. In particular, loss-of-function strategies using shRNA or CRISPR-Cas9-based techniques have been used across a multitude of cancer entities to reveal genetic dependencies that may provide the basis for discovery and prioritization of therapeutic targets [17, 18]. This approach might be particularly useful in malignancies with relatively stable genomes and a low abundance of targetable genetic alterations including ATRT [19].

We hypothesized that genome-wide functional screening will discover genetic dependencies that might serve as actionable molecular targets in ATRT despite their untargetable genetic profile. Thus, we employ here genome-wide CRISPR-Cas9 knockout screens. We discover a preferential sensitivity of ATRT cells to inhibition of these dependencies by targeted compounds including CDK4/6 inhibitors, independent of ATRT subgroups. Furthermore, we identify diverging cell cycle programs in ATRT, highlighted by diverse G1 phase cyclin essentiality profiles and diverse capabilities to modulate ATRT response to CDK4/6 inhibition. Additionally, we identify a previously unexplored cell type-specific role for the ubiquitin ligase substrate receptor AMBRA1 by acting as a potent tumor suppressor through regulation of factors involved in G2 and M phase progression.

## Results

### CRISPR-Cas9 knockout screens reveal genetic dependencies of ATRTs

**Fig. 1:**
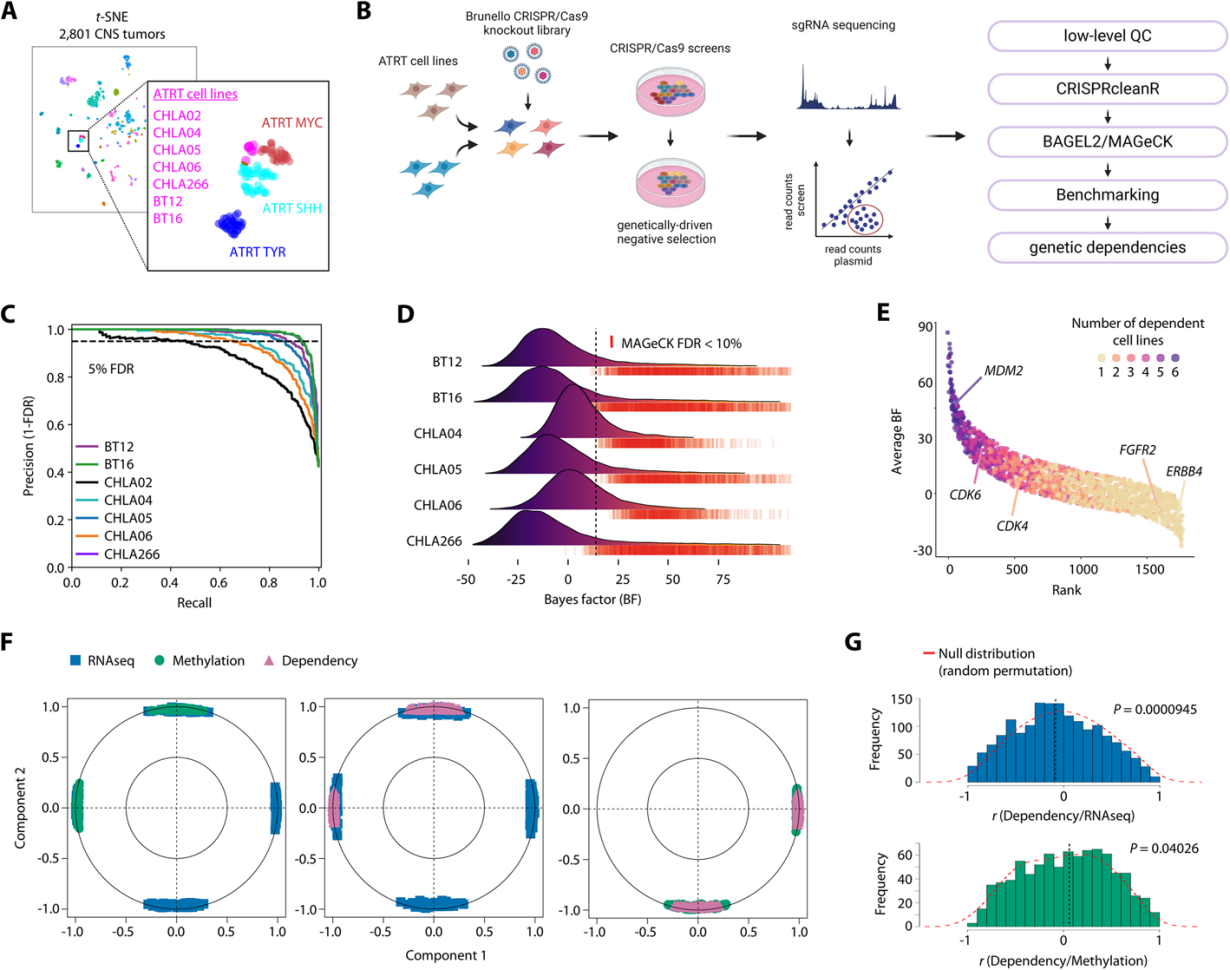
CRISPR-Cas9 knockout screens reveal genetic dependencies of ATRT. **A** t-SNE dimensionality reduction of global DNA methylation profiles from seven human ATRT cell lines (indicated in red) and a reference cohort of 2801 primary CNS tumors. **B** Overview of experimental approach using CRISPR-Cas9 knockout screens to identify genetic dependencies in ATRT cells. **C** Precision-recall-curve analyses for seven ATRT cell lines based on distribution of known essential and non-essential genes. Dashed line denotes 5% false discovery rate (FDR). **D** Ridgeline plot illustrating the distribution of gene Bayes factors for six ATRT cell lines calculated using BAGEL2. Vertical dashed line illustrates the lowest Bayes factor across cell lines at FDR < 10%. Rugs indicate genes with an FDR < 10% for depletion (neg. FDR) as determined by MAGeCK-RRA. **E** Illustration of the number of dependent ATRT cell lines for all context-specific essential fitness genes. **F** Correlation circle plots illustrating results from pairwise sPLS analyses integrating gene expression, gene promoter methylation and gene dependency. Shown are correlations of the top 100 variables with the first two components. In between the original variables, acute angles (< 90°) indicate positive correlations, while obtuse angels (> 90°) indicate negative correlations. **G** Bar graphs showing the density distributions of correlation coefficients for dependency and gene expression or dependency and gene promoter methylation of context-specific essential genes. Red dashed line illustrates the null distribution as generated by random permutation. Statistics are derived from robust rank aggregation (MAGeCK RRA) or 10-fold cross-validation (BAGEL2) (**C**, **D**), and a Wilcoxon rank sum test (**G**)

As a prerequisite for functional genomic screening, we first performed a detailed molecular profiling of a set of seven human ATRT cell lines (BT12, BT16, CHLA02, CHLA04, CHLA05, CHLA06, CHLA266; see “Methods”), providing evidence for a clear ATRT descent, including loss-of-function alterations in *SMARCB1* in all cell lines (Fig. 1A; Additional file 1: Fig. S1A,B; Additional file 2: Table S1). Of note, our integrated analyses led to distinct subgroup assignments for these seven cell lines to ATRT-SHH (CHLA02, CHLA04, CHLA05) or ATRT-TYR/MYC subgroups (CHLA06, CHLA266, BT12, BT16) (Additional file 1: Fig. S1C-H; Additional file 14), being in line with previous subgroup assignments for these lines [12]. We next performed a total of 21 genome-wide CRISPR-Cas9 knockout screens in seven ATRT cell lines, targeting 19,114 genes [20] (Fig. 1B; Additional file 3: Table S2). Our downstream bioinformatic pipeline included low-level read count-based quality control, correction for gene-independent effects, and screen performance evaluation using precision and sensitivity metrics for the classification of essential and non-essential genes (Fig. 1C; Additional file 1: Fig. S2A-D). Eighteen screens from six ATRT cell lines with high screening performance were kept for further analysis (Additional file 1: Fig. S2E-G).

We assessed gene essentiality using a combination of supervised (BAGEL2) and unsupervised (MAGeCK RRA) gene fitness classification (Fig. 1D; Additional file 3: Table S2). We identified a median of 1592 (range 866–2100) fitness genes at FDR < 10% in each cell line, and dependency profiles generated with the Brunello library were well correlated with essentialities from ATRT cell lines within the Project Achilles which is based on the Avana library (Additional file 1: Fig. S3A) [21]. Next, we masked genes that are known to be common essential across human cancer cells [22, 23], leading to the identification of a total of 1768 (range 446–1108) context-specific essential genes in ATRT cells that are associated with several pathways amenable to therapeutic intervention such as cell cycle control, DNA organization, and growth factor signaling (Additional file 1: Fig. S3B,C).

We noted that the dependency profiles for these context-specific essentials varied considerably across ATRT cell lines (Fig. 1E), and thus analyzed our screens for potential predictors of gene essentiality including genetic drivers, synthetic lethal interactions, and mRNA expression levels [24, 25]. We first used an in silico analysis to predict oncogenic mutations present in our ATRT cell lines. While this approach yielded a total of 37 potentially tumor-driving events in our cohort of ATRT cell lines (Additional file 2: Table S1), none of these were associated with a cell line-specific essentiality. In contrast, we identified several synthetic lethal dependencies [25], e.g., *TP53* wild-type ATRT cell lines being sensitive to loss of negative regulators of p53 signaling *MDM2* and *MDM4* (Additional file 1: Fig. S4A), further validating our screening approach. Due to the overall low mutational burden and stable genomes of ATRT, we reasoned that the majority of dependencies might be driven by high expression of the corresponding gene, as is true for most cancer dependencies [25]. We therefore performed sparse projection to latent structures (sPLS) in an unsupervised fashion to explore the relationship of DNA promoter methylation, gene expression, and gene dependency scores in ATRT cells [26]. Top selected features showed strong clustering among the first two components in pairwise comparisons and revealed relevant associations both in pairwise as well as multiblock comparisons (Fig. 1F; Additional file 1: Fig. S4B). Of note, while we found both positive as well as negative correlations of gene expression features with gene dependency scores, our data revealed exclusive positive correlations of promoter methylation and dependency scores, where low promoter methylation levels that lead to higher gene expression are associated with gene dependency. Direct correlation of either gene expression or promoter methylation with gene dependency scores for context-specific fitness genes revealed a significant shift of correlation coefficients towards a more negative (gene expression) or positive (methylation) distribution as compared to the null distribution (Fig. 1G). Indeed, several targets with potential clinical relevance such as *MYC*, *CDK6*, and *FGFR2* show a strong negative correlation with high gene expression leading to gene dependency (Additional file 1: Fig. S4C). Together these data suggest that context-specific essentiality in ATRT cells may be predicted to a useful extent by high gene expression which can serve as predictive molecular signature to inform biomarker-guided targeted therapies.

### CRISPR screens guide the identification of chemical dependencies in ATRT

**Fig. 2:**
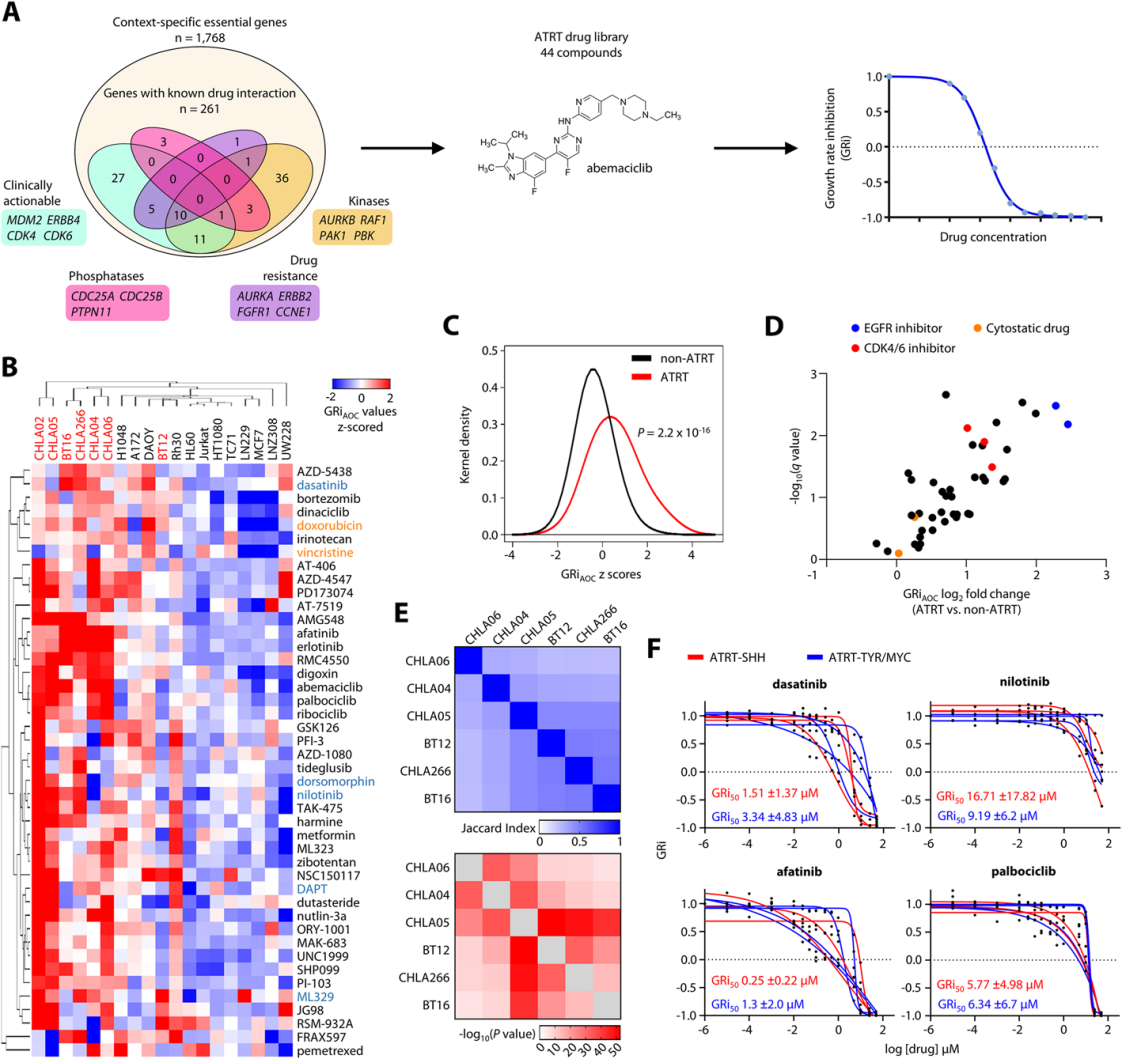
Functionally-instructed chemical dependencies in ATRT. **A** Graphical summary for the generation of a functionally-instructed drug library and drug screen analysis details. Venn diagram showing categorization of context-specific essential genes into druggable categories as determined by the Drug Gene Interaction database highlighting selected drug classes. **B** Unsupervised hierarchical clustering (1 minus Pearson correlation, average linkage) of z-scored GRiAOC values derived from a three-dose drug screen (0.01 μM, 0.1 μM, 1 μM for 72 hours) in 19 different human cancer cell lines. ATRT tumor cell lines are indicated in red. Broadly cytotoxic compounds are shown in orange, drugs previously shown to act in an ATRT subgroup-dependent manner are shown in blue. **C** Kernel density estimation and statistical comparison of z-scored GRi_AOC_ values from functionally-instructed drugs grouped by ATRT and non-ATRT cell lines. **D** Graph illustrating the log_2_ fold change in GRi_AOC_ and the corresponding q value of ATRT cell lines as compared to non-ATRT control cell lines. Selected drug classes are color coded. **E** Heat maps illustrating the Jaccard indices (top) and the corresponding significance (bottom) of pairwise intersections of context-specific essential genes. The order of the heat map was determined by unsupervised hierarchical clustering (1 minus Pearson correlation, average linkage) of the samples based on their Jaccard indices. **F** 15 point GRi dose response curve analyses for selected small molecules in ATRT-SHH (CHLA02, CHLA04, CHLA05) and ATRT-TYR/MYC (BT12, BT16, CHLA06, CHLA266) cell lines. Mean GRi_50_ values for ATRT-SHH and ATRT-TYR/MYC subgroup cell lines are shown

Since genetic dependencies might nominate potential drugs for a functionally instructed targeted therapy in ATRT, we next interrogated the Drug Gene Interaction database (DGIdb) and explored known drug-gene interactions and potential druggability of context-specific essential genes [27], thereby discovering 261 candidate genes with known drug interaction (Fig. 2A; Additional file 4: Table S3). Based on these data, we generated a functionally instructed chemical library of 44 distinct small molecule inhibitors including 37 compounds as potential inhibitors of context-specific genetic dependencies in ATRT cell lines, five compounds previously shown to be efficacious in ATRT in a subgroup-dependent manner as positive controls (dasatinib, nilotinib, dorsomorphin, DAPT, ML329) [9, 11], as well as two broadly cytotoxic agents (vincristine, doxorubicin). Using a three-dose drug screen, we determined the effects of this drug library on the viability of seven ATRT and 12 non-ATRT control cancer cell lines (Additional file 1: Fig. S5A,B; Additional file 5: Table S4), while employing growth rate inhibition (GRi) and area-over-the-curve-response curve (AOC) metrics [28] in order to account for differences in the division rate among cell lines (see “Methods”). As evidenced by unsupervised hierarchical clustering, the majority of ATRT cell lines showed a highly homogenous response to our drug library that was distinct from non-ATRT cell lines, and inhibitors with overlapping target profiles clustered together, suggesting on-target activity (Fig. 2B). Variable clustering of BT12 cells to either ATRT or non-ATRT cells at different drug concentrations might suggest distinct sensitivities of this cell line from other ATRT models (Additional file 1: Fig. S5B), and this is in line with a distinct gene expression profile in BT12 cells (Additional file 1: Fig. S7H). Overall, functionally instructed inhibitors were more potent in ATRT cell lines as compared to non-ATRT cell lines as a whole (Fig. 2C), and 14 out of 37 inhibitors displayed a significantly higher potency in ATRT cell lines compared to non-ATRT cell lines as judged by GRi_AOC_ values (Fig. 2D). Importantly, most of the drugs with a favorable activity profile in ATRT cells including EGFR and CDK4/6 inhibitors showed reduced yet considerable efficacy in non-ATRT cells (Additional file 1: Fig. S5C; Additional file 5: Table S4), showing that their activity is not exclusive to ATRT cells. While we note that ATRT and non-ATRT cell lines in our drug screen are genetically distinct and that the predominantly adult cancer types in non-ATRT lines show a higher degree of genome instability (Additional file 1: Fig. S5D), our data strongly suggest that the genetically homogenous group of ATRT cells is preferentially sensitive to our functionally instructed drug library.

While many of these functionally instructed drugs significantly inhibit the growth of ATRT cells, they might also exhibit substantial toxicity to normal cells of the nervous system, thereby limiting their potential clinical utility. To test the neurotoxicity profile of the most promising compounds in our library, we tested drugs with the most reliable efficacy in GRi across ATRT cell lines (GRi_MAX_ < 0.5 in at least 4 ATRT lines, *n* = 20) on murine post-mitotic cerebellar granule neurons and human astrocytes. Overall, 9 compounds including inhibitors of CDK4/6, MAPK, and EGF signaling did not significantly inhibit viability of normal cells (Additional file 1: Fig. S5E,F), suggesting that these classes of compounds represent chemical vulnerabilities of ATRTs with a broad therapeutic window.

ATRT subgroups were suggested to exhibit distinct chemical sensitivities [11]. We therefore re-analyzed both our genetic and chemical dependency data in this regard, focusing on differential vulnerabilities of ATRT-SHH (group 1) and ATRT-TYR/MYC (group 2) cell lines. We first used multi-set intersection analyses [29] to investigate a potential subgroup-specific, differential enrichment of context-specific genetic dependencies from our ATRT CRISPR screens. We did not observe any apparent clustering of dependency intersections associated with ATRT subgroups across any combination of ATRT cell lines (Additional file 1: Fig. S6A), and pairwise dependency set overlaps as measured by Jaccard indices and corresponding *P* values did not support a subgroup-driven enrichment of single gene dependencies in ATRTs (Fig. 2E). Furthermore, we detected a highly significant positive correlation of GRi_AOC_ values from ATRT-SHH and ATRT-TYR/MYC cell lines in our three-dose drug screen testing 44 small molecules (Additional file 1: Fig. S6B), indicating that cell models from distinct ATRT subgroups show similar sensitivity to most drugs in our library. In addition, we performed detailed GRi dose-response analyses for selected compounds from our library including EGFR and CDK4/6 inhibitors as well as dasatinib and nilotinib, both multi-targeted kinase inhibitors previously described to selectively inhibit viability of ATRT-TYR/MYC cell lines [11]. None of these drugs showed a significant difference in their ability to inhibit the growth rate of either ATRT-SHH or ATRT-TYR/MYC cell lines (Fig. 2F; Additional file 1: Fig. S6C). In summary, while our data do not provide any evidence for subgroup-specific genetic or chemical vulnerabilities in ATRT, we identify several drug classes including inhibitors of CDK4/6 and signaling pathways such as EGF and PI3K to be particularly efficacious in ATRT cells.

### CDK4 and CDK6 are distinct predictors of CDK4/6 inhibitor sensitivity in ATRT

**Fig. 3:**
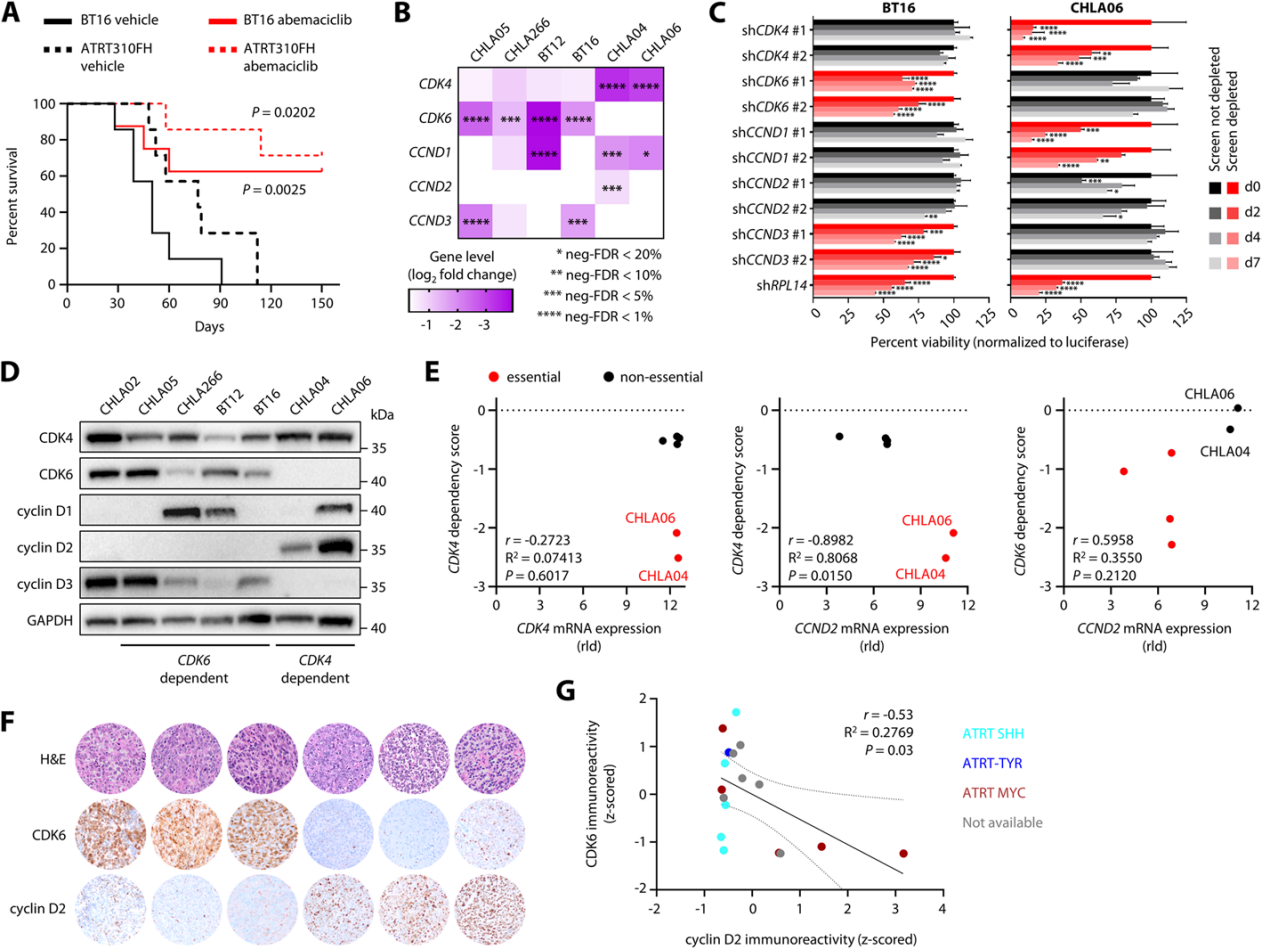
CDK4 and CDK6 are distinct predictors for CDK4/6 inhibitor sensitivity in ATRT. **A** Kaplan-Meier survival analyses of intracranial transplantation tumor mouse models (BT16 and ATRT310FH) treated daily with 75 mg/kg abemaciclib or vehicle (*n* = 7 for each condition and model), monitored for 150 days after tumor cell transplantation. **B** Heat map illustrating gene level log_2_ fold changes and corresponding FDR statistics for CDK4, CDK6, and all D-type cyclins in ATRT CRISPR knockout screens. **C** Bar graphs showing the effect of shRNA-mediated knockdown of CDK4, CDK6, CCND1, CCND2, and CCND3 in BT16 and CHLA06 cells (*n* = 3 independent experiments, *P < 0.05, **P < 0.01, ***P < 0.001, ****P < 0.0001). Control shRNAs target the pan-essential gene RPL14 or the luciferase gene for normalization. **D** Western blot analyses showing the protein expression levels of CDK4, CDK6, and D-type cyclins in the indicated ATRT cell lines. Note that profiles for CDK4 or CDK6 dependency were not available for CHLA02 cells. **E** Correlation analyses to illustrate CDK4 or CDK6 dependency prediction by *CCND2* and *CDK6* mRNA expression. Dependency for the gene on the y axis is indicated by red color. **F** Representative H&E stains and immunohistochemistry for CDK6 and cyclin D2 in tumor tissue from 6 ATRT patients (each column corresponds to one patient). **G** Correlation analysis of immunoreactivity for CDK6 and cyclin D2 in all analyzed ATRT patient tissues (*n* = 17). Dashed lines indicate the 95% confidence interval. Data are shown as mean ± SEM (**C**). Statistics are derived from a Log-rank test (**A**), robust rank aggregation (**B**), two-way ANOVA with Dunnett correction (**C**), and t tests (**E**, **G**)

Loss of *SMARCB1* in rhabdoid tumors has been associated with deregulation of the cyclin D-CDK4/6-RB axis [30–32], and CDK4/6 inhibitors were among the inhibitors with the most promising activity profile in ATRT cells. CDK4/6 inhibition in ATRT cells led to cell cycle arrest in G1 phase and displayed a strong anti-tumor effect in all ATRT models including an ATRT-SHH cell line derived from a patient-derived orthotopic xenograft (PDOX) mouse model [33] (Additional file 1: Fig. S7A-C). Additionally, ATRT cell lines showed a similar sensitivity to abemaciclib in colony formation assays as MCF7 cells (Additional file 1: Fig. S7D), an ER^+^ breast cancer line known to be highly susceptible to CDK4/6 inhibition [34], whereas other non-ATRT solid tumors showed substantially lower sensitivity to CDK4/6 inhibition. This is in line with results from our drug screen, and these results were not biased by genetic alterations within the p53 or cyclin D-CDK4/6-RB pathways previously described to modulate response to CDK4/6 inhibition [35–38] (Additional file 1: Fig. S7E). Importantly, abemaciclib treatment significantly prolonged survival in vivo in orthotopic xenograft mouse models using BT16 cells, a model for ATRT-MYC, and ATRT310FH, a PDOX model for ATRT-SHH [33] (Fig. 3A). Together, these data suggest a preferential sensitivity of ATRT cells to CDK4/6 inhibition.

We then aimed at investigating the molecular mechanisms underlying CDK4/6 inhibitor sensitivity in ATRT. All ATRT cell lines depend either on *CDK4* or *CDK6* expression in a mutually exclusive manner, a pattern recapitulated by most human cancer cell lines [25], and most require expression of at least one out of three D-type cyclins (Fig. 3B; Additional file 1: Fig. S7F). Genetic silencing of CDKs and associated D-type cyclins in both *CDK4* and *CDK6*-dependent ATRT cell lines confirmed the heterogenous dependency on distinct G1 phase cell cycle regulators as predicted by our CRISPR-Cas9 screens (Fig. 3C). We reasoned that differential expression of members of the CDK/D-type cyclin axis might be responsible for this heterogeneity, as suggested by our finding that high expression of *CDK6* may predict *CDK6* dependency (Additional file 1: Fig. S4C) and the lack of any obvious clustering of *CDK4*- and *CDK6*-dependent cell lines based on global gene expression profiling (Additional file 1: Fig. S7G,H). Indeed, protein expression of CDK4, CDK6, and associated D-type cyclins revealed a striking heterogeneity in ATRT cells, which was highly reminiscent of the corresponding dependency profiles of these genes (Fig. 3D; Additional file 15). We identified *CCND2*/cyclin D2 and *CDK6*/CDK6 expression as predictors of *CDK4*- and *CDK6*-dependent cell lines, respectively (Fig. 3D,E). While *CDK4*/CDK4 levels were not associated with *CDK4* essentiality, *CDK4* dependency scores showed strong negative correlation with *CCND2*/cyclin D2 expression (i.e., ATRT cells that dependent on *CDK4* show high *CCND2*/cyclin D2 expression), with *CDK4*-dependent cells showing no detectable CDK6 protein expression. In contrast, *CDK6* dependency scores showed strong negative correlation with *CDK6*/CDK6 expression and were positively correlated with *CCND2*/cyclin D2 levels. Notably, these correlations are conserved across human cancer cell lines, where *CDK6* expression is positively correlated with *CDK4* dependency scores, while showing strong negative correlation with *CDK6* dependency scores [25]. We validated this expression pattern in 17 human ATRT patient samples by immunohistochemistry stainings for CDK6 and cyclin D2 (Fig. 3F). Indeed, we observed a negative correlation of CDK6 and cyclin D2 protein expression, where low CDK6 expression correlated with high cyclin D2 expression, and vice versa (Fig. 3G). Taken together, these data suggest that ATRT cells employ distinct cell cycle programs and diverging usage of G1 phase cyclins and CDKs, and this predisposes to differential dependencies for these cell cycle regulators.

### G1 phase cyclins are diverging regulators of response to CDK4/6 inhibition and cell cycle progression in ATRT cells

**Fig. 4:**
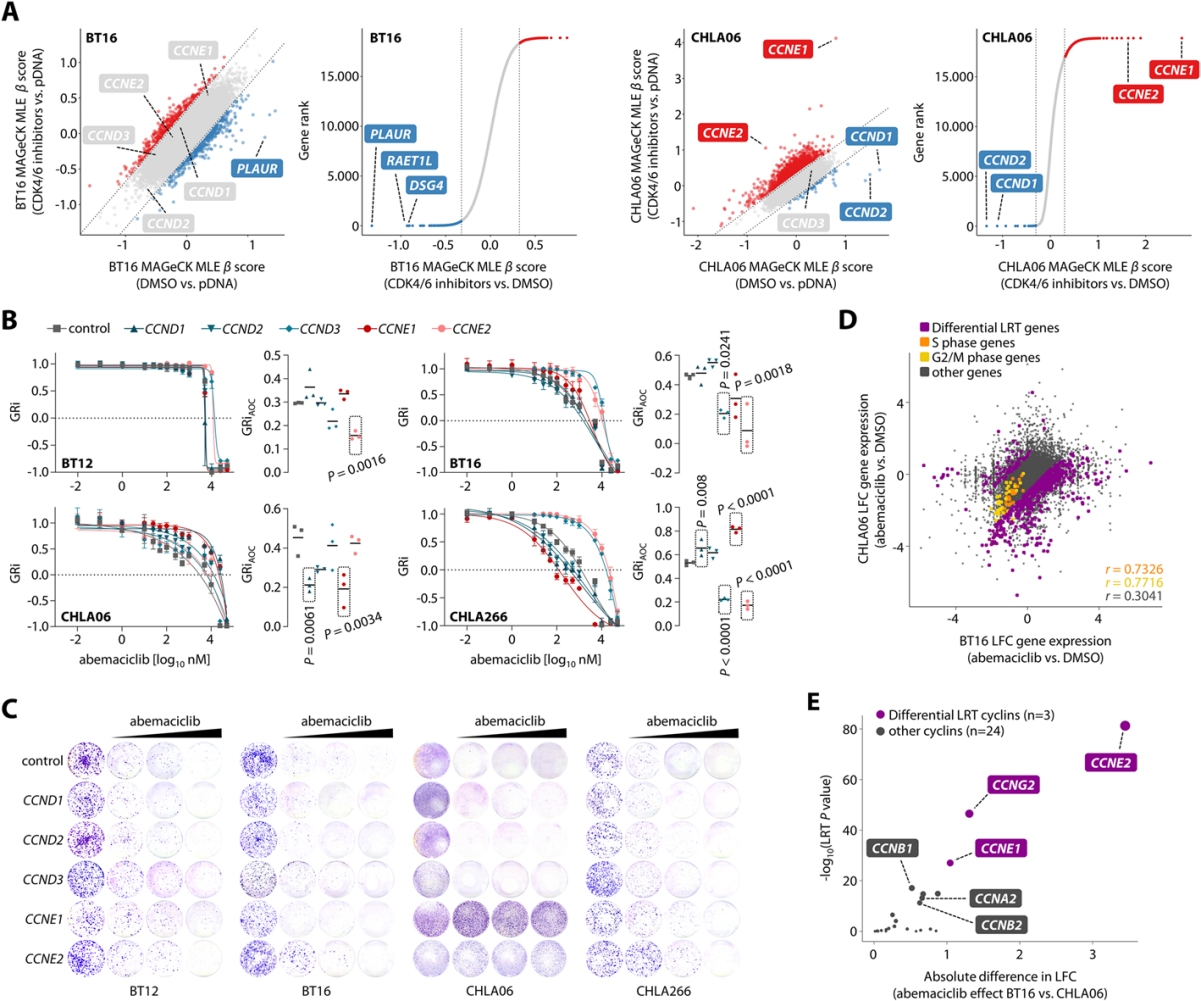
G1 phase cyclins are diverging regulators of response to CDK4/6 blockade and cell cycle progression in ATRT cells. **A** Scatter and rank plots for screening data from CRISPR-dCas9-VP64 chemogenetic screens. BT16 (left) and CHLA06 cells (right) were treated with the CDK4/6 inhibitors (abemaciclib or palbociclib) or DMSO, and MAGeCK MLE was used to model common differences in CDK4/6 inhibitor treated screens as compared to either the DMSO control or the plasmid DNA reference (pDNA). **B** GRi dose response curve analyses for ATRT cells overexpressing G1 phase cyclins and comparison of GRi_AOC_ values. Statistically significant differences in GRi_AOC_ values are highlighted. **C** Analyses of clonogenic survival of ATRT cell lines under increasing concentrations of abemaciclib (200 nM to 800 nM). See Additional file 1: Fig. S8E for statistics. **D** Scatter plot illustrating common and differential gene expression changes in BT16 and CHLA06 cells upon CDK4/6 blockade. Common suppression of S and G2/M phase-associated genes as a result of G1 phase arrest is indicated in orange/yellow. Genes with differential gene expression changes in both cell lines as determined by likelihood ratio test (LRT) are highlighted in magenta. **E** Volcano plot associating the absolute difference in gene expression changes between BT16 and CHLA06 cells and its corresponding LRT P value for all annotated cyclins. Data are shown as mean ± SD (**B**). Statistics are derived from maximum likelihood estimation (**A**), one-way ANOVA with Dunnett correction (**B**), and a likelihood ratio test (**D**, **E**)

We next aimed to test whether diverging cell cycle programs in different ATRT cell lines result in differential modulation of CDK4/6 inhibition, and thus performed chemogenetic CRISPR screens (Additional file 1: Fig. S8A; see “Methods”). We first investigated the role of gene activations for potential modulation of drug response in a CRISPR-dCas9-VP64 approach using two distinct ATRT cell lines based on their differential dependency on either *CDK4* (CHLA06) or *CDK6* (BT16). Data from activation screens for two distinct CDK4/6 inhibitors (abemaciclib and palbociclib) strongly correlated by cell type, but not by individual CDK4/6 inhibitors (Additional file 1: Fig. S8B; Additional file 6: Table S5). In fact, activation of E-type cyclin genes, in particular *CCNE1*, conferred resistance to CDK4/6 inhibition in CHLA06 cells but not in BT16 cells (Fig. 4A). Additionally, these screens identified D-type cyclins as positive regulators of cell cycle progression only in CHLA06 cells but not in BT16 cells. Contrasting these results, all ATRT cell lines relied on at least one D-type cyclin for survival while E-type cyclins were uniformly dispensable (Additional file 3: Table S2), illustrating further functional heterogeneity with regard to pure cell survival and cell cycle progression. Accordingly, overexpression of G1 phase cyclins showed highly diverging effects on the proliferation of ATRT cells (Additional file 1: Fig. S8C,D; Additional file 16), suggesting that cell cycle progression is regulated differently in ATRT cells by a distinct combination of G1 phase cyclins in ATRT cells.

We next assessed the capacity of G1 phase cyclin overexpression to confer resistance to CDK4/6 blockade using GRi metrics of dose-response data and clonogenic survival assays. Activation of *CCNE1*, and to a lesser extent *CCND1* while not being a clear screen hit for a buffering effect, conferred resistance in CHLA06 but none of the other cell lines (Fig. 4B,C; Additional file 1: Fig. S8E). In contrast, all other ATRT cell lines were amenable to activation of *CCND3* and *CCNE2* to potentially confer resistance to CDK4/6 inhibition. Of note, activation of *CCNE2* also decreased sensitivity of CHLA06 cells to CDK4/6 blockade, but to a lesser extent than *CCNE1*. We hypothesized that these differences in sensitivity profiles might be related to gene expression changes downstream of CDK4/6 inhibition in the individual ATRT cell lines, based on the idea that re-activation of drug-suppressed genes might confer resistance. Global gene expression profiling for two ATRT cell lines under abemaciclib treatment revealed common expression changes associated with G1 phase arrest, but also significant differences in gene regulation (Fig. 4D; Additional file 1: Fig. S8F,G). Among the top differentially affected cyclins by abemaciclib treatment, cell type-specific downregulation of *CCNE1* expression in CHLA06 cells was associated with resistance when re-activated, while endogenous levels of *CCNE1* in BT16 cells increased by CDK4/6 blockade and overexpression failed to confer resistance (Fig. 4E; Additional file 1: Fig. S8H). In contrast, *CCNE2* expression was decreased in both ATRT cell lines under abemaciclib treatment and overexpression conferred resistance in both cell lines. Gene expression changes for D-type cyclins upon CDK4/6 blockade were similar across ATRT cell lines, with the exception that *CCND2* was only upregulated in the context of detectable baseline expression. Furthermore, *CCND1* and *CCND2* were exclusively upregulated, whereas *CCND3* showed a significant but minor decrease in expression upon CDK4/6 inhibition. Together, overexpression of distinct E-type cyclins conferred resistance in a cell context-specific manner, and this degree of resistance was much more pronounced than that conferred by overexpression of D-type cyclins. Mechanistically, resistance conferred by E-type cyclins was associated with gene re-activation, while resistance conferred by cell line-specific overexpression of distinct D-type cyclins was not necessarily associated with re-activation of D-type cyclin genes after CDK4/6 blockade. These data further provide evidence for distinct cell cycle programs and particularly different regulation of cell cycle progression in ATRT cells as a potential mechanism of resistance to CDK4/6 inhibition.

### AMBRA1 acts as a context-dependent tumor suppressor in ATRT

**Fig. 5:**
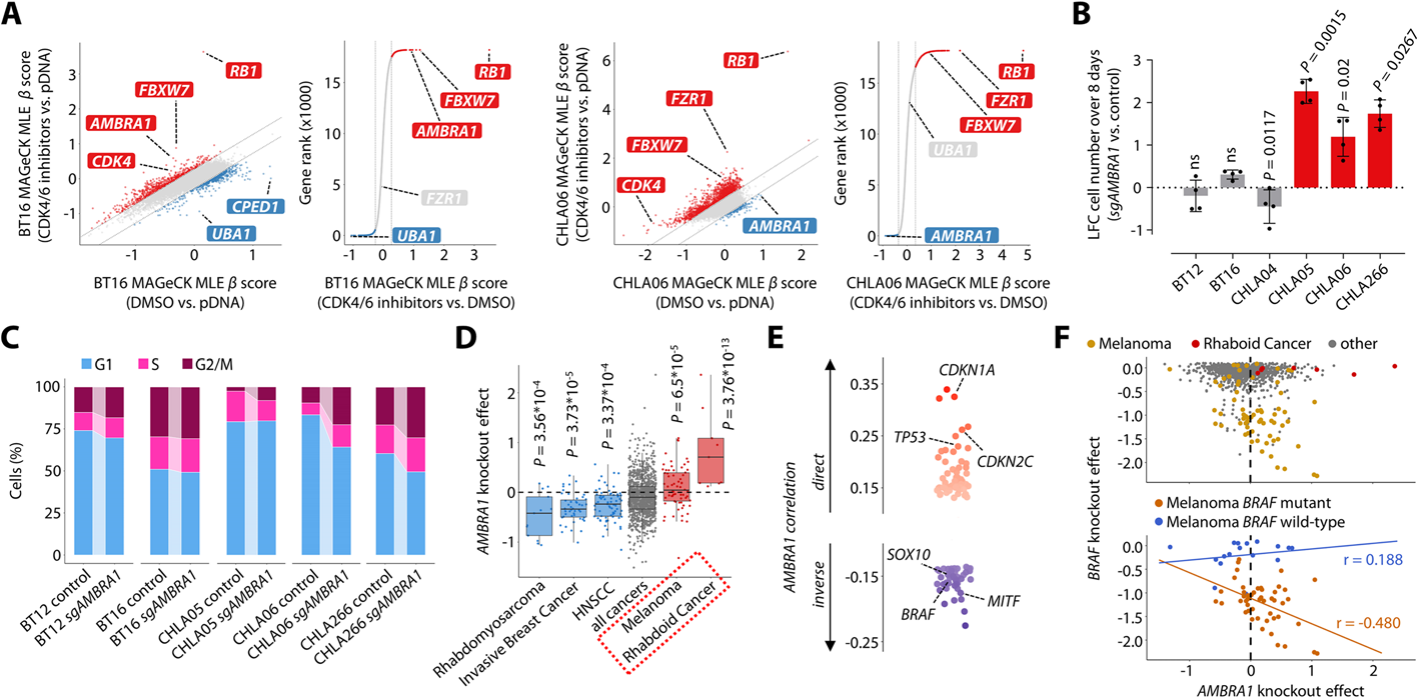
AMBRA1 is a context-dependent tumor suppressor. **A** Scatter and rank plots for screening data from CRISPR-Cas9 knockout drug screens. MAGeCK MLE was used to identify common screen hits in BT16 (left) and CHLA06 cells (right) that were treated with CDK4/6 inhibitors (abemaciclib or palbociclib) and DMSO by comparing drug screens to either the DMSO control or the plasmid DNA reference (pDNA). **B** Effect of loss of *AMBRA1* on the proliferation of ATRT cells as measured by log_2_ fold change in cell number over 8 days for *AMBRA1* knockout cells compared to control cells. **C** Alluvial plots illustrating changes in cell cycle distribution of ATRT cells upon loss of *AMBRA1*. See Additional file 1: Fig. S9G for statistics. **D** Effect of *AMBRA1* knockout in 1150 human cancer cell lines from DepMap. Boxes illustrate primary diseases in which the knockout effect of *AMBRA1* significantly differed from all other cell lines. HNSCC: Head and Neck Squamous Cell Carcinoma. **E** The top 100 pre-computed genetic associations for *AMBRA1* in DepMap. Selected genes that show a skewed gene effect distribution across human cancer cell lines are indicated. **F** Correlation of *AMBRA1* and *BRAF* gene knockout effects in DepMap, highlighting melanoma and rhabdoid cancer cell lines. Data are shown as mean ± SD (**B**). Statistics are derived from maximum likelihood estimation (**A**), and t tests (**B**, **D**)

In parallel to gain-of-function chemogenetic screens described above, we performed genome-wide screening to identify loss-of-function alterations that might modify response to CDK4/6 blockade as well as to delineate general regulators of cell cycle progression in ATRT cells (Additional file 1: Fig. S9A; Additional file 7: Table S6). Again, correlation analyses of screening data for distinct CDK4/6 inhibitors in two ATRT cell lines suggested a cell line-specific response to CRISPR-Cas9 mediated gene knockouts under drug treatment (Additional file 1: Fig. S9B). However, the top hits modulating drug response were shared in both cell lines, with loss of *RB1* and *FBXW7* being among the top scoring genes to confer resistance to CDK4/6 blockade (Fig. 5A) as expected [35, 39]. The top differential hit across both cell lines was the autophagy-related gene *AMBRA1*, which was predicted to act as a strong negative regulator of cell cycle progression only in CHLA06 cells. AMBRA1 was recently described as a potent tumor suppressor, which regulates the stability of D-type cyclins and S phase entry, and loss of *AMBRA1* has been suggested to decrease sensitivity to CDK4/6 blockade [40–42]. Our screen did not suggest a major role for AMBRA1 in ATRT cells in modulating response to CDK4/6 inhibitors per se, and functional validation did not provide any evidence that loss of *AMBRA1* confers resistance to CDK4/6 blockade in ATRT cells (Additional file 1: Fig. S9C-F). In contrast, loss of *AMBRA1* selectively enhanced proliferation in a subset of ATRT cell lines (Fig. 5B), and this was paralleled by a strong increase in G2/M rather than S phase cells upon loss of *AMBRA1* in a cell line-specific manner (Fig. 5C; Additional file 1: Fig. S9G).

In order to put these findings into a broader context, we interrogated the DepMap (https://depmap.org/) for knockout effects and potential genetic interactions of *AMBRA1* across 1150 human cancer cell lines. Indeed, while knockout of *AMBRA1* did not affect the vast majority of cell lines, individual primary diseases showed a unique response pattern, and knockout effects in melanoma and rhabdoid cancer cell lines suggested a tumor suppressive role for AMBRA1 (Fig. 5D; Additional file 1: Fig. S10A). In line with these data, we found a strong direct correlation of *AMBRA1* with several tumor suppressors including *TP53* across DepMap, while inverse correlation was seen for selectively essential transcription factors defining neural crest (*SOX10*) and melanocyte (*MITF*) lineage (Fig. 5E). In melanoma, tumor suppressive activity of AMBRA1 was correlated with oncogenic *BRAF* signaling (Fig. 5F), corroborating previous results [43]. In line with that, genetic alterations of both *AMBRA1* and *BRAF* are prevalent in melanoma (Additional file 1: Fig. 10B), while no *AMBRA1* alterations have been identified in ATRT so far [9, 11]. Interestingly, even though rhabdoid tumors have been suggested to potentially derive from neural crest cells similar to melanocytes [44–46], neither rhabdoid cell lines from DepMap nor ATRT cell lines from our study were dependent on *SOX10* or *MITF* (Additional file 1: Fig. S10C,D). Furthermore, while context-dependent tumor suppressive activity of AMBRA1 has been associated with oncogenic KRAS signaling in lung adenocarcinoma [42], we did not find any correlation of *KRAS* and *AMBRA1* in tumors which show recurrent oncogenic KRAS mutations (Additional file 1: Fig. 10E). Together, these analyses reveal context-specific tumor suppressor activity of AMBRA1 which might be related to descent from the neural crest lineage.

### AMBRA1 tumor suppressor activity is associated with regulation of G2/M phase mediators

**Fig. 6:**
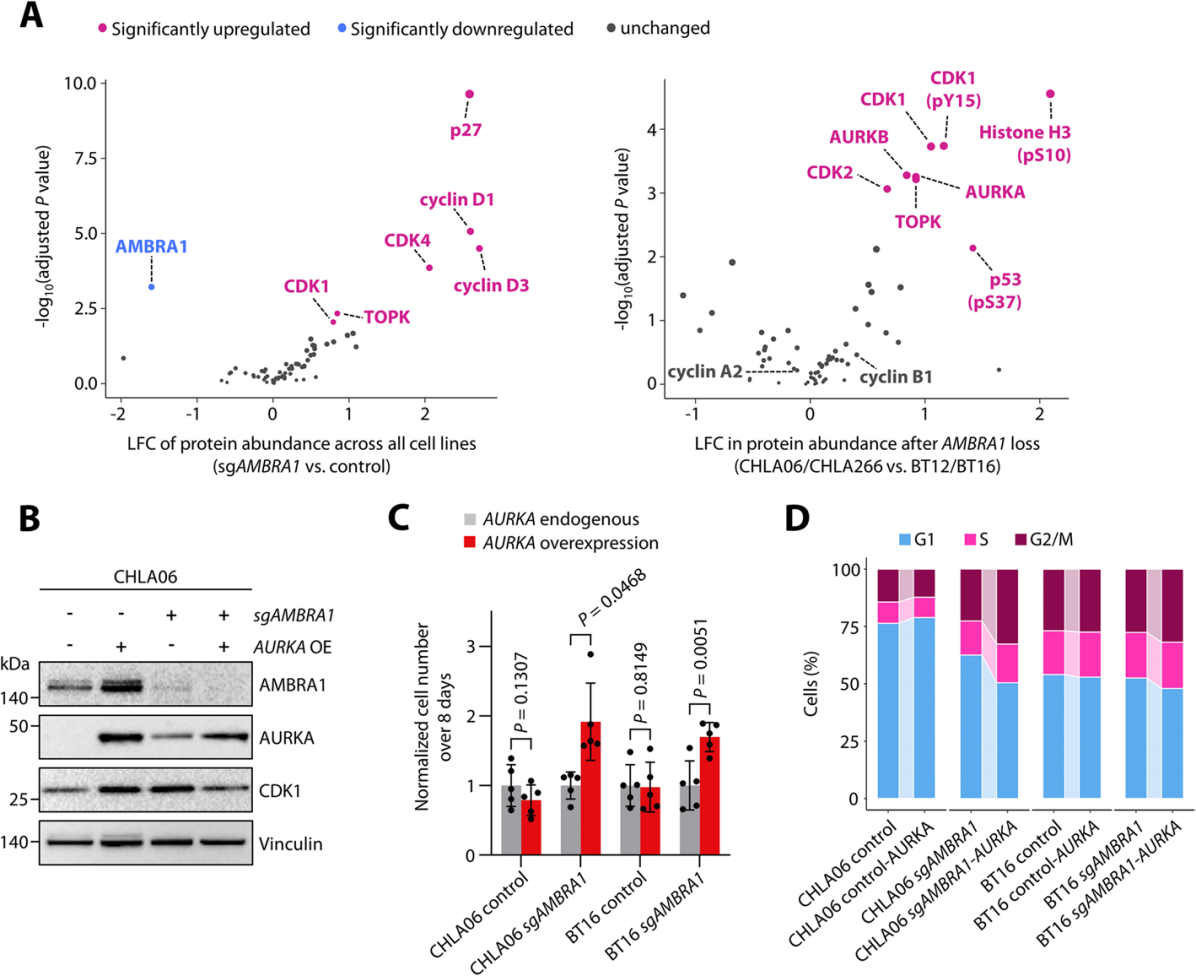
AMBRA1 tumor suppressor activity is associated with regulation of G2/M phase mediators. **A** Volcano plots showing changes in protein levels for 39 cell cycle-associated regulators in ATRT cells upon loss of *AMBRA1* as assessed by DigiWest. Left, common changes in protein levels across four ATRT cell lines upon *AMBRA1* knockout. Right, context-specific changes in protein levels in *AMBRA1* responder (CHLA06 and CHLA266) as compared to *AMBRA1* non-responder ATRT cell lines (BT12 and BT16) upon loss of *AMBRA1*. **B** Western blot analyses for control and *sgAMBRA1* CHLA06 cells with or without overexpression of *AURKA* (*AURKA* OE). Note AURKA and CDK1 overexpression driven by loss of *AMBRA1* alone. **C** Effect of gain of AURKA on the proliferation of ATRT cells on control and *sgAMBRA1* background. D Effect of gain of *AURKA* on cell cycle phase distributions of control and *sgAMBRA1* ATRT cells. Data are shown as mean ± SD (**B**). Statistics are derived from and Welch’s t tests (**A**), and from paired t tests (**C**)

Since tumor suppressive activity of AMBRA1 has been attributed to its role as substrate receptor for a cullin4-RING E3 ubiquitin ligase (CRL4) [40–42], we next aimed to delineate changes in protein levels of cell cycle regulators upon loss of *AMBRA1* in ATRT cells with clear AMBRA1-associated tumor suppressive activity (hereafter referred to as AMBRA1 responders), and compare those to ATRT cells where loss of *AMBRA1* does not show any proliferative or cell cycle-associated effects (hereafter referred to as AMBRA1 non-responders). Employing a bead-based western blot system [47] with a total of 59 distinct antibodies recognizing unmodified and phosphorylated residues for a total of 39 cell cycle regulators, we confirmed significant reduction of AMBRA1 protein levels following CRISPR-Cas9-mediated knockout and concomitant increase of G1 phase regulators such as D-type cyclins, CDK4, and p27 across all ATRT cells (Fig. 6A; Additional file 1: Fig. S11A-C; Additional file 8: Table S7) as expected [42]. In particular, when comparing changes in protein abundance in AMBRA1 responder to AMBRA1 non-responder cell lines, we found that AMBRA1 responder cell lines showed a significant upregulation of cell cycle regulators upon loss of *AMBRA1* that are known to act during G2 and M phase of the cell cycle, including AURKA, AURKB, and their downstream target histone H3-pS10 (Fig. 6A; Additional file 1: Fig. S11D). Of note, cyclin A2 and cyclin B1 levels were not significantly altered in this comparison, suggesting that upregulation of G2/M phase regulators in AMBRA1 responder cells is not due to an increase in G2/M phase cells per se, but instead might have functional relevance for the context-dependent tumor suppressive activity of AMBRA1 in those cells. These data suggest a model in which AMBRA1, beyond previously described actions on D-type stability alone, acts as a tumor suppressor by simultaneously regulating several cell cycle regulators at once. Consistent with this model, overexpression of *AURKA* enhanced proliferation both in *AMBRA1*-deleted AMBRA1 responder (CHLA06) as well as non-responder (BT16) cells, while this was not the case in corresponding control cells (Fig. 6B,C; Additional file 17). Together, stabilization of D-type cyclins as a result of *AMBRA1* loss in combination with *AURKA* overexpression led to an increase of G2/M phase cells in both CHLA06 and BT16 cells (Fig. 6D).

### CRL4^AMBRA1^ activity regulates G2/M phase mediators

**Fig. 7:**
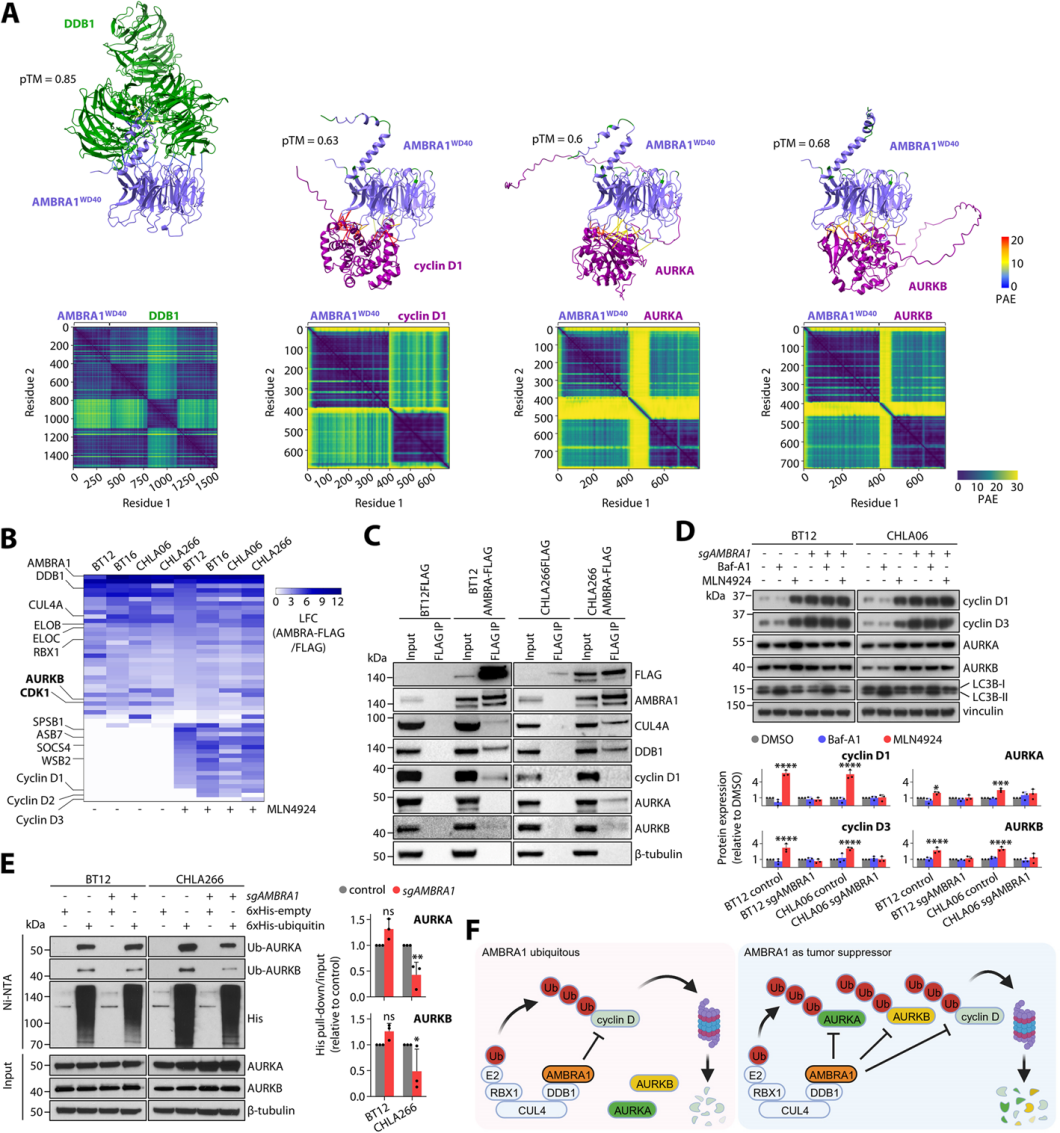
AMBRA1 regulates ubiquitin-dependent degradation of aurora kinases in a context-dependent manner. **A** Complex structure prediction using AlphaFold2_mmseqs2 for AMBRA1^WD40^ with DDB1, cyclin D1, AURKA or AURKB. Top, cartoon illustration of predicted heteromeric protein complexes. Interchain AlphaFold2 contacts (< 8 Å) are shown as straight lines colored by predicted alignment error (PAE). AMBRA1 residues implicated in DDB1 binding are highlighted in green. Predicted template modeling scores (pTM) are indicated. Bottom, pairwise PAE scores for all protein complexes. **B** Heat map showing log_2_ fold changes of label-free quantification values from FLAG affinity purification and mass spectrometry detection in AMBRA-FLAG versus FLAG expressing ATRT cells. Cells were investigated with and without treatment of the CRL inhibitor MLN4924. **C** Co-immunoprecipitation analyses followed by western blot for selected potential AMBRA1 interactors and substrates. Previously identified interactors and substrates of AMBRA1 (CUL4A, DDB1, and cyclin D1) were included as controls. **D** Top: Immunoassays of cyclin D1, cyclin D3, AURKA, and AURKB for BT12 and CHLA06 cells, both in wildtype and *AMBRA1*-knockout conditions. Cells were treated with DMSO, 0.4 μM Baf-A1, or 1 μM MLN4924 for 4 h (cyclin D) or 12 h (aurora kinases). Increase in LC3B-II levels was used as a validation of autophagy inhibition. Bottom: Quantification of protein expression levels relative the corresponding DMSO control conditions. **E** Left: Immunoassays from His pull-down experiments for BT12 and CHLA266 cells, both in wildtype and *AMBRA1*-knockout conditions, transfected either with 6xHis-empty or 6xHis-tagged ubiquitin 48 h prior to pull-down. Right: Quantification of AURKA and AURKB ubiquitylation relative to total protein levels. F Model of context-dependent, CRL4^AMBRA1^-associated blockade of cell cycle regulators via degradation by the ubiquitin-proteasome system. Data are shown as mean ± SD (**D**, **E**). Statistics are derived from two-way ANOVA tests with Dunnett’s (**D**) or Sidak’s correction (**E**). *P < 0.05, **P < 0.01,***P < 0.001, ****P < 0.0001

We hypothesized that upregulation of G2/M phase mediators upon loss of *AMBRA1* might be linked to loss of CRL4^AMBRA1^ activity similar to the mechanism driving the increase of D-type cyclins. Heteromeric protein complex structure prediction using ColabFold, an extension of the AlphaFold2 algorithm [48, 49], showed highly similar complex formations for the AMBRA1^WD40^ domain, previously shown to be important for DDB1 binding [50], with all G2/M phase regulators upregulated upon *AMBRA1* loss (Fig. 7A; Additional file 1: Fig. S12A). Of note, model accuracy estimates were higher for substrates shown to be affected by loss of *AMBRA1* (pTM range 0.68–0.59) than estimates for cyclin genes unaffected by ablation of *AMBRA1* (pTM=0.54 for cyclin A2 and cyclin B1) (Additional file 1: Fig. S12B). For all potential substrates, strong interaction is predicted for the cyclin D1-binding interface of the AMBRA1^WD40^ domain, residing contralaterally to the DDB1 binding side. To strengthen these findings, we performed affinity purification coupled with mass spectrometry analyses to identify proteins interacting with AMBRA1 in ATRT cells (see “Methods”). We found core components of CRL4 as well as CRL2/5 to be among the top interactors of AMBRA1 across all cell lines (Fig. 7B; Additional file 1: Fig. S12C,D; Additional file 9: Table S8). Inhibition of CRLs strongly increased interaction of AMBRA1 with D-type cyclins, as expected for this ubiquitin ligase substrate interaction, as well as a wide selection of CRL2/5 substrate receptors, further providing evidence for a previously described CRL cross-regulation [51]. Of note, AURKB and CDK1, two regulators shown to be upregulated upon loss of *AMBRA1*, did interact with AMBRA1 in this initial experiment. However, this interaction did not seem to increase upon CRL inhibition as suggested for a ubiquitin ligase substrate. We noted that this experiment did not detect some expected interactions of D-type cyclins and AMBRA1 altogether (e.g., BT12 cells), indicating that AMBRA1 interactions in particular with substrates for degradation are highly dynamic and potentially short lived. We therefore validated our mass spectrometry approach by performing co-immunoprecipitation followed by western blot analyses (Fig. 7C; Additional file 18). Interactors such as CUL4A and DDB1 co-immunoprecipitated invariably with AMBRA1-FLAG. However, cyclin D1 again showed variable precipitation across MLN4924-treated ATRT cell lines. Of note, interaction of AURKA with AMBRA1 was only seen in AMBRA1 responder cell line CHLA266 but not in BT12 cells, and AURKB also showed a weak signal only in CHLA266 cells.

Based on our results so far, we hypothesized that AMBRA1 may directly interact with aurora kinases in a cell context-specific manner. Thus, we aimed at investigating the mechanism of the degradation process. Acute inhibition of all CRLs, but not inhibition of autophagy, increased levels of D-type cyclins and aurora kinases in both AMBRA1 responder and non-responder cell lines (Fig. 7D; Additional file 18). Also, cyclin D and aurora kinase levels did not further accumulate in *AMBRA1* knockout cells upon CRL inhibition. Thus, in AMBRA1 responder cell lines, aurora kinase stability seems to be regulated by CRL4^AMBRA1^ activity, similarly to D-type cyclins. While *AMBRA1* knockout is not sufficient to increase aurora kinase levels in AMBRA1 non-responder lines (Fig. 6D), surprisingly loss of *AMBRA1* still impeded the accumulation of aurora kinase levels induced by CRL inhibition in these cells. These data suggest that the stability of aurora kinases in AMBRA1 non-responder cell lines is regulated by a cullin-RING ubiquitin ligase other than CRL4^AMBRA1^, while indicating that there might be crosstalk between CRL4^AMBRA1^ and the CRL degradation pathway in these cells as well. Only in AMBRA1 responder cell lines, however, loss of *AMBRA1* clearly reduced levels of ubiquitylated AURKA and AURKB (Fig. 7E; Additional file 18). In AMBRA1 non-responder cell lines, on the other hand, AMBRA1 status did not affect the ubiquitylation of aurora kinases.

Together, our data provide evidence that AMBRA1 acts as a context-specific tumor suppressor in ATRT cells, where tumor suppressive activity is associated with negative regulation of mitotic regulators. While D-type cyclins are degraded by CRL4^AMBRA1^ activity regardless of the cellular context, AMBRA1 facilitates the context-dependent degradation of aurora kinases, and potentially other G2/M phase regulators, by substrate ubiquitylation and proteasomal degradation (Fig. 7F).

### Synthetic lethality of AURKA and CDK4/6 inhibition upon loss of AMBRA1

**Fig. 8:**
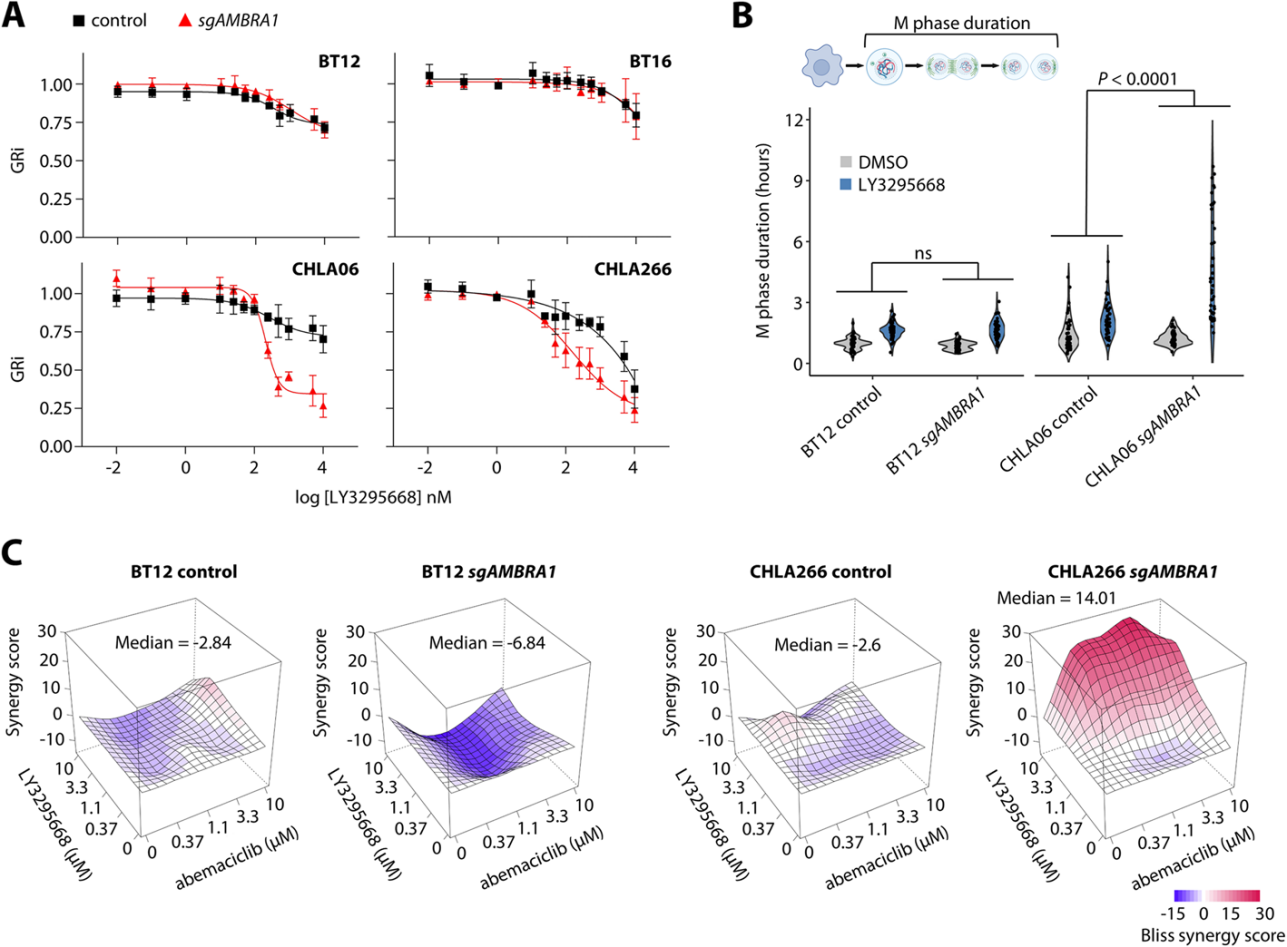
Inhibition of AURKA is synthetic lethal upon oncogenic loss of *AMBRA1*. **A** Dose response analyses for the AURKA inhibitor LY3295668 in AMBRA1 proficient and knockout ATRT cells. **B** Duration of mitosis in BT12 and CHLA06 cells, both AMBRA1 proficient and knockout cells, treated with 200 nM LY3295668. **C** Drug synergies as determined by the Bliss model for the combination of the CDK4/6 inhibitor abemaciclib and the AURKA inhibitor LY3295668 in AMBRA1 proficient and knockout ATRT cells. Data are shown as mean ± SD (**B**, **D**). Statistics are derived from paired t test (**B**), and two-way ANOVA test for interaction (**E**)

Our data highlighted an unanticipated tumor suppressive role of AMBRA1 mediated by the regulation of G2/M phase mediators in combination with D-type cyclins, suggesting that corresponding cells might be particularly dependent on these mitotic regulators. In fact, upregulation of AURKA upon loss of *AMBRA1* in ATRT responder cells strongly sensitized cells to AURKA inhibition, while *AMBRA1* status did not affect sensitivity towards AURKA inhibition in AMBRA1 non-responder cells (Fig. 8A). We reasoned that gain of mitotic factors enforced a higher proliferation rate, but might also render those cells dependent on high activity of those regulators to progress through M phase. In line with this hypothesis, prolongation of mitosis by AURKA inhibition was significantly associated with *AMBRA1* status only in AMBRA1 responder cells, with loss of *AMBRA1* significantly delaying M phase progression compared to the respective control cells (Fig. 8B; Additional files 10–13: Movies S1-4). Simultaneous blockade of cell cycle progression in G1 and G2/M phase using CDK4/6 and AURKA inhibitors showed synergistic interaction only in AMBRA1 responder cells with an *AMBRA1* knockout (Fig. 8C), further suggesting that tumor cells in which AMBRA1 fulfills a tumor suppressive role might be particularly sensitive to AURKA inhibition. Together, our findings indicate a multi-level regulation of cell cycle progression by AMBRA1. Mechanistically, regulation of protein levels of D-type cyclins in G1 and other factors such as aurora kinases at later stages implicate AMBRA1 as a critical regulator of the cell cycle, and context-specific ability of AMBRA1 to regulate these factors simultaneously might provide the basis for context-specific tumor suppressive activity.

## Discussion

In this study, we applied a functional genomics approach to ATRT, an incurable pediatric brain tumor, in order to define genetic vulnerabilities that can guide targeted therapies for this tumor entity. We here provide evidence for a particular sensitivity of ATRT to CDK4/6 inhibition, and investigate genetic modulators of response to CDK4/6 inhibitors. Surprisingly, our study reveals a very interesting and novel context-specific tumor suppressive activity of the autophagy regulator AMBRA1 in ATRT that depends on the negative regulation of G2/M phase mediators.

Our study provides an unprecedented view of genetic dependencies in ATRT at genome scale and illustrates various molecular mechanisms underlying tumor cell-specific dependencies. Small molecule inhibitor screening revealed a striking sensitivity of ATRT cells over non-ATRT cells to functionally instructed compounds based on genome-wide CRISPR-Cas9 screens. Among others, these include several classes of inhibitors that did not show any neurotoxicity in our study and present manageable toxicity profiles in the clinic for CDK4/6 [52–54] and EGFR inhibitors [55, 56], while others such as MAPK inhibitors show promising results in preclinical research [57, 58]. Of note, the dependencies identified by CRISPR screening and corresponding chemical vulnerabilities in our study did not show any particular association with epigenetically defined ATRT subgroups, contrasting previous studies [11, 59]. Yet, these studies relied on relative viability assays which can be largely confounded by the cell division rate of the investigated in vitro model systems [28, 60]. This might be of particular importance when using ATRT cell lines as these cells present a wide range of doubling times (range of 38 h to 135 h for ATRT cell lines in this study). Our analyses using growth rate inhibition drug dose-responses are in line with intersection analyses of genetic dependencies from ATRT cells, which did not provide any evidence for subgroup-specific enrichment. Together, these data suggest that chemical vulnerabilities in ATRT are a result of entity-specific molecular mechanisms, rather than a consequence of age and epigenetic or transcriptional features defining ATRT subgroups in previous studies. Yet, all these studies including ours are limited by the number of available cell lines representing individual ATRT subgroups, and novel model systems are warranted to further examine potential subgroup-specific differences.

CDK4/6 inhibitors were among the top compounds differentially affecting viability of ATRT cells as compared to non-ATRT cells. In general, *TP53* wild type tumors are predicted to show increased sensitivity to CDK4/6 inhibition as a result of p53-mediated upregulation of the cell cycle inhibitor p21 and downstream inhibition of CDK2 [61, 62], being well in line with findings of p53 pathway deregulation in patients treated with CDK4/6 inhibitors [35, 37]. However, in our collection of cell lines used for drug screening, we did not find evidence for a potential bias of increased sensitivity for ATRT cell lines based on p53 pathway deregulation. On the other hand, preferential sensitivity of ATRT cells to CDK4/6 blockade might be due to deregulation of the D-type cyclin-CDK4/6-RB1 axis downstream of loss of *SMARCB1 * [32, 63, 64]. While these previous studies have focused on the role of cyclin D1 in rhabdoid tumors, our analyses shed new light on the functional diversity of distinct D-type cyclins and associated CDKs in ATRT to drive cell cycle progression and possibly response to CDK4/6 blockade. In fact, ATRT cells express a variable set of at least one D-type cyclin, and dependency for these cyclins strongly correlated with their expression. In contrast, E-type cyclins were uniformly expressed in all ATRT cells and did not represent a dependency in any of the ATRT cells investigated, arguing that ATRT cells strictly rely on the D-type cyclin-CDK4/6-RB1 axis to progress from G1 to S phase of the cell cycle. A strong correlation of cyclin D expression and dependency has been suggested before, together with lineage-specific associations of CDK6 with cyclin D3 in hematopoietic and CDK4 with cyclin D1 in solid tumor cell lines [25]. *CDK4* and *CDK6* are mutually exclusive dependencies in ATRT, and best correlation of *CDK4* dependency was seen for *CCND2* expression in vitro and in vivo. These data further illustrate the heterogeneity of programs that drive cell cycle progression in single tumor entities which extents obvious lineage associations [65].

In line with the uniform dependency on the D-type cyclin-CDK4/6-RB1 axis to progress through the cell cycle, loss of *RB1* rescued cells from CDK4/6 blockade, but gain-of-function for G1 phase cyclins was again much more heterogenous in terms of their ability to confer resistance. E-type cyclins scored as top hits conferring resistance to CDK4/6 blockade after overexpression, being well in line with observations of E-type cyclin overexpression in preclinical models of acquired resistance to CDK4/6 inhibitors as well as resistant biopsies [35, 66]. Of note, resistance by gain of *CCNE1* or *CCNE2* expression was associated with re-activation after CDK4/6 inhibition, illustrating a cell line-specific adaptation of G1 phase cyclins to CDK4/6 blockade. In contrast, the ability of gain-of-function alterations of D-type cyclins to decrease sensitivity to CDK4/6 inhibitors was less pronounced and restricted to distinct D-type cyclins in a cell line-specific manner. In fact, resistance mechanisms within the D-type cyclin-CDK4/6 axis have been largely attributed to overexpression or amplification of *CDK4* or *CDK6* [38, 67, 68]. While alterations leading to increased D-type cyclin levels have been suggested as potential biomarkers for sensitivity to CDK4/6 inhibitors in cancer cell lines [36], this was not observed for primary breast cancer [69], and data suggest that high levels of cyclin D might also desensitize cells to CDK4/6 blockade by formation of cyclin D-CDK2 complexes which are expected to be insensitive to CDK4/6 inhibitors [40]. Together, our data highlight potentially different routes of resistance to CDK4/6 inhibitors which correspond to the diverging usage of G1 cyclins for cell cycle progression in ATRT.

Our loss-of-function screening approaches in ATRT cells under CDK4/6 inhibition further revealed an unanticipated diverging role of the CRL4^AMBRA1^ ubiquitin ligase for cell cycle progression. AMBRA1 has been described as a WD40 domain protein involved in autophagy and development of the nervous system by acting as a substrate receptor of CRL4 [70–72]. Several studies have revealed extensive CRL crosstalk mediated by AMBRA1 [50, 51], and our data from ATRT cells expand these data by showing substantial interaction of AMBRA1 in particular with substrate receptors of CRL2/5. In addition to its prominent role during autophagy, recent studies highlighted the role of AMBRA1 during cell cycle progression, providing an intriguing explanation for the effects seen after loss of *AMBRA1* during neurodevelopment [40–42]. In contrast to the expected increase in S phase cells upon cyclin D stabilization as a result of *AMBRA1* deficiency, we find that tumor suppressive activity of AMBRA1 in a subset of ATRT cells is directly correlated with deregulation of mitotic factors and a concomitant increase in G2/M phase cells. Of note, negative regulation of mitotic factors by AMBRA1 including aurora kinases was suggested before, but has been disregarded for lack of reproducibility [42].

 We provide several lines of evidence including in silico protein complex modeling as well as immunoprecipitation approaches for a direct interaction of AMBRA1 with several G2/M phase regulators. These include both aurora kinase A and aurora kinase B, and protein levels of both kinases together with their established target phospho-Histone H3 (Ser10) [73, 74], a hallmark feature of mitosis, increase in response to loss of *AMBRA1* in a cell type-specific manner. We show that aurora kinases are regulated in AMBRA1 responder cells by AMBRA1 acting as a substrate receptor for cullin4-RING ligases, rather than its involvement in autophagy-associated processes. Of note, while aurora kinases are known to be degraded by the anaphase-promoting complex/cyclosome (APC/C) [75, 76], inhibition of CRLs in our study using the neddylation inhibitor MLN4924, which is specific for neddylation-dependent cullins not present in the APC/C, provides evidence for the degradation of aurora kinases by CRLs. Surprisingly, however, inhibition of CRLs increased aurora kinases levels in AMBRA1 responder and non-responder cells alike, suggesting that, depending on the cellular context, this regulation is conveyed either by CRL4^AMBRA1^ or another AMBRA1-independent CRL. Together, we identify CRL4^AMBRA1^ as a context-dependent regulator of the stability of aurora kinases, and potentially other G2/M regulators, highlighting a broader role of AMBRA1 and its function as a CRL substrate receptor in the regulation of the cell cycle beyond the destabilization of D-type cyclins.

 While we provide evidence for a direct regulation of aurora kinases by AMBRA1, the mechanism of cell type-specific tumor suppressive activity of AMBRA1 has still to be elucidated. AMBRA1 has been described as a tumor suppressor in diffuse large B-cell lymphoma (DLBCL) [40], lung adenocarcinoma (LUAD) [41, 42], melanoma [43], and these entities may harbor mutations in *AMBRA1*. While it is surprising that ATRT do not seem to harbor AMBRA1 mutations [9, 11], both loss-of-function studies in ATRT cells as well as genetic interaction analyses from DepMap performed in this study strongly support a tumor suppressive role of AMBRA1 in neural crest derivatives including melanoma and rhabdoid tumors. In addition to a potential lineage association, oncogenic KRAS signaling is the best predictor for a tumor suppressive role of AMBRA1 in LUAD [42], and oncogenic BRAF signaling correlates with AMBRA1 tumor suppressive activity in melanoma in DepMap. In fact, all genetic mouse models describing tumor suppressive activity of AMBRA1 so far have been generated on a mutated KRAS/BRAF/MAPK signaling background, and mutations of the KRAS/BRAF/MAPK pathway are common in DLBCL, LUAD, and melanoma [77, 78]. Both oncogenic BRAF and KRAS signaling have recently been shown to act through regulation of kinases involved in mitosis including aurora kinase A and B [79, 80], and based on our findings of an AMBRA1-dependent regulation of mitotic kinases, it seems plausible that tumors with high activity of mitotic regulators might be particularly vulnerable to loss of *AMBRA1*. However, further genetic and biochemical studies will be necessary to decipher the exact molecular mechanism of cell type-specific selectivity of AMBRA1-dependent regulation of mitotic regulators.

 Conversely, KRAS mutated tumors have been shown to present synthetic lethalities particularly for mitotic regulators [81], and we here find that ATRT cells with an oncogenic loss *AMBRA1* are particularly sensitive to AURKA inhibition. Also, while only tested in a small number of ATRT models, oncogenic loss of *AMBRA1* might also predispose to synthetic lethality of CDK4/6 and AURKA inhibition, likely a result of the concerted regulation of these important G1/S and G2/M phase mediators by AMBRA1. However, ATRT cell do not typically present alterations of AMBRA1, and other potential mechanisms of AMBRA1 downregulation need to be determined. Furthermore, the generalizability of this synthetic lethality needs to be further investigated in tumors that frequently present *AMBRA1* alterations.

## Conclusions

In summary, we present a comprehensive and unprecedented resource for further investigations on molecular-based therapeutic strategies for ATRT. Our results particularly warrant further clinical translation of CDK4/6 inhibitors as a promising therapeutic approach for ATRT. Mechanistically, we provide evidence for a previously unappreciated context-dependent role of the ubiquitin ligase receptor AMBRA1 in inhibiting cell cycle progression at the level of mitosis that contributes to an understanding of context-specific differences in tumor suppressive activity.

## Methods

### Experimental design

For genome-wide CRISPR-Cas9 knockout screens, we used ATRT cancer cell lines to identify genetic dependencies following rigorous quality assessment. Together with a set of human cancer cell lines representing other tumor entities (total of 12 human cancer cell lines representing 6 lineages), we performed drug screening to identify most efficacious drugs in ATRT cells. Neurotoxicity of selected drugs was assessed in murine cerebellar granule neurons and human astrocytes. Efficacy of CDK4/6 inhibitors was assessed both in vitro and in vivo using stable cell lines as well as established PDOX models. Correlation of CDK6 and cyclin D2 protein levels was assessed in human primary ATRT tissue by immunohistochemistry. Chemogenetic CRISPR-Cas9 screens were performed both in gain-of-function and loss-of-function mode to identify modulators of response to CDK4/6 inhibition in ATRT cell lines, identifying the ubiquitin ligase receptor AMBRA1 as differential hit in two ATRT cell lines. Identification of substrates of AMBRA1 was performed using in silico complex prediction and co-immunoprecipitations, and validated in biochemical assays.

### Cell lines

The following cell lines were used in this study: CHLA02 (RRID: CVCL_B045), CHLA04 (RRID: CVCL_0F38), CHLA05 (RRID: CVCL_AQ41), CHLA06 (RRID: CVCL_AQ42), CHLA266 (RRID: CVCL_M149), BT12 (RRID: CVCL_M155), BT16 (RRID: CVCL_M156), H1048 (RRID: CVCL_1453), A172 (RRID: CVCL_0131), LN229 (RRID: CVCL_0393), LNZ308 (RRID: CVCL_0394), DAOY (RRID: CVCL_1167), UW228 (RRID: CVCL_0572), Rh30 (RRID: CVCL_0041), HL60 (RRID: CVCL_0002), Jurkat (RRID: CVCL_0367), HT1080 (RRID: CVCL_0317), TC71 (RRID: CVCL_2213), MCF7 (RRID: CVCL_0031), and ATRT-311FHTC [33]. CHLA02, CHLA04, CHLA05, CHLA06, A172, LN229, LNZ308, HT1080, and MCF7 were obtained from ATCC. BT12, CHLA266, Rh30, and TC71 cells were obtained from the Childhood Cancer Repository. BT16 cells were generously provided from Peter Houghton, Greehey Children’s Cancer Research Institute, San Antonio, USA. H1048 cells were generously provided by Roman Thomas, University of Cologne, Germany. DAOY and UW228 cells were generously provided by Ulrich Schüller, University of Hamburg, Germany. HL60 and Jurkat cells were generously provided by Julia Skokowa, University of Tübingen, Germany. ATRT-311FHTC cells were obtained from the Fred Hutch Research Cell Bank, Seattle, USA. CHLA02, CHLA04, CHLA05, and CHLA06 cell were grown in DMEM/F12 media supplemented with B27, EGF, FGF, GlutaMAX, and HEPES. CHLA266, BT12, Rh30, and TC71 cells were grown in IMDM media supplemented with 10% FCS and 1x ITS (Insulin-Transferrin-Selenium). BT16, DAOY, HL60, Jurkat, and HT1048 cells were grown in RPMI media supplemented with 10% FCS. A172, LN229, LNZ308, HT1080, and MCF7 cells were grown in DMEM media supplemented with 10% FCS. All cell line media were supplemented with gentamycin. PDOX-derived ATRT-311FHTC cells were grown in laminin-coated plates in NeuroCult NS-A Basal media supplemented with NS-A Proliferation Supplement, EGF, FGF, Heparin, and penicillin/streptomycin. All cells were authenticated by STR profiling at the Leibniz Institute, DSMZ-German Collection of Microorganisms and Cell Cultures GmbH, Braunschweig, Germany. All cell lines were regularly tested in-house negative for mycoplasma contamination.

### Genetic dependency screens

CRISPR-Cas9 screens using a genome-wide knockout gRNA library were performed as previously described [20]. Briefly, ATRT cell lines were transduced by spinfection with lentiviral particles representing the gRNA library in an all-in-one version containing Cas9 enzyme at a MOI ~0.3. Cell numbers were estimated to ensure a 500x library coverage in the transduced cell population. On the second day, cells were split to three pseudo-replicates, selected by puromycin for 5 days, and kept in culture for a total for 21 days to allow for the depletion of cells where gRNA-guided knockouts affect cell survival or proliferation. For each ATRT cell line individually, optimal transduction and growth conditions were pre-determined. Library coverage was kept at 500x at all sub-culturing steps. After 21 days, genomic DNA was isolated using the QIAamp Blood Maxi kit (Qiagen), and subjected to next-generation sequencing for quantification of gRNA distribution in the remaining cell population. Illumina sequencing was performed as previously described [20].

### Chemogenetic screens

CRISPR screens can be used to functionally investigate gain-of-function or loss-of-function gene alterations and their potential to enhance or suppress the activity of chemical compounds, but also to identify cell cycle regulators in combination with a cytostatically or cytotoxically active drug. These chemogenetic screens were performed in BT16 and CHLA06 cells using genome-wide CRISPR-Cas9 knockout and CRISPR-dCas9-VP64 activation libraries [16, 20]. Cas9 or dCas9-VP64 derivatives were generated by transducing with the lentiviral vector lentiCas9-Blast (Addgene #52962) or lentidCas9-VP64 (Addgene #61425) and selection by blasticidin. Cells were transduced with the CRISPR libraries using spinfection with a predetermined amount of virus to achieve at a MOI ~0.3. Cells were selected 24 h after transduction with puromycin for 5 days, and success of viral transduction and transduction rate was assessed using an inline assay. One week after transduction with CRISPR libraries, cells were split to either drug or DMSO control conditions keeping a minimum 500x library coverage for the respective libraries. Drug screens were treated with abemaciclib or palbociclib at predetermined concentrations to yield a complete cytotoxic effect (for activation screens) or complete cytostatic effect (for knockout screens), in order to prioritize genes conferring resistance or synthetic lethalities, respectively. After 2 weeks of drug or vehicle treatment, genomic DNA was isolated using the QIAamp Blood Maxi, Midi, or Mini kit (Qiagen) depending on the number of surviving cells, and gDNA was subjected to next-generation sequencing.

### Analysis of CRISPR screens

Reads from next-generation sequencing were aligned to the respective CRISPR libraries using PoolQ (v3.6.3). Replicate correlation was performed using gene-level log_2_ fold changes using the top3% most variable genes across all screens, and intra as well as inter cell line Pearson correlation coefficients were calculated. To account for gene-independent effects in knockout screens, we corrected sgRNA-level log_2_ fold changes using *CRISPRcleanR* [82]. To assess quality of knockout screens, we used Cohen’s D [83] and null-normalized median difference (NNMD) as replicate-level and F-measure at bayes factor five [83] as cell line-level statistics based on known essential and non-essential genes [22]. Additionally, we performed precision-recall curve analyses as implemented in the *BAGEL2* algorithm [83]. Finally, depletion of well-known gene sets associated with essential cellular processes was verified by gene set enrichment analyses as implemented in the *CRISPRcleanR* [82] and *MAGeCK-MLE* (v0.5.9.5) [84] packages. Screens for one ATRT cell line (CHLA02) performed poorly according to all our quality metrics, and these screens were excluded from further analyses. To further assess significantly affected genes in dependency screens, either depleted or enriched, we followed two distinct strategies. One, we scored genes using a semi-supervised algorithm (*MAGeCK-RRA*), considering all genes with FDR < 0.1. Second, we scored genes using the supervised *BAGEL2* algorithm, which quantifies the degree of support for each single gene to act like an essential or non-essential gene [83]. The Bayes Factor given by BAGEL2 can be regarded as a combined metric for both statistical significance and effect size. All genes in a given cell line that scored at FDR < 0.1 by both algorithms were considered an essential gene in that cell line. For chemogenetic screens, we averaged across two distinct screens, representing two distinct CDK4/6 inhibitors, for each cell line and compared sgRNA abundance to either the DMSO control for that cell line or the plasmid DNA using the *MAGeCK MLE* algorithm [84]. All genes that scored at FDR < 0.1 in the drug conditions versus the DMSO control, either enriched or depleted, were considered as potential drug modifier. To distinguish between a bona fide drug modifier or a general regulator of cell cycle progression, a nine-square model was used comparing both the drug screens and the DMSO control to the plasmid DNA. Screen results were visualized using the R package *MAGeCKFlute*. *CRISPRcleanR* and *MAGeCKFlute* were run on Rstudio (v4.0.5). *BAGEL2*, *MAGeCK-RRA*, and *MAGeCK-MLE* were run on Python (v3.9.7).

### DNA methylation profiling by 850k EPIC array

Global DNA methylation from seven human ATRT cell lines was assessed using Illumina Infinium MethylationEPIC 850k arrays according to the manufacturer’s instructions. Raw idat files were read and processed using the *minfi* Bioconductor package in R (3.6.0). Briefly, all probes (*n* = 865,859) were subjected to quality control metrics, removing all probes with *P* detection values > 0.01. Furthermore, all probes targeting site on sex chromosomes were removed. Also, all probes with SNPs at the CpG site were removed. In total, 813,439 distinct probes were kept in order to generate *β* values. For prediction of ATRT subgroups, we first generated several models using machine-learning algorithms using a test cohort of 150 primary ATRTs [9]. In order to combine 450k and 850k methylation arrays, we generated virtual arrays using the *minfi* package. Employing the top 2% probes (*n* = 8513) showing highest variability across all three molecular subgroups in the test cohort, we generated prediction models using the *caret* package with the following machine-learning algorithms: random forest (rf), stochastic gradient boosting (gbm), support vector machine (SVM), linear discriminant analysis (lda), k-nearest neighbors (knn), nearest shrunken centroids (PAM), decision tree (dt), and classification and regression trees (cart). The three models that predicted ATRT subgroups with highest accuracy in the test cohort (rf, gbm, svm) were used for further prediction studies. Our random forest model was last evaluated on a validation cohort comprising 121 primary ATRTs [85]. *t*-SNE dimensionality reduction of global DNA methylation data from human ATRT cell lines and a primary CNS tumor reference (*n* = 2801) was performed as previously described [85].

### Custom next-generation onco-panel sequencing

For DNA sequencing, 200 ng of genomic DNA was fragmented to 150–200-bp pairs using ultrasonication on the LE220 Focused-ultrasonicator (Covaris). Library preparation was performed using the SureSelect XT Library Prep Kit (Agilent Technologies) and enrichment of gene of interest was performed using the SureSelect XT Target Enrichment System with custom-designed bait-sets (ssSC v5) covering 708 cancer-related genes, 7 promoter regions and selected fusions. The libraries were sequenced as paired-end 75 bp reads on an Illumina NextSeq500 (Illumina) with a sequencing depth of approximately 25 million clusters per sample. DNA raw data QC and processing was performed using the in-house megSAP Pipeline (https://github.com/imgag/megSAP, version 0.1–1223-gf2879e3) combined with ngs-bits package (https://github.com/imgag/ngs-bits, version 2019_08) [86]. Briefly, sequencing reads were aligned to the human reference genome (GRCh37) by BWA-MEM, variants were called using Strelka2 [87] and annotated with VEP [88]. To obtain high-confidence results, filter criteria for variants were defined as a tumor and normal depth of at least 20x, an allelic frequency of 5% or more and a minimum of 3 reads. NGS onco-panel data were analyzed to retrieve activating mutations of oncogenes as possible driver mutations. Therefore, artefacts, silent mutations (synonymous amino acids), nonsense mutations (gain of stop-codon), and out-of-frame frameshift mutations were rejected. The missense mutations were analyzed using Ensembl Variant Effect Predictor (VEP, https://grch37.ensembl.org/Tools/VEP). Thirty mutations were rated as deleterious in the SWIFT database, as (probably) damaging in the PolyPhen database and/or case reports that these mutations may have clinical impact were available. Sixteen of the detected mutations were rated as oncogenes using the literature based CancerMine database (http://bionlp.bcgsc.ca/cancermine/).

### Gene expression profiling using bulk RNA sequencing

For RNA sequencing, mRNA fraction was enriched using polyA capture from 200 ng of total RNA using the NEBNext Poly(A) mRNA Magnetic Isolation Module (NEB). Next, mRNA libraries were prepared using the NEB Next Ultra II Directional RNA Library Prep Kit for Illumina (NEB) according to the manufacturer’s instructions. The libraries were sequenced as paired-end 50bp reads on an Illumina NovaSeq6000 (Illumina) with a sequencing depth of approximately 25 million clusters per sample. RNA raw data QC and processing was performed using megSAP (version 0.2–135-gd002274) combined with ngs-bits package (version 2019_11-42-gflb98e63). Reads were aligned using STAR v2.7.3a [89] to the GRCh38, and alignment quality was analyzed using ngs-bits. Normalized read counts for all genes were obtained using Subread (v2.0.0) and edgeR (v3.26.6). Further details can be found under https://nf-core.re/rnaseq.

### Copy number analysis

We followed two distinct strategies in this study to assess copy number changes in ATRT cell lines to complement each other. One, for DNA methylation-based analysis of copy-number variation from 850k EPIC arrays, we used the *conumee* package in R (http://bioconductor.org/packages/conumee/). Two, for somatic copy number alteration detection, we used ClinCNV (version 1.16). By analyzing off-target reads, the tool can generate copy-number information about the whole genome, also for targeted panel sequencing data. A gene with an integer copy-number (CN) of ≥ 4 was defined as amplified. A heterozygous deletion was assumed with an integer CN = 1, a homozygous deletion when CN = 0.

### Integration of dependencies, methylation, and gene expression

We used sparse projection to latent structures for the unsupervised integration of ATRT molecular features using the *mixOmics* package (v6.23.4) [90]. As suggested by the package, we reduced the number of molecular features per omics data set by using the top features extending more than 1.5 standard deviations from the mean across all cell lines, resulting in a total of 16,571, 13,116, and 11,404 data points for dependencies (dependency scores, scales log_2_ fold changes), gene expression (normalized read counts, median of ratios method) and promoter methylation (β values). Pairwise sPLS models were generated while keeping 100 features for the first two latent structures for each data set, and correlations of the respective features with the first two latent structures were visualized using correlation circle plots. In order to integrate all three data sets simultaneously, we performed multiblock sPLS as outlined in the package vignette using the top 50 features for the first two latent structures for all omics sets. Multiblock sPLS analysis was visualized using a Circos plot. Direct correlation of either dependency and gene expression or dependency and promoter methylation for all context-specific genes was performed by assessing Pearson correlation coefficients in R. For the null distribution, we randomly permutated the dependency data set and repeated the correlation analysis. Normality of Pearson correlation coefficients was rejected based on a Shapiro-Wilk test for all comparisons, and a Wilcoxon rank sum test was used to test differences of distributions. Based on the results from the above explained sPLS analyses, the alternative hypothesis was set to “two-sided” and “greater” for the RNAseq and methylation analyses, respectively.

### AMBRA1 gene effect associations in DepMap

In order to investigate the effect of AMBRA1 loss-of-function across human cancer cell lines, we interrogated the Broad Dependency Map (DepMap, DepMap Public 24Q2) including data from 1150 human cancer cell lines. Enriched primary diseases based on *AMBRA1* gene effect were identified using a *t*-test and *P* value cutoff of 0.0005, comparing individual primary diseases with the corresponding rest of cancer cell lines. For genetic interactors, we used the top 100 co-dependencies pre-computed by the DepMap for AMBRA1. Genetic interactors were further prioritized for genes that show a skewed *t*-distribution across human cancer cell lines [91], meaning that they harbor distinct gene effects across human cancer cell lines.

### DigiWest analyses

LDS Lysis buffer (Life Technologies, Carlsbad, CA, USA), supplemented with 10% reducing agent (Thermo Fisher Scientific, Waltham, MA, USA), 4% Protease-Inhibitor (Roche Diagnostics GmbH, Mannheim, Germany), and 10% Phosphatase-Inhibitor (Roche) were added to cell pellets on ice. Proteins were denatured by heating to 95°C for 10 min before the lysates were transferred to QiaShredder Eppendorf tubes (Eppendorf, Hamburg, Germany). After centrifugation (16,000*g*, 5 min, RT), eluates were stored at −80°C until further use. Protein quantification was performed using in-gel staining. One microliters of each original lysate was diluted 1:10 (v/v) in lysis buffer. Ten microliters of the respective aliquots was loaded onto a NuPAGE 4–12% Bis-Tris precast gel (Thermo Fisher Scientific) and run according to the manufacturer’s instructions. The gel was washed with water and proteins were stained with BlueBandit (VWR, Darmstadt, Germany) for 1 h. The gel was de-stained overnight with ddH2O before detection at a LI-COR (LI-COR, Bad Homburg, Germany) instrument. Analysis and protein quantification were performed using ImageStudio. DigiWest was performed as published [47]. In brief, 10–12 μg of cellular protein was loaded on an SDS- polyacrylamide gel and size-separated using the commercial NuPAGE system (Life Technologies). Size-separated proteins were blotted onto a PVDF membrane and biotinylated on the membrane using NHS-PEG12-Biotin (50 µM) in PBST for 1 h. After washing with PBST and drying of the membrane, the individual samples lanes were cut into 96strips of 0.5 mm width using an automated cutting plotter (Silhouette America, West Orem, UT, USA) each corresponding to a defined molecular weight fraction. Each of the strips was placed in one well of a 96-well plate and 10 µl elution buffer (8 M urea, 1% Triton-X100 in 100 mM Tris-HCl pH 9.5) was added. The eluted proteins were diluted with 90 μl of dilution buffer (5% BSA in PBS, 0.02% sodium azide, 0.05% Tween-20) and each of the protein fractions was incubated with 1 distinct magnetic color-coded bead population (Luminex, Austin, USA) coated with neutravidin. The biotinylated proteins bind to the neutravidin beads such that each bead color represents proteins of one specific molecular weight fraction. All 96 protein loaded bead populations were mixed resulting in reconstitution of the original lane. Aliquots of the DigiWest bead-mixes (about 1/200th per well) were added to 96-well plates containing 50 µl assay buffer (Blocking Reagent for ELISA; Roche, Rotkreuz, Switzerland) supplemented with 0.2% milk powder, 0.05% Tween-20, and 0.02% sodium azide. Beads were briefly incubated in assay buffer and buffer was discarded. Antibodies were diluted in assay buffer and 30 μl were added per well. After overnight incubation at 15°C, the bead-mixes were washed twice with PBST and species-specific PE-labelled (Phycoerythrin) secondary antibodies (Dianova, Hamburg, Germany) were added and incubated for 1 h at 23°C. Beads were washed twice with PBST prior to readout on a Luminex FlexMAP 3D. For quantification of the antibody-specific signals, an Excel-based analysis tool was employed [47] that automatically identifies peaks of appropriate molecular weight and calculates the peak area (reported as accumulated fluorescence intensity = AFI). Signal intensity was normalized to the total amount of protein loaded onto one lane. The software package MEV 4.9.0 was used for heatmap generation and statistical analysis along with GraphPad Prism (Version 9.0.0). Hierarchical clustering (HCL) was performed using Euclidian Distance and complete linkage. Welch’s *t* test was used for comparisons between groups unless stated otherwise, and *P* < 0.01 was considered significant. For the comparison of control (*n*=12) and *sgAMBRA1* (*n*=12) lines, normalized values were median-centered and log_2_ transformed. For the evaluation of Ambra1 loss in specific cell lines, fold changes of each *sgAMBRA1* sample vs its respective control were computed. Primary antibodies used in this study for DigiWest were the following: β-actin (Sigma, A1978, AMBRA1 (Cell Signaling, 24907), ATM (Cell Signaling, 2873), ATR (Cell Signaling, 2790), AURKA (Cell Signaling, 4718), AURKB (Cell Signaling, 3094), BUB1B (Cell Signaling, 5421), Caspase3 (Cell Signaling, 9662), CDK1 (Cell Signaling, 9112), CDK1-pTyr15 (Cell Signaling, 4539), CDC25A (abm, Y021163), CDC25A-pSer75 (abm, Y011138), CDC25B (R&D, AF1649), CDC27 (Transduction Laboratories, C40920), CDK2 (Cell Signaling, 2546), CDK2-pThr160 (Cell Signaling, 2561), CDK3 (abcam, ab135805), CDK4 (Cell Signaling, 2906), CDK4-pThr172 (Invitrogen, PA-64482), CDK5 (Cell Signaling, 2506), CDK6 (Cell Signaling, 13331), CDK6-pTyr13 (biorbyt, orb15013), CDK6-pTyr24 (biorbyt, orb15014), CHK2 (Cell Signaling, 3440), CHK2-pThr68 (Cell Signaling, 2661), c-MYC (Cell Signaling, 9402), c-MYC-pThr58 (ThermoFisher, PA5-37654), c-MYC-pThr62/Ser62 (abcam, ab32029), cyclin A (abcam, ab53054), cyclin B1 (abcam, ab32053), cyclin D1 (Cell Signaling, 2926), cyclin D1-pThr286 (ThermoFisher, PA5-37487), cyclin D2 (Cell Signaling, 3741), cyclin D3 (Cell Signaling, 2936), cyclin E1 (Cell Signaling, 4129), cyclin E2 (Cell Signaling, 4132), E2F-2 (Millipore, DR1095), E2F-4 (biorbyt, orb10571), histone H3-pSer28 (Millipore, 07–145), hisotne H3-pSer10 (Cell Signaling, 9701), MCM2 (Cell Signaling, 3619), MCM2-pSer139 (Cell Signaling, 8861), MDM2 (Santa Cruz, sc-965), MDM2-pSer166 (Cell Signaling, 3521), p16 (ProteinTech Group, 10883-1-AP), p21 (Cell Signaling, 2947), p27 (Cell Signaling, 3698), p53 (Santa Cruz, sc-126), p53-pSer37 (Cell Signaling, 9289), p53-pSer15 (Cell Signaling, 9284), RB (Cell Signaling, 9313), RB-pSer807/Ser811 (Cell Signaling, 8516), RB-pSer780 (Cell Signaling, 3590), RB-pSer608 (Cell Signaling, 8147), RB-pSer795 (Cell Signaling, 9301), RBPSUH (Cell Signaling, 5313), RPA2 p34 (Millipore, 04–1481), Survivin (Cell Signaling, 2802), TOPK (Cell Signaling, 4942), and TOPO 2 alpha (Santa Cruz, sc-13058).

### Mass spectrometry-coupled immunoprecipitations

In order to overexpress either 3xFLAG tag alone or 3xFLAG-AMBRA1 in ATRT cells, we first generated lentiviral plasmids N174-MCS (Puro) (Addgene #81068) carrying either 3xFLAG or 3xFLAG-AMBRA1. 3xFLAG construct was generated using a DNA oligo hybridization approach, inserting recognition sites for EcoRI and MluI to be used for directed cloning into N174-MCS. 3xFLAG-AMBRA1 open reading frame was PCR amplified from pcDNA3.1-AMBRA1-3xFLAG using adequate primers to be used for directed cloning into N174-MCS using EcoRI/MluI sites. Lentiviral particles were generated and expression was verified by western blot.

Cells expressing 3xFLAG or 3xFLAG-AMBRA were lysed and processed for affinity purification using anti-FLAG beads. For immunoprecipitations followed by mass spectrometry, experiments were performed with and without inhibition of CRLs by MLN4924 to potentially discriminate between interactors of AMBRA1 and substrates which are proteolytically degraded upon AMBRA1 binding. Briefly, a similar amount of total cellular lysates was loaded on slurry mixed anti-Flag M2 beads (SIGMA). Following overnight incubation at 4C in an end-over-end shaker, the supernatants were discarded, beads were washed twice with Tris Buffer (pH 7.4), and proteins were eluted from beads using on-beads enzymatic digestion. Trypsin and LysC (Thermo) were used to elute peptides from the Flag beads. Peptides were desalted and purified using StageTips (Thermo) and stored at −80 °C prior to LC-MS analysis.

Mass spectrometry analysis was performed as described [92] in Data Independent Analysis (DIA) mode. Briefly, MS was performed on an Ultimate3000 RSLC system coupled to an Orbitrap Fusion Tribrid mass spectrometer (Thermo Fisher Scientific). Digested peptides were loaded onto a µPAC Trapping Column at 10 µl per min flow rate in 0.1% trifluoroacetic acid in HPLC-grade water. Peptides were eluted and separated on the PharmaFluidics 50 cm µPAC C18 nano-LC column by a linear gradient from 2 to 30 % of buffer B (80% acetonitrile and 0.08% formic acid in HPLC-grade water) in buffer A (2% acetonitrile and 0.1% formic acid in HPLC-grade water) at a flow rate of 300 nl per min. The remaining peptides were eluted by a short gradient from 30 to 95% buffer B; the total gradient run was 120 min. Spectra were acquired in DIA mode using 30 variable-width windows (isolation windows: 66, 31, 27, 19, 19, 18, 16, 19, 14, 18, 19, 17, 17, 17, 20, 21, 21, 24, 26, 21, 28, 31, 33, 32, 39, 45, 49, 63, 99, and 289) over the mass range 350–1500 m/z. MS2 scan range was set from 200 to 2000 m/z, with an AGC target of 5E4 and a maximum injection time of 54 ms.

MS RAW data were analyzed using DIA-NN 1.8.1 (https://github.com/vdemichev/DiaNN) in library-free mode against the human database (UniProt release October 2022). Only high-accuracy spectra with a minimum precursor FDR of 0.01, and only tryptic peptides were used for protein quantification. The match between runs option was activated and no shared spectra were used for protein identification. Statistical analysis including label-free quantification ratios (LFQ), and one-sided corrected permutation-based *T*-test (250 permutations and a minimum *p*-value of 0.05) to identify putative interactors of AMBRA1 in each cell line, were done using the Perseus software suite version 1.6.15.0.

### Ni-NTA pull-down

Cell lines were transfected with pCMV (Clontech) vectors carrying either a 6xHis tag alone (control) or 6xHis-tagged ubiquitin 48 h prior to the experiment using Lipofectamine 3000. Cells were collected after 6 h treatment with bortezomib (1 μM) in lysis buffer (10 mM Tris pH 8.0, 100 mM NaH_2_PO_4_, and 8 M urea) containing 10 mM imidazole. Total protein (600 μg) was incubated with Ni-NTA agarose beads (Qiagen) overnight at 4 °C on shaker. Agarose beads were washed in lysis buffer (pH 6.3) containing 20 mM imidazole four times. Proteins were eluted in 2x Laemmli buffer at 95 °C, and input as well as pull-down was subjected to western blot analysis. For quantification, target proteins in the input samples were normalized to levels of β-tubulin to account for differences in total protein loaded. Protein signals in pull-down samples were normalized to the total amount of ubiquitylated proteins per sample as assessed by probing with an His-specific antibody. Total ubiquitylation of AURKA or AURKB was defined as the normalized target signal in pull-downs relative to the normalized target signal in the corresponding input.

### Protein complex prediction using ColabFold

To analyze heteromeric protein complexes, we used the amino acid sequence of the AMBRA1^WD40^ domain together with full-length sequences of selected G2/M phase regulators. Complexes were modelled using the ColabFold (v1.5.2) available at github (https://github.com/sokrypton/ColabFold). We used ColabFold that uses the AlphaFold2 algorithm [48] together with MMseqs2 for sequence alignments. Alphafold_multimer_v3 was used as model type for complex prediction with 20 recycling iterations. The corresponding protein data bank file from the top-ranking model was used as input for ChimeraX to generate protein cartoon structures. Corresponding .json file were used to visualize inter-chain alphafold contacts below eight angstroms. Plots illustrating predicted alignment errors for all pairwise residue comparisons in heteromeric complexes and predicted template modeling scores were generated using a custom jupyter notebook.

### Archived tissue samples from ATRT patients

For immunohistochemistry studies, we used archival tissue from 17 ATRT patients (3 females, 14 males), average age was 1.9 years ± 2.5. Molecular subgroups of ATRT tumors had been determined by global DNA methylation profiles within the clinical routine diagnostics using the methylation classifier for central nervous system tumors (www.molecularneuropathology.org).

### Orthotopic xenograft mouse models

For orthotopic implantation of tumor cells, Crl:CD1-Foxn1^Nu/Nu^ nude mice were used (Charles River). All mice were female, and between 8 and 10 weeks old at the time of tumor cell implantation. All mice were kept at a 12-h light/dark cycle, with a maximum of four animals per cage. Animal husbandry including supply of food, water, and litter was performed by professional animal care takers. Sample size calculation for experimental groups was performed by a biometrician. For both orthotopic xenograft mouse models (BT16 and ATRT310FH), we injected 200,000 cells into the forebrain (2 mm lateral and 1 mm anterior to bregma). Subsequently, mice were randomly assigned to experimental groups. Abemaciclib treatment was scheduled daily at a concentration of 75 mg/kg by oral gavage. Vehicle animals were treated with the same volume of isotonic NaCl solution. Treatments were performed for 3 weeks, with 2-day drug holidays on weekends.

### Customized drug library for pharmacological validation

Based on all genetic dependencies identified in any of the ATRT cell lines, we used the Drug Gene Interaction database (DGIdb) to identify potential chemical vulnerabilities [27]. DGIdb consolidates and organizes the druggable genome in a tissue/cell type agnostic manner in two classes: genes with known drug interactions, as well as genes that are in potentially druggable categories. All drug gene interactions with a potential small molecule inhibitor or antagonist were considered. The final library included 37 small molecule inhibitors targeting ATRT genetic dependencies, five positive controls based on previous work (dasatinib, nilotinib, dorsomorphin, DAPT, ML329) [9, 11], as well as two broadly cytotoxic agents (vincristine, doxorubicin).

### Cell culture and growth rate inhibition assays

Cells were cultured in the abovementioned media under empirically optimized conditions. All cells were cultured in 100 µl supplemented with the corresponding compound at three distinct concentrations (10 nM, 100 nM, 1000 nM) in 96-well plates. Each plate contained a DMSO control that was used to normalize all data from this plate. Each condition on each plate contained 8 replicate values that were averaged upon analysis. We performed the drug screen in two sessions, and tested the coherence of both sessions constructing a similarity index [93]. Cell viability was assessed using CellTiter-Blue (Promega).

### Neurotoxicity assays

For assessment of neurotoxic activity of selected drugs from the ATRT compound library, we used postmitotic cerebellar granule neurons as well as human astrocytes as surrogates for normal cell types of the brain. Granule neurons were generated from wildtype pups at P5. Cerebella were dissected under the microscope, meninges removed, and cerebella were triturated in trypsin/EDTA containing DNase. After washing, cells were counted, and seeded in DMEM/F12 media supplemented with 10% FCS, 25 mM KCL, 1x GlutaMAX, and Pen/Strep onto polyornithine-coated 96-well plates. On the second day, 10 µM Ara-C were added for 4 days in order to enrich for postmitotic granule neuron cells. In order to test our compounds in a glia-representing lineage, we used normal human astrocytes (NHA) cells from Lonza which were cultured according to the manufacturer’s instructions.

### Genetic validation experiments

For knockdown experiments of *CDK4*, *CDK6*, *CCND1*, *CCND2*, and *CCND3*, two distinct shRNA for each target gene were cloned into pLKO.1 puro (Addgene #8453) according to the corresponding protocol. shRNA sequences were as follows: *shCDK4* #1, 5’-TTTATCTCTGAGGCTATGGACCTCGAGGTCCATAGCCTCAGAGATAAA-3’, *shCDK4* #2, 5’-CTTTATCTCTGAGGCTATGGACTCGAGTCCATAGCCTCAGAGATAAAG-3’, *shCDK6* #1, 5’-CAGATGTTGATCAACTAGGAACTCGAGTTCCTAGTTGATCAACATCTG-3’, *shCDK6* #2, 5’-CCAGAACACCTCGGAGCTGAACTCGAGTTCAGCTCCGAGGTGTTCTGG-3’, *shCCND1* #1, 5’-ATTGGAATAGCTTCTGGAATCTCGAGATTCCAGAAGCTATTCCAATC-3’, *shCCND1* #2, 5’-CCACAGATGTGAAGTTCATTTCTCGAGAAATGAACTTCACATCTGTGG-3’, *shCCND2* #1, 5’-GAAGGACATCCAACCCTACATCTCGAGATGTAGGGTTGGATGTCCTTC-3’, *shCCND2* #2, 5’-AGGAACTGTGTACGCCATTTACTCGAGTAAATGGCGTACACAGTTCCT-3’, *shCCND3* #1, 5’-CGCTGTGAGGAGGAAGTCTTCCTCGAGGAAGACTTCCTCCTCACAGCG-3’, *shCCND3* #2, 5’-CCAGCACTCCTACAGATGTCACTCGAGTGACATCTGTAGGAGTGCTGG-3’, *shRPL14*, 5’-GCGATTGTAGATGTTATTGATCTCGAGATCAATAACATCTACAATCGC-3’, *shLuc*, 5’-CGTGATCTTCACCGACAAGATCTCGAGATCTTGTCGGTGAAGATCACG-3’. Lentiviral particles were produced in HEK293FT cells, and ATRT cell lines were subsequently transduced with viral supernatants using spinfection. Forty eight hours after transduction, transduced cells were selected using puromycin for 5 days, and subsequently cultured in triplicates in 96-well plates. Cell viability was assessed at several time points after seeding using CellTiter-Blue (Promega). Cell viability was compared as percent viability of a corresponding condition using a shRNA directed against the luciferase gene.

Knockout experiments for *AMBRA1* were performed using lentiviral particles based on lentiCRISPRv2_*AMBRA1*#1 (Addgene #174152). For overexpression studies, full length open reading frames were PCR amplified for *CCND1* (Addgene #172632), *CCND2* (Addgene #172629), *CCND3* (Addgene #172623), *CCNE1* (Addgene #164144), and *CCNE2* (Addgene #19935) using custom-made primers including restriction enzymes recognition sites for EcoRI and MluI for cloning into N174-MCS (Puro) (Addgene #81068). The open reading frame for *AURKA* (Addgene #23532) was PCR amplified for EcoRI/MluI directed cloning into N174-MCS (Addgene #81061). Lentiviral particles were produced in HEK293FT cells, and cells were transduced by spinfection. After transduction, cells were selected using either puromycin (N174-MCS (Puro)), or G418 (N174-MCS) under empirically determined concentrations. For overexpression of AURKA in AMBRA-depleted cells, ATRT cells were first transduced with lentiCRISPRv2_*AMBRA1*#1 lentiviral particles, selected with puromycin, and subsequently transduced with N174-*AURKA* lentiviral particles and selected with G418.

### Cell cycle analyses

Briefly, cells were collected and isolated using accutase and resuspended in PBS buffer with 1 g/L glucose. After spinning, cells were fixed by ice-cold ethanol. After washing, cells were rehydrated in buffer and stained with propidium iodide (50 µg/ml in 0.2% Triton X-100, RNase in buffer). After 15 min of staining, cells were subjected to measurement on a MACSQuant machine. Data were analyzed using FlowJo.

### Western blotting

Whole cell lysates were prepared using either RIPA (25 mM Tris-HCl pH 7.6, 150 mM NaCl, 1% NP-40, 1% sodium deoxycholate, 0.1% SDS) or urea buffer (10 mM Tris HCl pH 8.0, 100 mM NaH_2_PO_4_, 8 M urea). Protein gels were run using 10% BOLT Bis-Tris or 4–12% NuPAGE Bis-Tris polyacrylamide precast gels. Proteins were blotted onto PVDF or nitrocellulose membranes and detected using indicated antibodies as per standard methods. Visualization was done using HRP-coupled, species-specific secondary antibodies. Primary antibodies used in this study for western blotting were the following: CDK4 (abcam, ab199728), CDK6 (Cell Signaling, 13331), cyclin D1 (Cell Signaling, 55506), cyclin D2 (Cell Signaling, 3741), cyclin D3 (Cell Signaling, 2936), cyclin E1 (Cell Signaling, 20808), cyclin E2 (Cell Signaling, 4132), GAPDH (Cell Signaling 2118), Vinculin (Cell Signaling, 13901), β-tubulin (Cell Signaling, 86298), AMBRA1 (Cell Signaling, 24907), AURKA (Cell Signaling, 14475), AURKB (Cell Signaling, 3094), CDK1 (Cell Signaling, 77055), LC3B (Cell Signaling, 83506), PDGFR β (Cell Signaling, 3169), DDB1 (Cell Signaling, 6998), CUL4A (Cell Signaling, 2699), FLAG (Sigma-Aldrich, F7425), His (addgene, 184180).

### Immunohistochemical analyses

To assess CDK6 and cyclin D2 expression in samples from ATRT patients via immunohistochemistry, we used archived formalin-fixed, paraffin-embedded (FFPE) tumor material. FFPE tissue specimens were cut to 2.5-µm-thick sections using a microtome, mounted on glass slides and subjected to immunhistochemical (IHC) staining. The stainings were performed using either CDK6 monoclonal rabbit anti-human antibody (Clone EPR4515, dilution 1:250, Abcam, Cambridge, UK) or Cyclin D2 monoclonal rabbit anti-human antibody (Clone D52F9, dilution 1:50, Cell Signaling, Cambridge, UK). The IHC procedure was conducted using an automated immunostainer (BenchMark ULTRA IHC/ISH Staining Module, Hoffmann-La Roche, Basel, CH) together with the respective reagents (as listed below) and according to the protocols provided by the manufacturer. For detection, the OptiView DAB IHC protocol was used including the following steps: Deparaffinization for 4 min at 72 °C, washing with EZ Prep, incubation with Cell Conditioner No.1 for 64 min at 100 °C, incubation with OV PEROX IHBTR for 4 min, incubation with primary antibody for 32 min at 37 °C, and incubation in sequence with OV HQ UNIV LINKR, then incubation with OV HRP MULTIMER and then incubation with OV DAB and OV H2O2 for 8 min each, last incubation with OV Copper for 4 min. The counterstaining was performed with hematoxylin for 20 min upon which the tissues were incubated with BLUING REAGENT for 8 min prior to mounting the coverslips. Multiple intervening washing steps were included. All images were derived from tissue areas with evident tumor pathology, corresponding to approximately 1500 cells per sample and obtained using bright-field microscopy with a ×40 objective (Olympus BX61). After adjusting a common threshold in all images to highlight stained cells, immunoreactivity was measured on ImageJ software as percentage area of CDK6 or cyclin D2-positive cells.

### Time-lapse imaging

M phase duration was determined by live-cell phase contrast imaging using a Leica DMi8 microscope with a ×20 objective. Briefly, cells were seeded on Lab-Tek II 8 chamber coverglass systems, treated with LY329568 at 200 nM, and imaged every 20 min for 60 h. Time in M phase was measured as the time between cell round up/chromosome condensation and completion of cytogenesis. Quantification was done by tracking 50 cells per cell line and condition in ImageJ.

### Statistical analysis

No statistical methods were used to predetermine sample sizes except for in vivo studies, but the sample sizes here are similar to those reported in previous publications. Differences were considered statistically significant at *P* < 0.05 if not stated otherwise, and sample sizes are indicated in each figure legend. For the analysis of CRISPR-Cas9 dependency screens, two distinct statistical approaches were performed. One, we used a Bayesian classifier implementing a cross-validation strategy to determine the log likelihood for either of two models, being the distributions of sgRNAs targeting known essential and non-essential genes. Two, we performed a ranking approach based on a negative binomial model and performed robust rank aggregation in order to identify negatively or positively selected genes. For chemogenetic screens modeling differences for several drug screens versus a common reference, we used a maximum likelihood estimation approach. For all correlation studies, a line derived from nonlinear regression was fitted onto the data, and correlation was computed using Pearson’s *r*. For all correlations, Pearson’s *r*, R squared, and *P* values are shown. To compare the distribution of *z* scores relating to drug screen data, we used a Wilcoxon rank sum test and calculated the corresponding effect size. Intersection analyses for genetic dependencies were calculated using the *SuperExactTest* package [29]. All growth rate inhibition assays were analyzed using the *GRmetrics* package [28]. For survival analyses, we used the Log-rank test, and survival was defined as the time period between surgery and the onset of neurological symptoms, or the presence of any other exclusion criteria defined by local authorities. Differential gene expression analyses from RNA-seq data was performed using *DESeq2* [94], using a cutoff of log_2_ fold change > 1 and adjusted *P* value < 0.01 for significance. For comparisons of more than 2 groups across more than 2 variables, we used two-way ANOVA with correction for multiple testing as indicated in the corresponding figure legends. All statistical analyses were performed in GraphPad Prism 9 or R (v4.0.5). Additional information on statistical approaches can be found in the above subsections "Analysis of CRISPR screens", "Integration of dependencies, methylation, and gene expression", "DigiWest analyses", and "Mass spectrometry-coupled immunoprecipitations".

## Supplementary Information

Additional file 1: Figure S1. A detailed molecular classification of human ATRT cell lines. Figure S2. Quality control for CRISPR-Cas9 knockout screens in human ATRT cell lines. Figure S3. DepMap comparison and functional annotation of ATRT-context essential genes. Figure S4. Extended analyses of molecular predictors of gene essentiality in ATRT cell lines. Figure S5. ATRT drug screen quality control and neurotoxicity validation of top drug candidates. Figure S6 Extended interaction analyses of genetic/chemical vulnerabilities and molecular subgroups of ATRT. Figure S7. Analyses for CDK4/6 inhibitor sensitivity in ATRT cells. Figure S8. Gain-of-function CRISPR drug screens and transcriptional analysis of CDK4/6 blockade effects in ATRT cells. Figure S9: Loss-of-function CRISPR drug screens and validation of AMBRA1 as screen hit. Figure S10: AMBRA1 gene effect associations from DepMap and mutation frequency. Figure S11: Changes in protein abundance upon loss of AMBRA1 in ATRT cells. Figure S12: Interactome analysis for AMBRA1 in ATRT cells.

Additional file 2: Table S1. Summary of oncopanel results and copy number variation (CNV) analyses for ATRT cell lines.

Additional file 3: Table S2. Summary of BAGEL2/MAGeCK RRA analyses for CRISPR-Cas9 dropout screens in ATRT cells.

Additional file 4: Table S3. Drug-gene interactions and potential druggability from the Drug Gene Interaction Database for genetic dependencies in ATRT cells.

Additional file 5: Table S4. Cell line overview and growth rate inhibition data from three-dose drug screen.

Additional file 6: Table S5. Summary of MAGeCK MLE results for activation chemogenetic screens using CDK4/6 inhibitors in ATRT cells.

Additional file 7: Table S6. Summary of MAGeCK MLE results for knockout chemogenetic screens using CDK4/6 inhibitors in ATRT cells.

Additional file 8: Table S7. DigiWest analyses of changes in protein levels upon loss of AMBRA1 in ATRT cells.

Additional file 9: Table S8. Mass spectrometry analyses for FLAG-AMBRA1 co-immunoprecipitations in human ATRT cell lines.

Additional file 10: Movie S1. Time-lapse movie from BT12 sgAMBRA1 cells treated with DMSO.

Additional file 11: Movie S2. Time-lapse movie from BT12 sgAMBRA1 cells treated with 200 nM LY3295668.

Additional file 12: Movie S3. Time-lapse movie from CHLA06 sgAMBRA1 cells treated with DMSO.

Additional file 13: Movie S4. Time-lapse movie from CHLA06 sgAMBRA1 cells treated with 200 nM LY3295668.

Additional file 14: Uncropped western blots for Figure S1D.

Additional file 15: Uncropped western blots for Figure 3D.

Additional file 16: Uncropped western blots for Figure S8C.

Additional file 17: Uncropped western blots for Figure 6B.

Additional file 18: Uncropped western blots for Figure 7C-E.

Additional file 19: Review history.

## Data Availability

All major code from this study, particularly code used to analyze CRISPR screens, can be found at the corresponding Zenodo Github page (10.5281/zenodo.10137598) [95]. All omics data generated in this study can be found under GEO SuperSeries GSE231287 [96], or can be accessed at figshare for dependency screens (10.6084/m9.figshare.23552070.v1) [97] and chemogenetic screens (10.6084/m9.figshare.23552100.v1) [98]. Mass spectrometry data can be accessed via the Proteomics IDEntifications Database under identifier PXD043369 (https://www.ebi.ac.uk/pride/archive/projects/PXD043369) [99].
