# Supplementary material for: Functional screening reveals genetic dependencies and diverging cell cycle control in atypical teratoid rhabdoid tumors
Source: Genome Biol. 2024 Dec 2;25:301. doi: 10.1186/s13059-024-03438-w (PMC11610224; doi:10.1186/s13059-024-03438-w)
Supplement: Supplementary file 1 — Additional file 1: Figure S1. A detailed molecular classification of human ATRT cell lines. Figure S2. Quality control for CRISPR-Cas9 knockout screens in human ATRT cell lines. Figure S3. DepMap comparison and functional annotation of ATRT-context essential genes. Figure S4. Extended analyses of molecular predictors of gene essentiality in ATRT cell lines. Figure S5. ATRT drug screen quality control and neurotoxicity validation of top drug candidates. Figure S6 Extended interaction analyses of genetic/chemical vulnerabilities and molecular subgroups of ATRT. Figure S7. Analyses for CDK4/6 inhibitor sensitivity in ATRT cells. Figure S8. Gain-of-function CRISPR drug screens and transcriptional analysis of CDK4/6 blockade effects in ATRT cells. Figure S9: Loss-of-function CRISPR drug screens and validation of AMBRA1 as screen hit. Figure S10: AMBRA1 gene effect associations from DepMap and mutation frequency. Figure S11: Changes in protein abundance upon loss of AMBRA1 in ATRT cells. Figure S12: Interactome analysis for AMBRA1 in ATRT cells. [file 13059_2024_3438_MOESM1_ESM.pdf]

Functional screening reveals genetic dependencies and diverging cell cycle control in atypical teratoid rhabdoid tumors

Daniel J. Merk, Foteini Tsiami, Sophie Hirsch, Bianca Walter, Lara A. Haeusser, Jens D. Maile, Aaron Stahl, Mohamed A. Jarboui, Anna Lechado-Terradas, Franziska Klose, Sepideh Babaei, Jakob Admard, Nicolas Casadei, Cristiana Roggia, Michael Spohn, Jens Schittenhelm, Stephan Singer, Ulrich Schüller, Federica Piccioni, Nicole S. Persky, Manfred Claassen, Marcos Tatagiba, Philipp J. Kahle, David E. Root, Markus Templin, and Ghazaleh Tabatabai

Supplementary figures

Figure S1. A detailed molecular classification of human ATRT cell lines.

Figure S2: Quality control for CRISPR-Cas9 knockout screens in human ATRT cell lines.

Figure S3: DepMap comparison and functional annotation of ATRT-context essential genes.

Figure S4: Extended analyses of molecular predictors of gene essentiality in ATRT cell lines.

Figure S5: ATRT drug screen quality control and neurotoxicity validation of top drug candidates.

Figure S6: Extended interaction analyses of genetic/chemical vulnerabilities and molecular subgroups of ATRT.

Figure S7: Analyses for CDK4/6 inhibitor sensitivity in ATRT cells.

Figure S8: Gain-of-function CRISPR drug screens and transcriptional analysis of CDK4/6 blockade effects in ATRT cells.

Figure S9: Loss-of-function CRISPR drug screens and validation of AMBRA1 as screen hit.

Figure S10: AMBRA1 gene effect associations from DepMap.

Figure S11: Changes in protein abundance upon loss of AMBRA1 in ATRT cells.

Figure S12: Interactome analysis for AMBRA1 in ATRT cells.

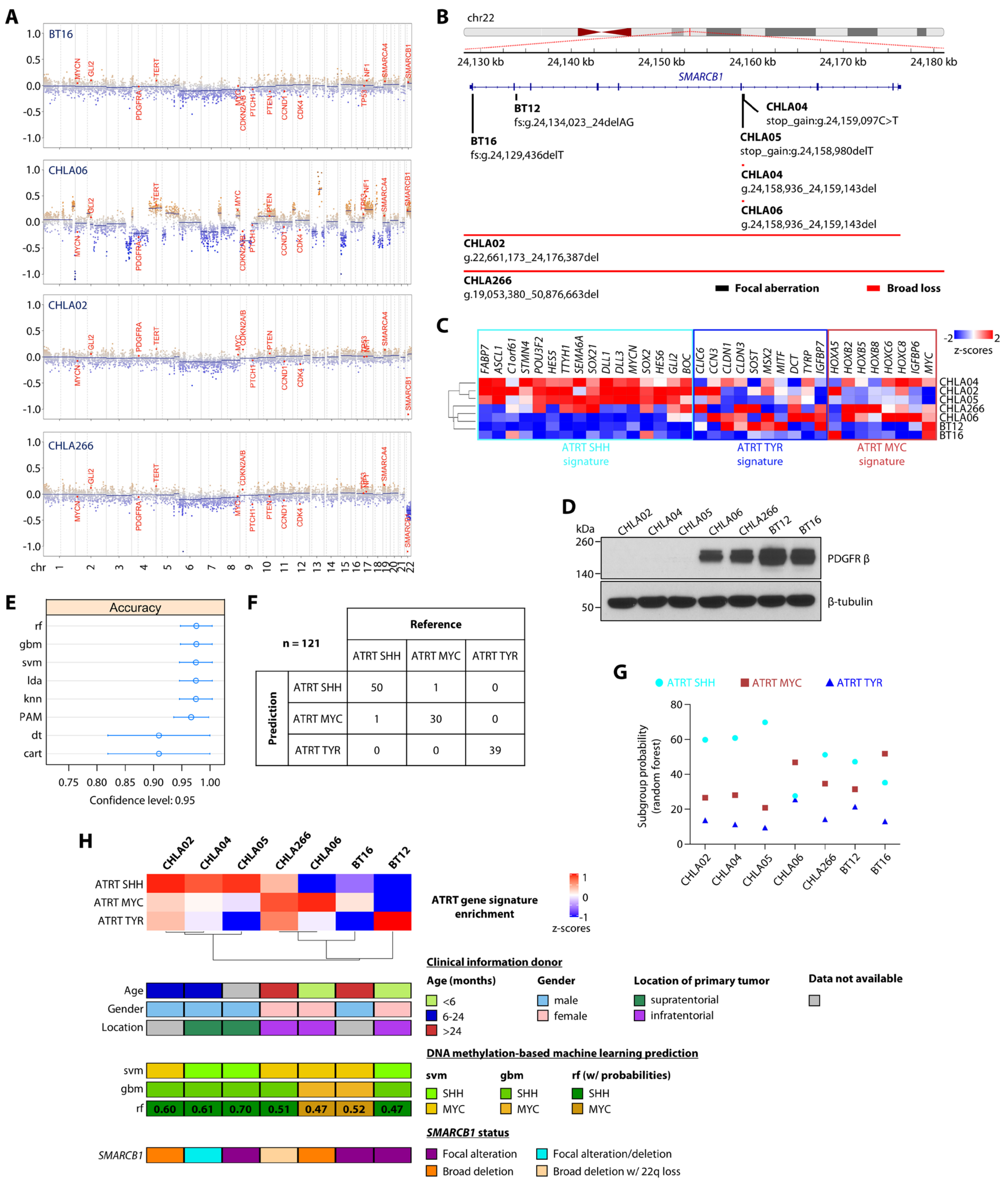

n = 121		Reference		
		ATRT SHH	ATRT MYC	ATRT TYR
Prediction	ATRT SHH	50	1	0
	ATRT MYC	1	30	0
	ATRT TYR	0	0	39

Figure S1. A detailed molecular classification of human ATRT cell lines. A Representative copy number variation plots for selected ATRT cell lines. Selected brain tumor-associated genes are highlighted. **B** Overview of *SMARCB1* alterations in ATRT cell lines. **C** Heat map illustrating the enrichment of ATRT subgroup-associated gene signatures in ATRT cell lines. **D** Western blot analysis of protein level of PDGFR β in ATRT cell lines. **E** Accuracy of several machine learning algorithms in predicting ATRT subgroups from global DNA methylation profiles in the validation cohort. **F** Confusion matrix illustrating accuracy of the random forest model in predicting ATRT subgroups in the test cohort. **G** Subgroup probabilities for human ATRT cell lines from DNA methylation as predicted by the random forest model. **H** Overview of key molecular and clinical parameters of ATRT cell lines including prediction of subgroup affiliation based on gene expression and global DNA methylation.

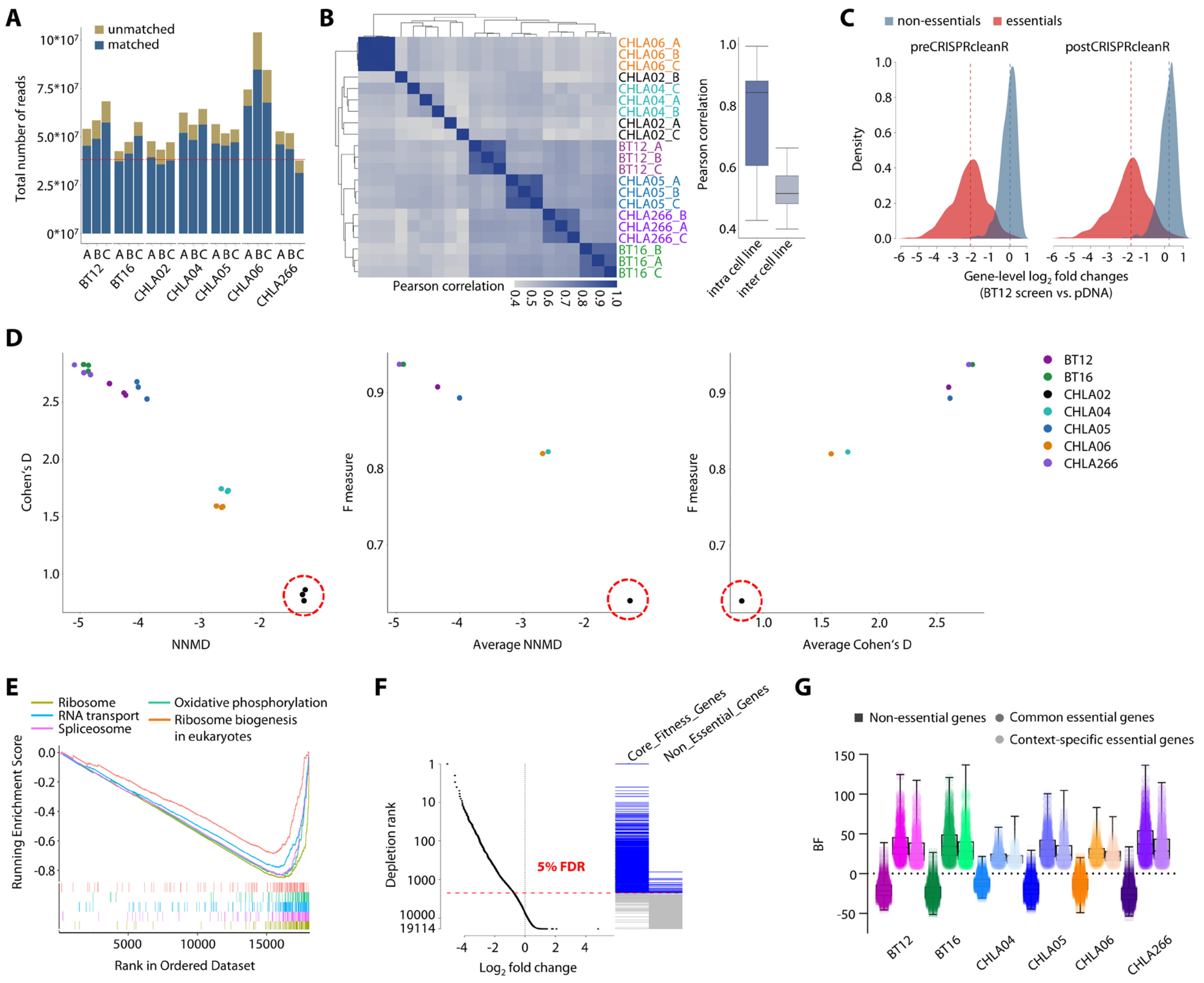

Figure S2. Quality control for CRISPR-Cas9 knockout screens in human ATRT cell lines. A Number of unmatched and library-matched read counts from a total of 21 CRISPR-Cas9 knockout screens. Red line illustrated number of reads providing a 500x library coverage. **B** Left, pairwise correlations (Pearson's *r*) for all technical replicates from ATRT screens based on normalized gRNA read counts. Right, distribution of intra and inter cell line correlation coefficients for all screens via a box-plot (center line is median, box limits are upper and lower quartiles, whiskers are 1.5x interquartile range). **C** Distribution of gene-level $\log_2$ fold changes for known non-essential and essential genes in BT12 cells before and after correction for gene-independent effects using CRISPRcleanR. **D** Correlation of three screen quality metrics. For replicate level quality assurance, Cohen's D and null-normalized median difference (NNMD) were employed. For quality assessment on cell line level, the harmonic mean of precision and recall (F measure) at bayes factor 5 was calculated. The uniformly poor performing cell line CHLA02 is encircled in red. **E** Gene set enrichment analyses for six ATRT cell lines (CHLA04, CHLA05, CHLA06, CHLA266, BT12, BT16) illustrating coherent depletion of pan-essential gene sets across all screens as determined by MAGeCK-MLE. **F** Average gene-level depletion profile for six ATRT screens (CHLA04, CHLA05, CHLA06, CHLA266, BT12, BT16) with superimposed core fitness and non-essential gene sets. Recall at 5% FDR is shown in red. **G** Distribution of bayes factors with respect to non-essential, common essential and context-specific essential genes in individual ATRT cell lines.

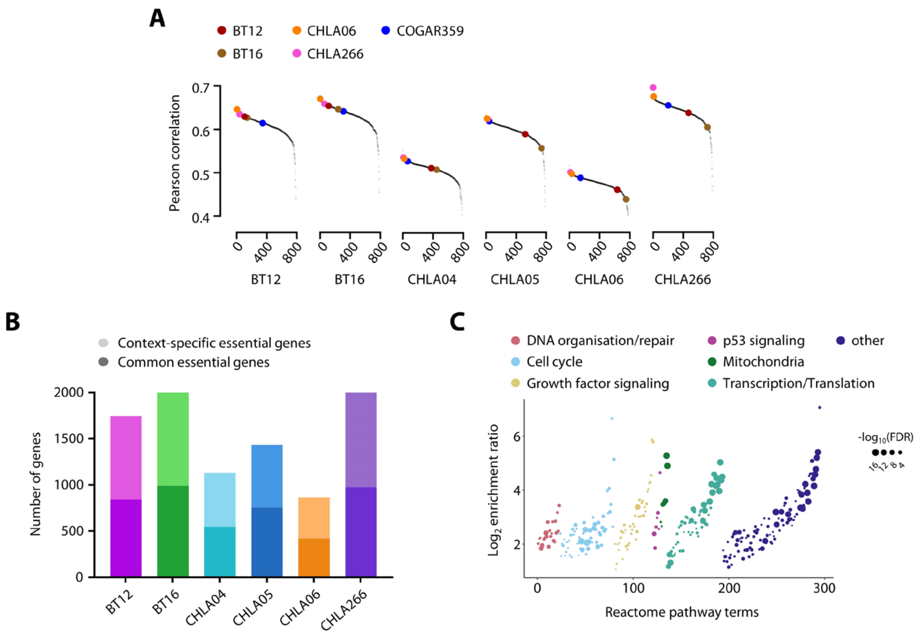

Figure S3. DepMap comparison and functional annotation of ATRT-context essential genes. A Similarity of depletion profiles for ATRT cell lines screened with the Brunello library in this study (x axis) to cell lines from DepMap screened with the Avana library. All ATRT cell lines within DepMap are shown (colored circles). **B** Bar plot showing number of common essential and context-specific essential genes for six ATRT cell lines fulfilling FDR < 10% for both BAGEL2 and MAGeCK-RRA (neg-FDR) analyses. **C** Illustration of top enriched gene ontologies in context-specific essential genes interrogating the Reactome pathway database. Statistics are derived from a hypergeometric test (C).

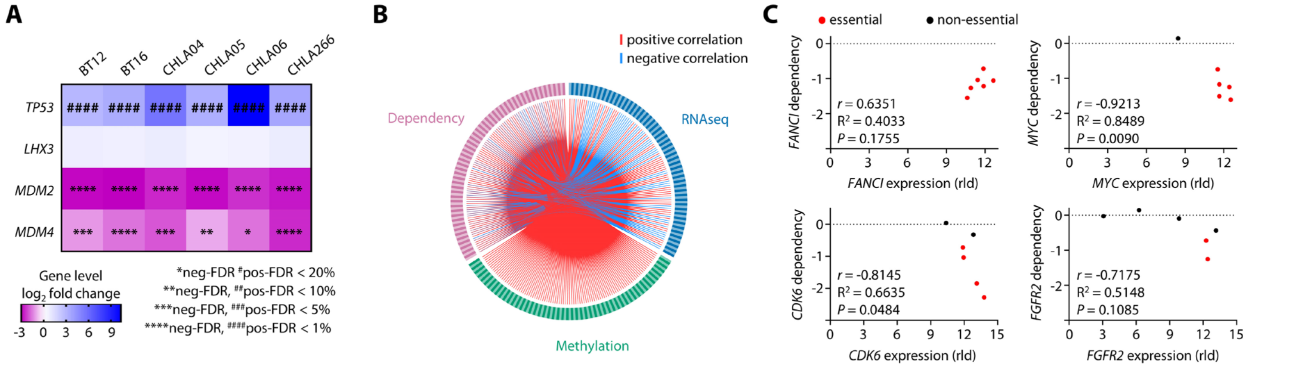

Figure S4. Extended analyses of molecular predictors of gene essentiality in ATRT cell lines. A Heat map illustrating the enrichment of TP53 and the depletion of negative regulators of p53 signaling MDM2 and MDM4 in CRISPR-Cas9 knockout screens of ATRT cell lines. **B** Circos plot illustrating results from a multiblock sPLS analysis integrating gene expression and gene promoter methylation with gene essentiality. The lines within the circle represent positive or negative correlations (all > 0.9) for the top 50 variables on the first two components. **C** Scatter plots illustrating the correlation of gene dependency scores and expression for selected genes known to be context-specific essentials across cancer cell lines according to DepMap (FANCI) or genes with distinct essentiality profiles in our study of ATRT cell lines (MYC, CDK6, FGFR2). Statistics are derived from robust rank aggregation (A), or t tests (C).

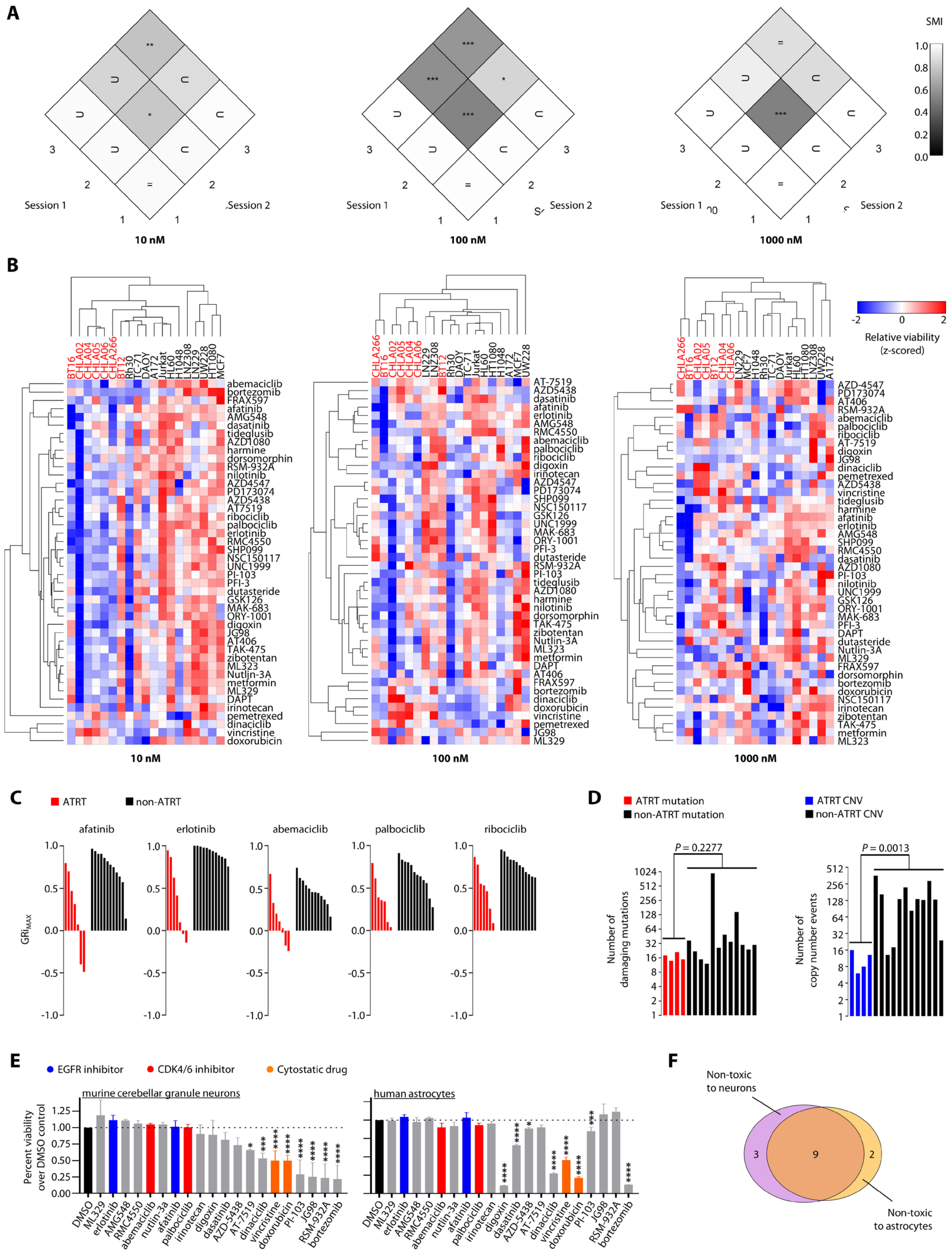

Figure S5. ATRT drug screen quality control and neurotoxicity validation of top drug candidates. A Diamond plots showing the similarity matrices index (SMI) for the comparison of session 1 and session 2 of the three-dose drug screen based on the effects on cell viability relative to DMSO control. Orthogonal projection has been used to compare the first three subspaces of each matrix. **B** Heat maps illustrating the z-scored effect of the ATRT drug library on cell viability as compared to DMSO control (1 minus Pearson correlation, average linkage). ATRT cell

lines are indicated in red. **C** Bar graphs depicting GRi_{MAX} values for EGFR and CDK4/6 inhibitors in ATRT and non-ATRT reference cell lines as determined by the drug screen shown in Fig. 2. **D** Bar graphs illustrating the number of damaging mutations (left) and the number of gene copy number events (right) for ATRT and non-ATRT cells. Available data from the Cancer Cell Line Encyclopedia included four ATRT and 12 non-ATRT cell lines. **E** Neurotoxicity testing of most promising small molecule candidates (GRmax < 0.5 in at least four ATRT cell lines, n=20) on postmitotic murine cerebellar granule neurons and human astrocytes. Cells were treated at 1 µM for 72 hours. Bar graphs illustrate the percent viability over the corresponding DMSO control (n=4, duplicates for each condition). **F** Venn diagram showing the overlap of drugs that are not toxic to either neurons or astrocytes, considering only significant decreases in viability as determined in E. Data are shown as mean ± SD (E). Statistics are derived from two-tailed unpaired t tests with Welch's correction (D), and one-way ANOVA with Dunnett correction (E).

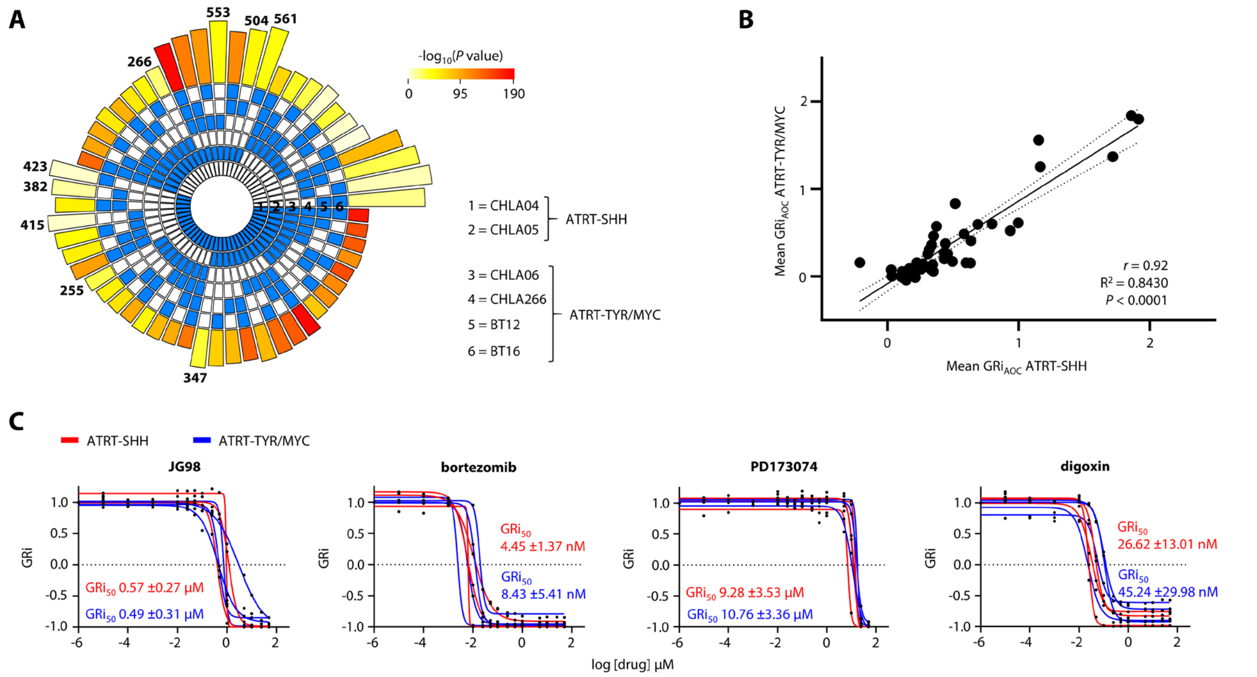

Figure S6. Extended interaction analyses of genetic/chemical vulnerabilities and molecular subgroups of ATRT. A Multi-layer circular plot illustrating all possible intersections of context-specific essential genes from six ATRT cell lines. The inner panels show absence (white) or presence (blue) of a specific cell line in a given intersection. The outer bar height represents the intersection size, and the bar color intensity represents statistical significance. Subgroup affiliation of ATRT cell lines is shown. **B** Scatterplot showing the correlation of mean GRi_{AOC} values from ATRT-SHH and ATRT-TYR/MYC cell lines for all 44 small molecules from the ATRT drug library tested in a three dose drug screen (see Fig. 2). **C** 15 point GRi dose response curve analyses for selected small molecules in ATRT-SHH (CHLA02, CHLA04, CHLA05) and ATRT-TYR/MYC (BT12, BT16, CHLA06, CHLA266) cell lines. Mean GRi_{50} values for ATRT-SHH and ATRT-TYR/MYC subgroup cell lines are shown. Statistics are derived from a SuperExactTest (A), and two-tailed unpaired t test (B).

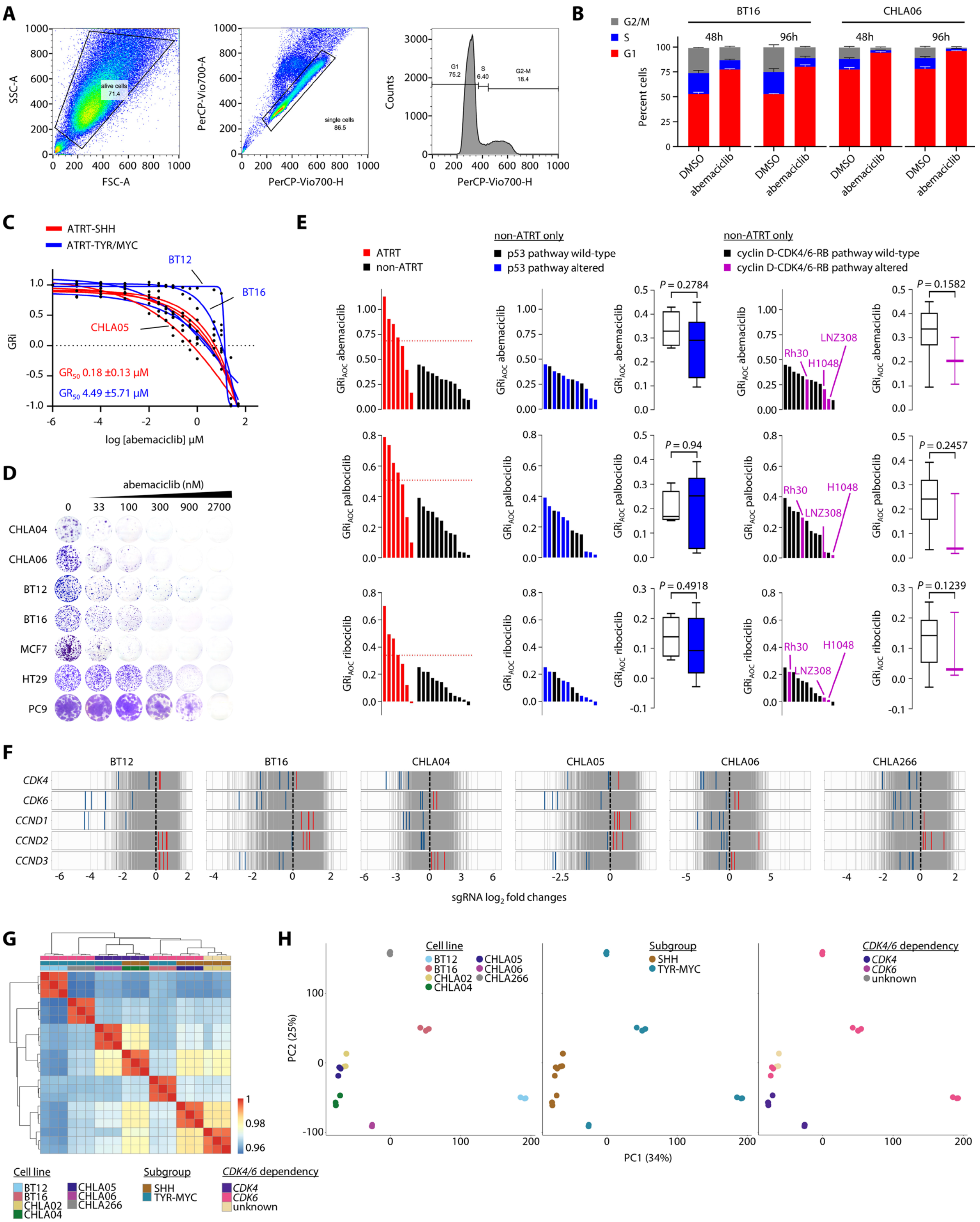

Figure S7. Analyses for CDK4/6 inhibitor sensitivity in ATRT cells. A Exemplary gating strategy for propidium iodide staining of DMSO-treated BT16 cells. Left, exclusion of cell debris based on forward (FSC-A) and sideward (SSC-A) scatter. Middle, identification of singlets using area and height of the fluorescence channel PerCP-Vio700. Right, Histogram of fluorescence counts to determine fraction of cells in distinct cell cycle phases. **B** FACS analyses using PI staining illustrating cell cycle phase shift after 200 nM abemaciclib treatment in ATRT cell lines. **C** 15-point GRi dose-response-curve analyses for the CDK4/6 inhibitor abemaciclib in ATRT-SHH (CHLA02, CHLA04, CHLA05, ATRT-311FHTC) and ATRT-TYR/MYC (BT12, BT16, CHLA06, CHLA266) cell lines. **D** Colony-formation assays of ATRT (CHLA04, CHLA06, BT12, BT16) and selected non-ATRT cell lines (MCF7, HT29, PC9) treated with abemaciclib. **E** Bar graphs associating GRi_{AOC} values from

the drug screen shown in Fig. 2 for CDK4/6 inhibitors abemaciclib (top), palbociclib (middle), and ribociclib (bottom) with known pathway alterations. Color-coded are known alterations in the p53 pathway (blue) and cyclin D-CDK4/6-RB pathway (magenta) in the non-ATRT cell lines. **F** Rank plots of log_2 fold changes for sgRNAs targeting *CDK4*, *CDK6*, *CCND1*, *CCND2*, and *CCND3* in six ATRT dependency screens. Vertical grey lines illustrate sgRNAs targeting known non-essential genes. Vertical black dashed lines indicate a log2 fold change of zero. **G** Pairwise correlations of ATRT cell lines according to gene expression. Molecular subgroups and dependency on either *CDK4* or *CDK6* are indicated. **H** Principal component analysis for ATRT cell lines according to gene expression. Cell line identities, molecular subgroups, and *CDK4* or *CDK6* dependencies are indicated. Statistics are derived from two-tailed unpaired t tests with Welch's correction (E).

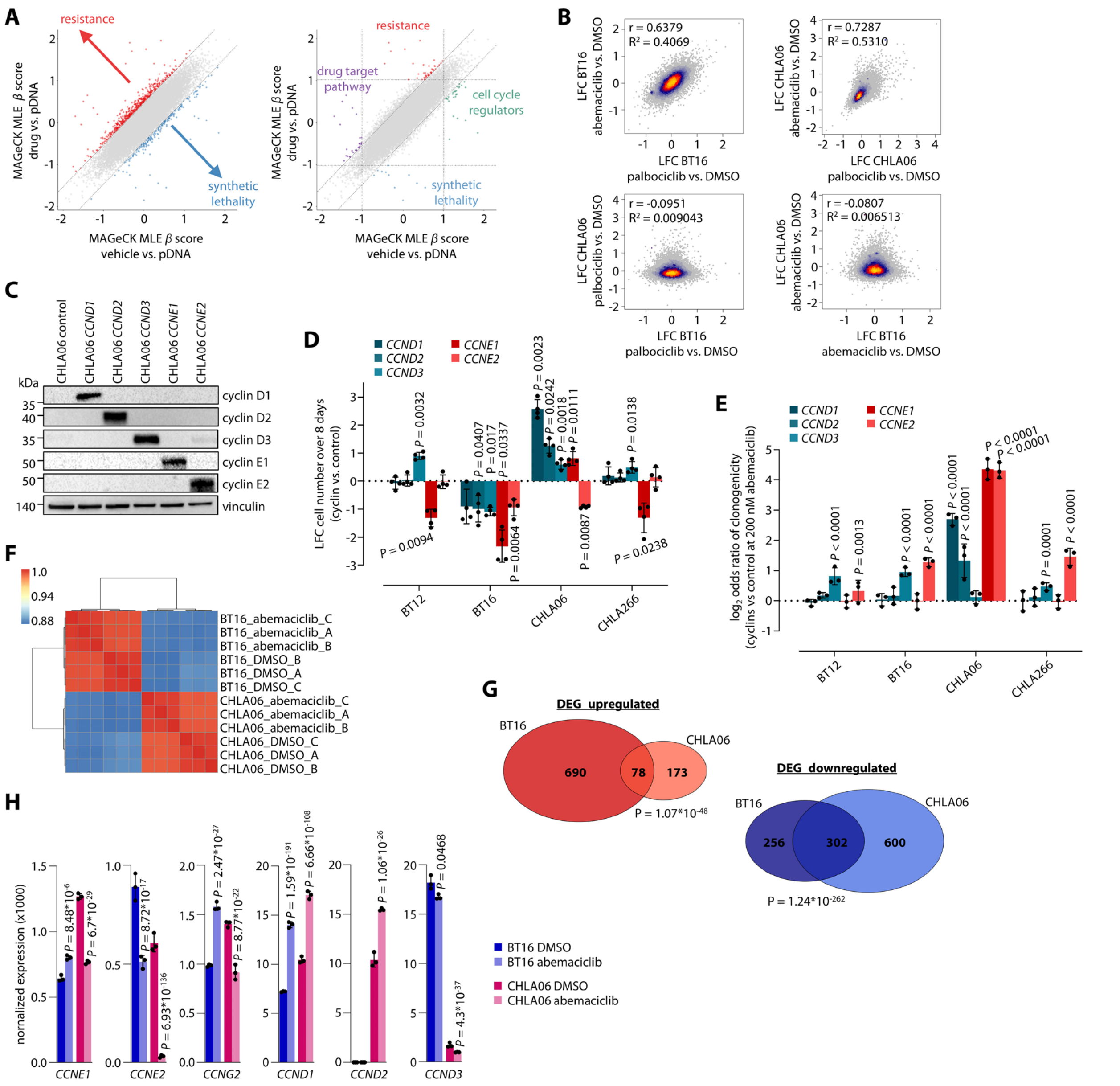

Figure S8. Gain-of-function CRISPR drug screens and transcriptional analysis of CDK4/6 blockade effects in ATRT cells. A Simulated results from CRISPR-Cas9 drug screens. Left, correlation of *β* scores from drug/vehicle comparisons to pDNA using MAGeCK MLE. Genes can be grossly categorized in hits conferring resistance or synthetic lethal hits. Right, nine square plot of the same data further distinguishing between cell cycle regulators and potential hits within the pathway that is targeted by the drug. **B** Heat scatter illustrating the correlation of gene level log_2 fold changes from CRISPR-Cas9 gain-of-function drug screens using distinct CDK4/6 inhibitors in the same cell line, or correlation of screen data using the same CDK4/6 inhibitor, but in different cell lines. **C** Exemplary western blot verification of overexpression of G1 phase cyclins in CHLA06 cells. **D** Effect of overexpression of distinct G1 phase cyclins on the proliferation of ATRT cells as measured by log_2 fold change in cell number over 8 days for the corresponding cell type compared to control cells. **E** log_2 odds ratio of number of colonies in cyclin overexpressing and corresponding control cells under DMSO and abemaciclib treatment (200 nM). **F** Pairwise correlation heat map and clustering for gene expression data from BT16 and CHLA06 cells treated with 200 nM abemaciclib or corresponding DMSO. **G** Venn diagrams illustrating the overlap of differentially down- or upregulated genes in ATRT cells upon CDK4/6 blockade. **H** Baseline expression and directionality of gene expression changes as a result of CDK4/6 blockade in BT16 and CHLA06 cells for selected cyclins. Statistics are derived from paired t tests (D), Fisher's exact test (E), a SuperExactTest (G), and Wald test (H).

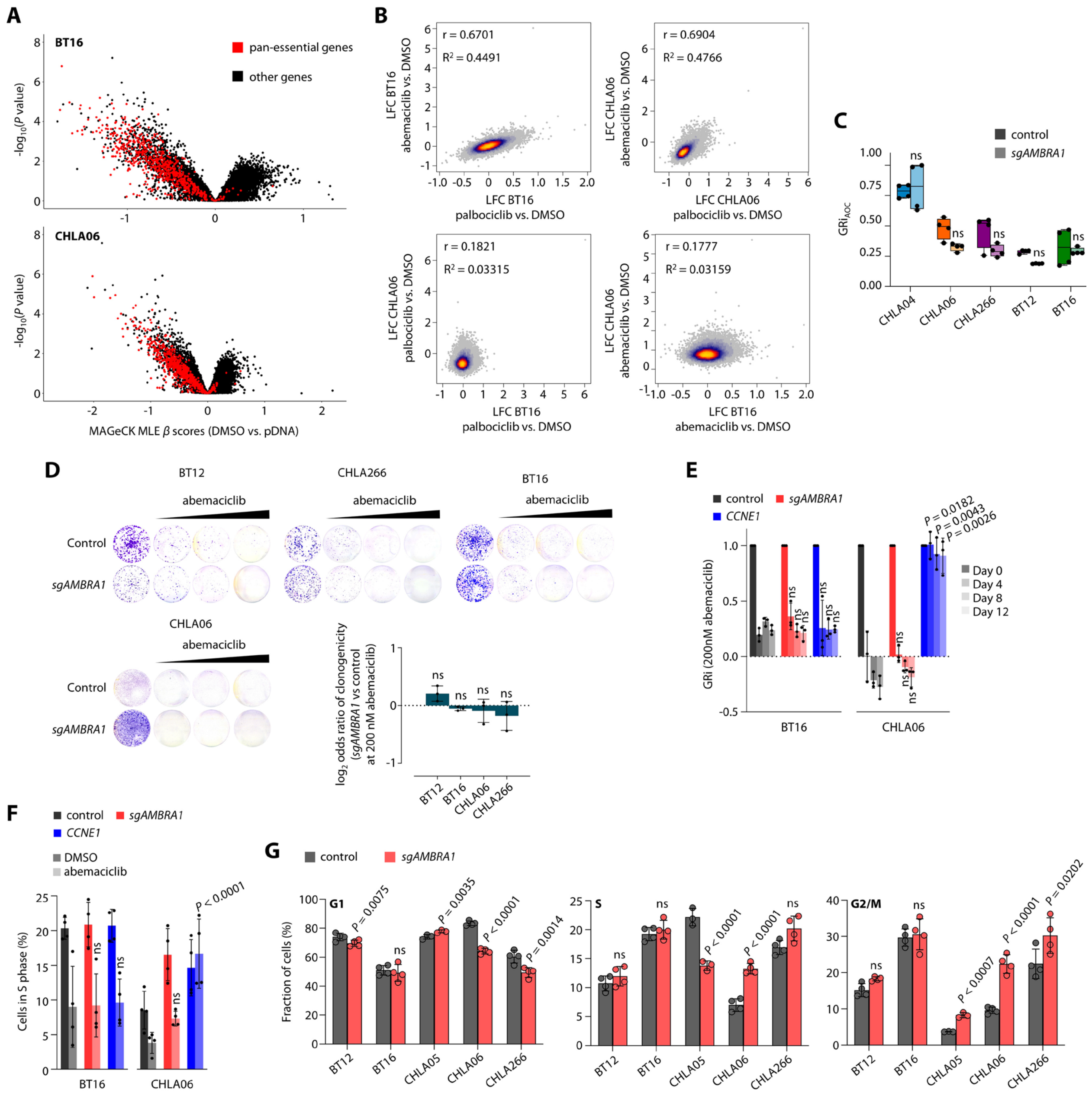

Figure S9. Loss-of-function CRISPR drug screens and validation of *AMBRA1* as screen hit. A Volcano plots validating depletion of known pan-essential genes in DMSO arms from genome-wide loss-of-function screens in BT16 and CHLA06 cells. **B** Heat scatter illustrating the correlation of gene level log_2 fold changes from CRISPR-Cas9 loss-of-function drug screens using distinct CDK4/6 inhibitors in the same cell line (two plots on the left), or correlation of screen data using the same CDK4/6 inhibitor but in different cell lines (two plots on the left). **C** Box whisker plot showing GRi_{AOC} for dose response curve analyses of ATRT cell lines treated with abemaciclib. Whiskers extend from maximum to minimum values. **D** Analyses of clonogenic survival of ATRT cell lines under increasing concentrations of abemaciclib (200 nM to 800 nM). Exemplary pictures of clonogenic survival plates from either control or *sgAMRBA1* ATRT cells are shown. Quantification as log_2 odds ratio of number of colonies in *AMBRA1* knockout and corresponding control cells under DMSO and abemaciclib treatment (200nM). **E** Bar graphs of GRi values for BT16 and CHLA06 cells as control, loss of *AMBRA1* (sgAMBRA1), and overexpression of *CCNE1* (*CCNE1*) treated with 200 nM abemaciclib for a total of 12 days. *P* values denote statistical difference in GRi values for *sgAMBRA1* and *CCNE1* conditions compared to control for the corresponding day. Interaction *P* value for treatment duration and genotype condition is P = 0.3121 (BT16) and P < 0.0001 (CHLA06). **F** Cell cycle analysis from PI staining in FACS analyses showing percentage of S phase cells for control, *sgAMBRA1* and *CCNE1* ATRT cells treated with 200 nM abemaciclib for 24 hours. *P* values denote statistical difference in fraction of S phase cells in *sgAMBRA1* and *CCNE1* conditions compared to control for the corresponding treatment condition (DMSO or abemaciclib). Interaction *P* value for genotype condition and treatment is P = 0.9809 (BT16) and P = 0.001 (CHLA06). **G** Bar graphs illustrating changes in cell cycle distributions in G1, S, and G2/M phase in several ATRT cell lines with and without loss of *AMBRA1* gene expression. Statistics are derived from maximum likelihood estimation (A), two-way ANOVA tests with Bonferroni's correction (C, E, G), fisher's exact test (D), and two-way ANOVA with Tukey correction (F).

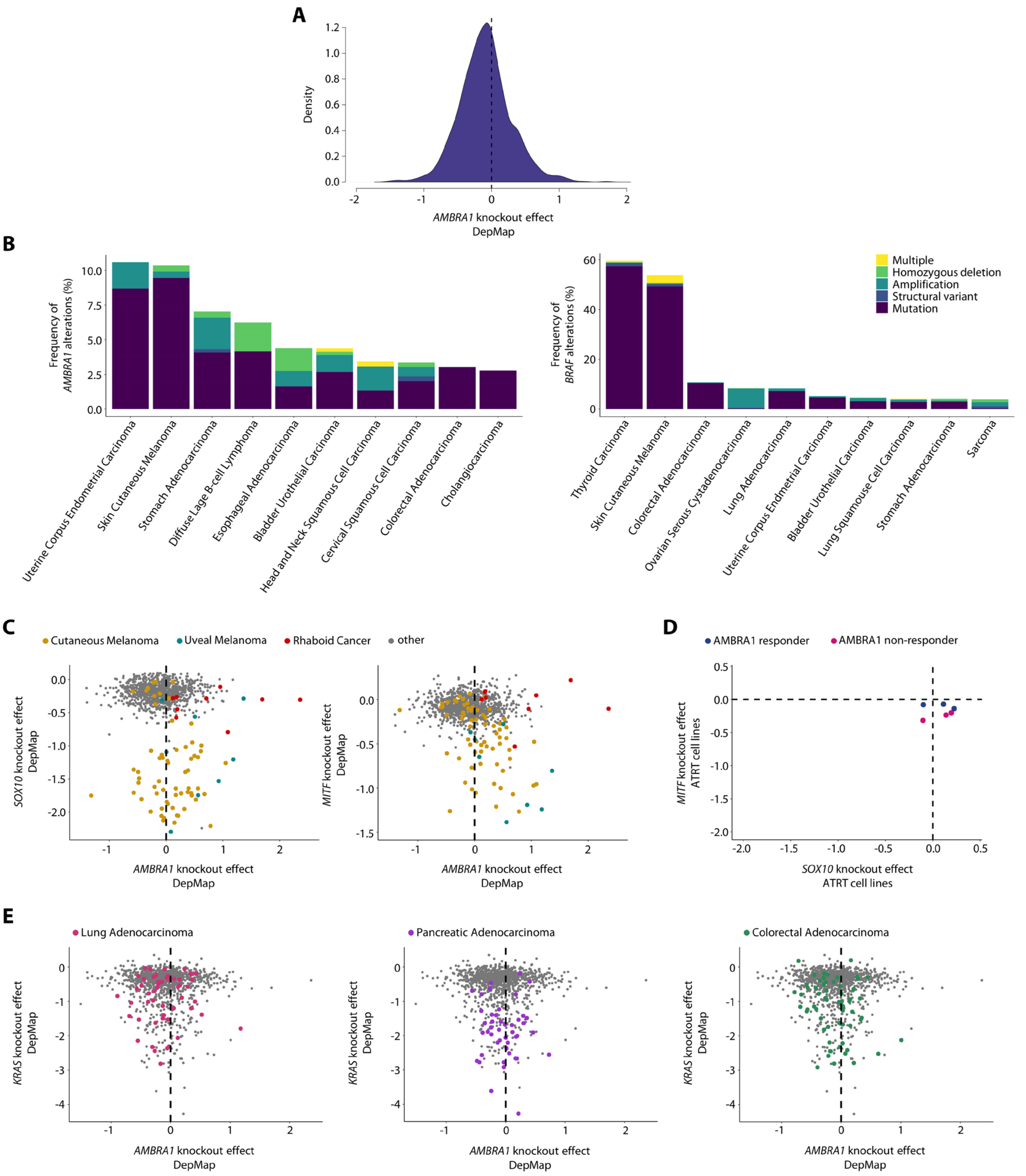

Figure S10. AMBRA1 gene effect associations from DepMap and mutation frequency. A *AMBRA1* knockout effect across 1,150 human cancer cell lines from DepMap. **B** Frequency of AMBRA1 (left) and BRAF (right) alterations in 10,967 tumor tissue samples from the TCGA PanCancer Atlas Studies. The top 10 tumor entities are shown. **C** Correlation of *SOX10* or *MITF* and *AMBRA1* gene knockout effects in melanoma and rhabdoid cancer cell lines. Data for all other cell lines in DepMap are shown as comparison. **D** Scatter plot illustrating the effect of *SOX10* and *MITF* knockout in ATRT cell lines. **E** Correlation of *KRAS* and *AMBRA1* knockout effects in lung, pancreatic, and colorectal adenocarcinoma cell lines from DepMap.

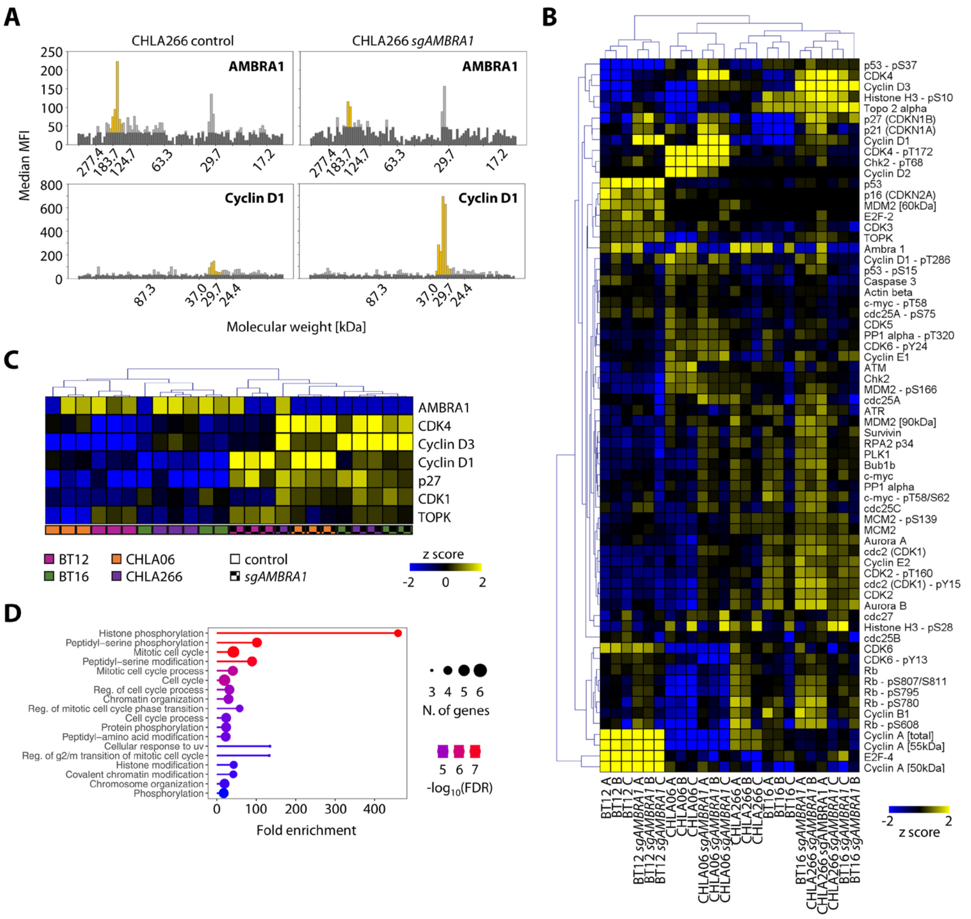

Figure S11. Changes in protein abundance upon loss of *AMBRA1* in ATRT cells. A Exemplary DigiWest spectra for AMBRA1 and cyclin D1 protein levels in control and *sgAMBRA1* CHLA266 cells. Signal spectra at the predicted size are highlighted in yellow. **B** Heat map illustrating protein levels as determined by antibodies for total and phosphorylated forms of a total of 39 cell cycle-associated antigens in control and *sgAMBRA1* ATRT cells. **C** Heat map illustrating significantly different log_2 fold changes in protein levels upon loss of *AMBRA1* in ATRT cell lines. **D** Lollipop plot showing results from gene ontology analyses using the GO Biological Process database for all genes significantly upregulated by loss of *AMBRA1* in AMBRA1 responder ATRT cells. Statistics are derived from Welch's t test (C), and hypergeometric test (D).

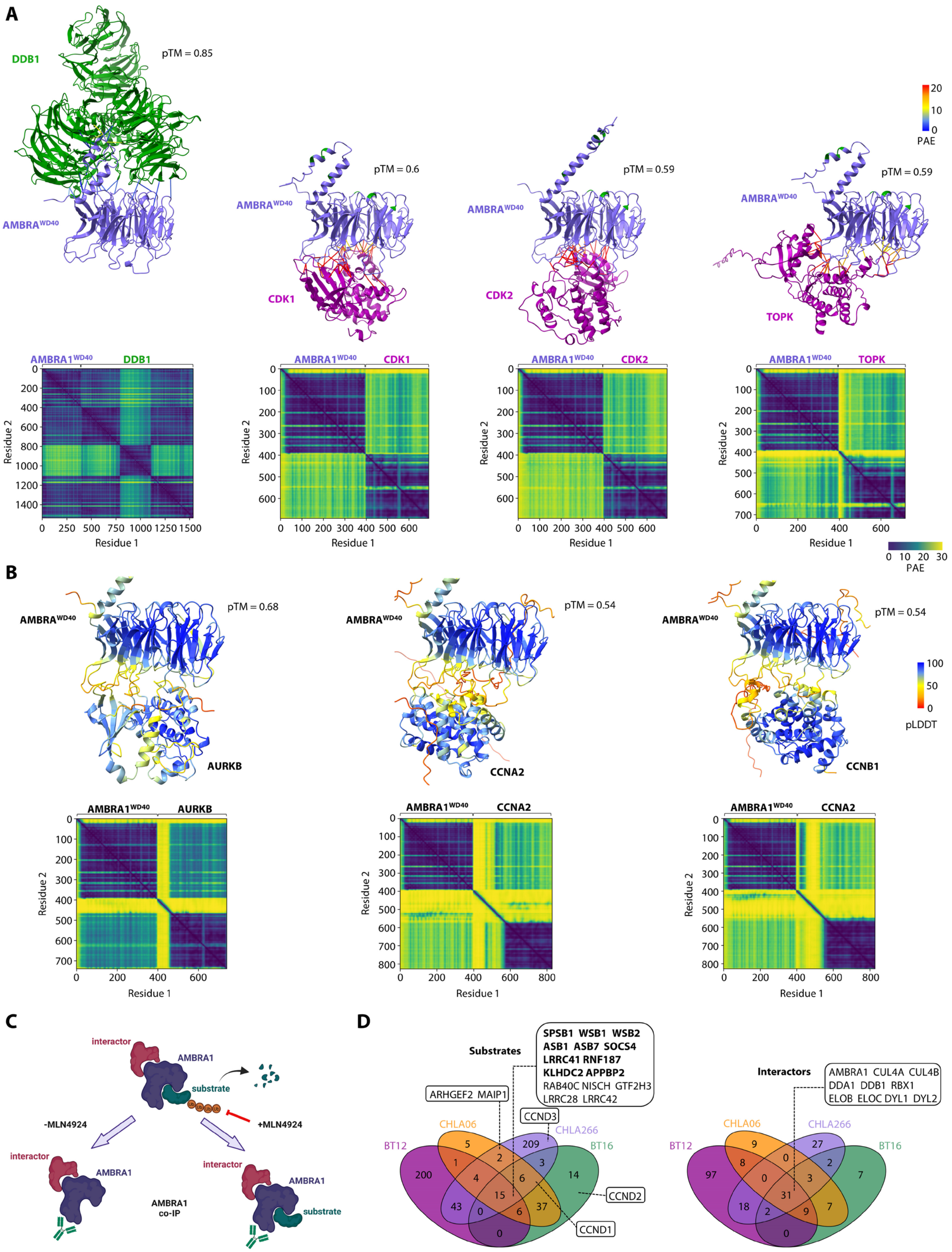

Figure S12. Interactome analysis for AMBRA1 in ATRT cells. A Protein complex structure prediction using AlphaFold2_mmseqs2 for AMBRA1^{WD40} with DDB1, CDK1, CDK2, and TOPK. Top, cartoon illustration of predicted heteromeric protein complexes. Interchain AlphaFold2 contacts of less than eight Angstroms are shown as straight lines colored by predicted alignment error (PAE). AMBRA1 residues previously implicated in DDB1 binding are highlighted in green. Predicted template modeling scores (pTM) for all complexes are indicated. Bottom, pairwise

PAE scores for all protein complexes shown. **B** Protein complex structure prediction using AlphaFold2_mmseqs2 for AMBRA1^{WD40} with AURKB as compared to negative controls CCNA2 and CCNB1. Top, cartoon illustration of predicted heteromeric protein complexes. Per-residue confidence pLDDT metric is color-coded. pTM scores for all complexes are indicated. Bottom, pairwise PAE scores for all protein complexes shown. **C** Schematic illustrating the experimental setup to differentiate between interactors and substrates of AMBRA1 by affinity purification coupled with mass spectrometry. **D** Venn diagrams illustrating the overlap of identified interactors and substrates across 4 ATRT cell lines. Substrates indicated in bold are predicted to act as substrate receptors for CUL2/5-RING E3 ubiquitin ligases.
