# Supplementary figures and images for: Functional screening reveals genetic dependencies and diverging cell cycle control in atypical teratoid rhabdoid tumors
Source: Genome Biol. 2024 Dec 2;25:301. doi: 10.1186/s13059-024-03438-w (PMC11610224; doi:10.1186/s13059-024-03438-w)

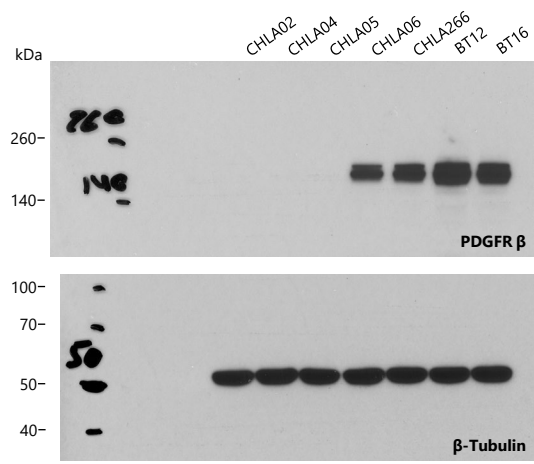

**Additional file 14:** Uncropped western blots for Figure S1D.

Supplement: Supplementary file 14 — Additional file 14: Uncropped western blots for Figure S1D. [file 13059_2024_3438_MOESM14_ESM.pdf]

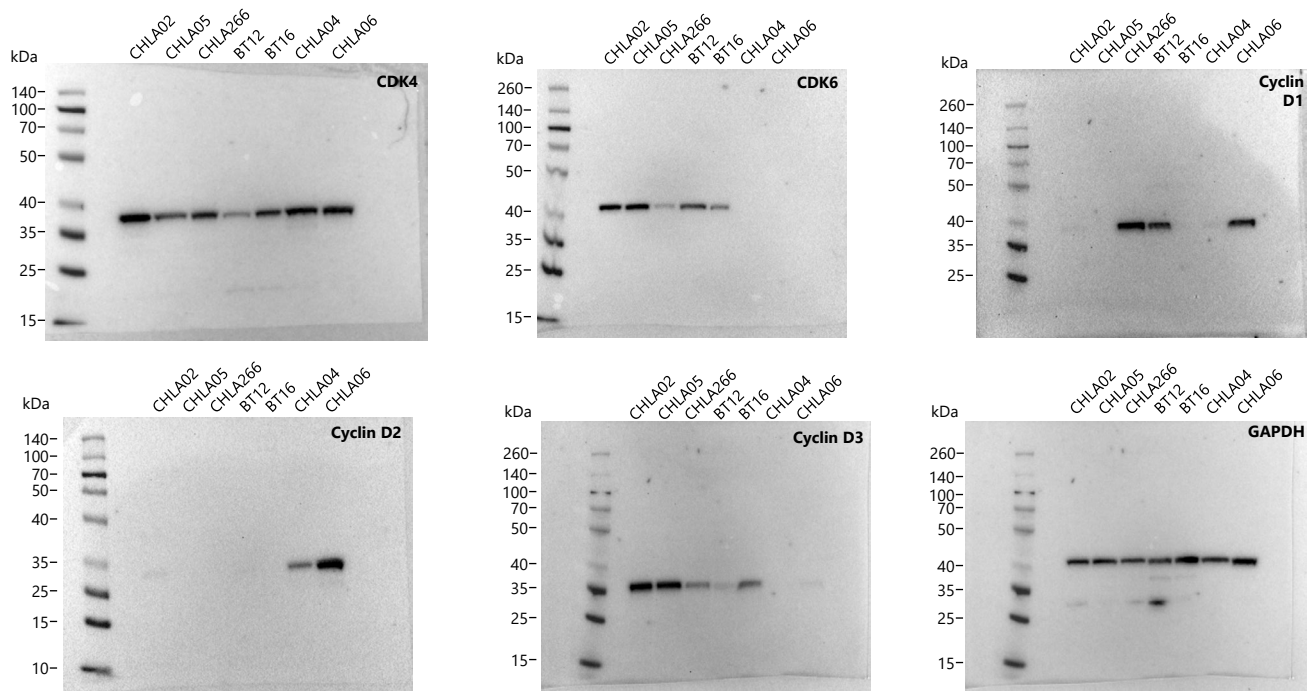

**Additional file 15:** Uncropped western blots for Figure 3D.

Supplement: Supplementary file 15 — Additional file 15: Uncropped western blots for Figure 3D. [file 13059_2024_3438_MOESM15_ESM.pdf]

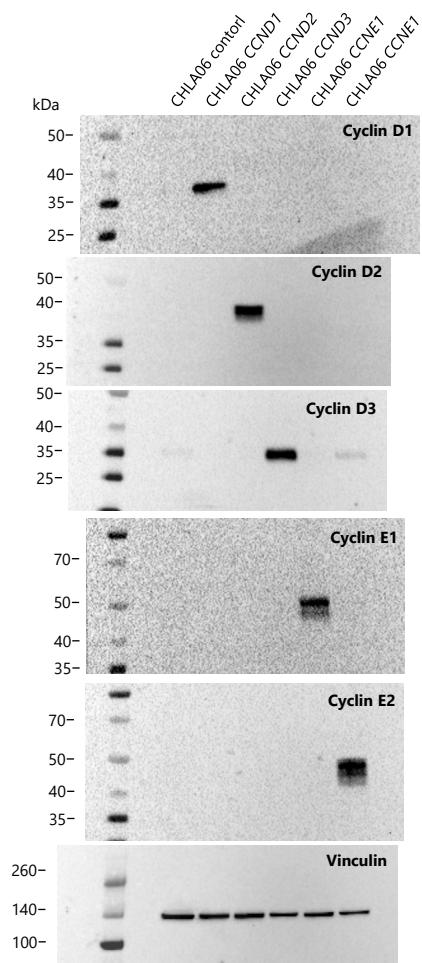

**Additional file 16:** Uncropped western blots for Figure S8C.

Supplement: Supplementary file 16 — Additional file 16: Uncropped western blots for Figure S8C. [file 13059_2024_3438_MOESM16_ESM.pdf]

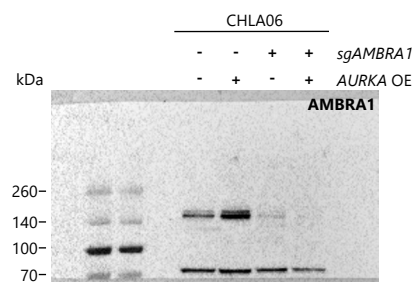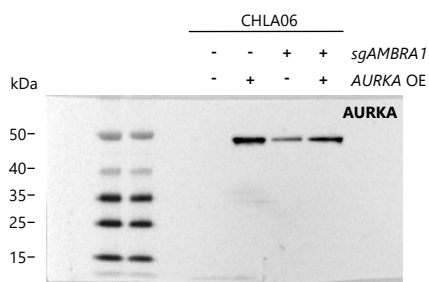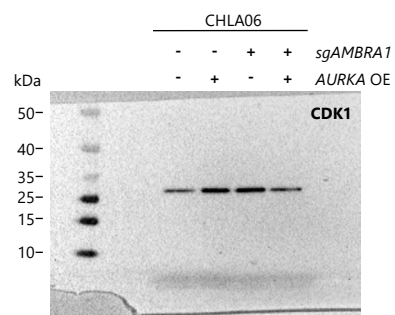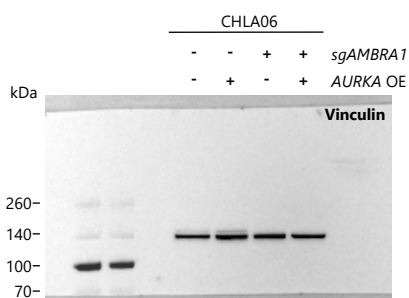

**Additional file 17:** Uncropped western blots for Figure 6B.

Supplement: Supplementary file 17 — Additional file 17: Uncropped western blots for Figure 6B. [file 13059_2024_3438_MOESM17_ESM.pdf]

**A**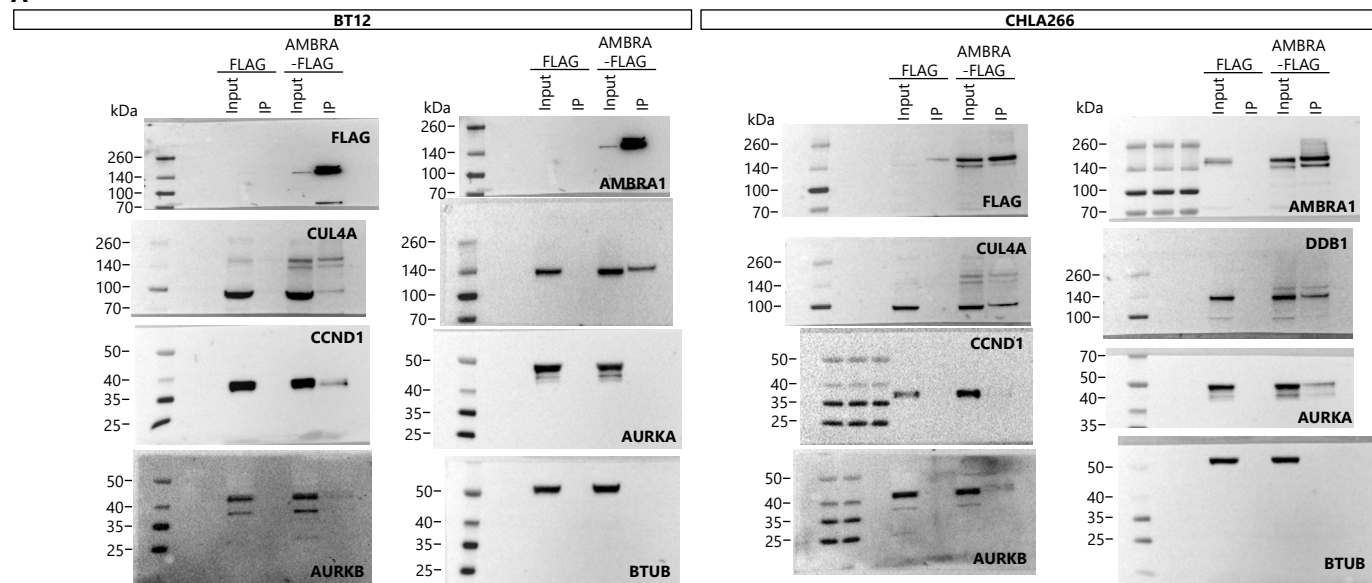**B**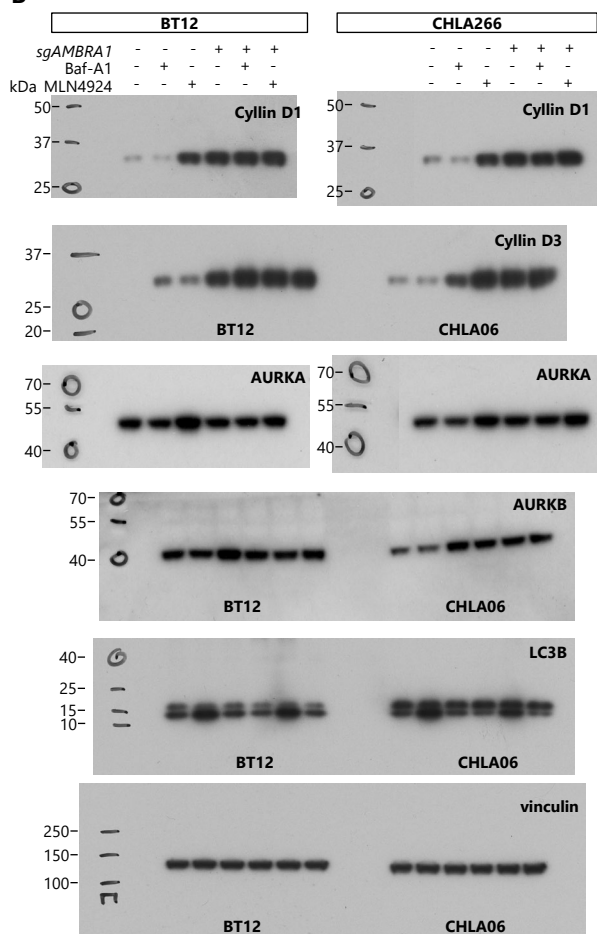**C**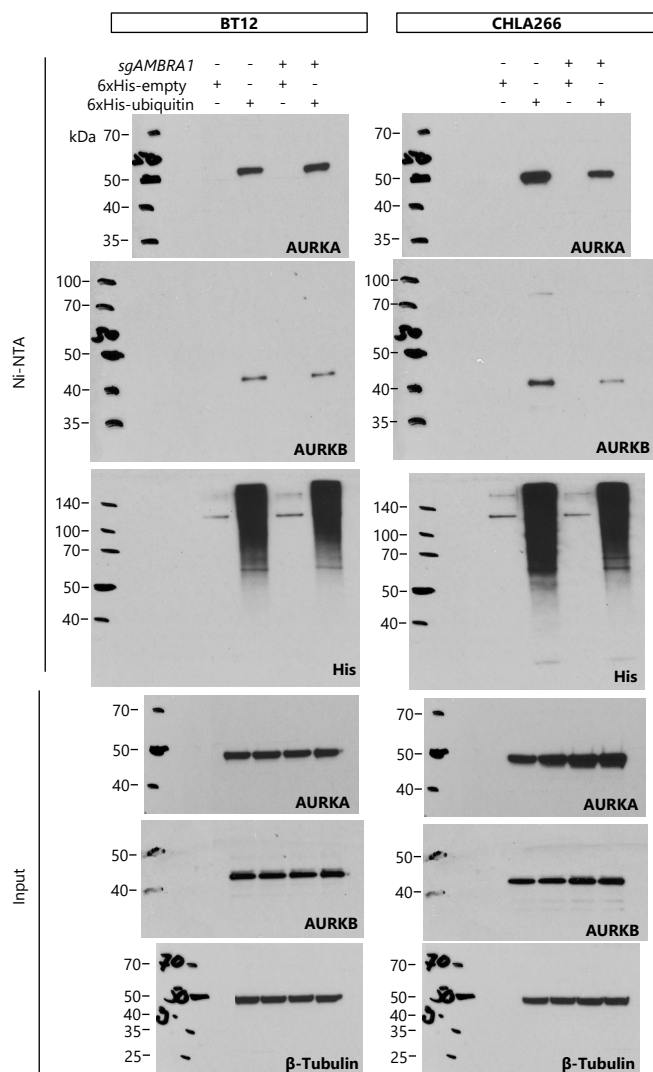

**Additional file 18:** Uncropped western blots for Figure 7C (A), Figure 7D (B), and Figure 7E (C).

Supplement: Supplementary file 18 — Additional file 18: Uncropped western blots for Figure 7C-E. [file 13059_2024_3438_MOESM18_ESM.pdf]
