# Supplementary material for: Functional screening reveals genetic dependencies and diverging cell cycle control in atypical teratoid rhabdoid tumors
Source: Genome Biol. 2024 Dec 2;25:301. doi: 10.1186/s13059-024-03438-w (PMC11610224; doi:10.1186/s13059-024-03438-w)
Supplement: Supplementary file 19 — Additional file 19: Review history. [file 13059_2024_3438_MOESM19_ESM.docx]

Review History

**First round of review**

**Reviewer 1**

**Were you able to assess all statistics in the manuscript, including the appropriateness of statistical tests used?**

Yes, and I have assessed the statistics in my report.

**Comments to author:**

Atypical teratoid rhabdoid tumors (ATRT) are fast-growing tumors typically found in the brain stem or cerebellum and are characterized by a lack of actionable mutations. The authors of this study uncover a preferential sensitivity of ATRT cells to the inhibition of specific dependencies, including differential dependencies for cell cycle regulators upstream pRB. Mechanistically, they identify diverging roles for G1 phase cyclins in regulating cell cycle progression, finding significant correlations between low CDK6 expression and high cyclin D2 expression, as well as changes in the expression levels of E- and D-type cyclins upon CDK4/6 inhibition. These observations lead to important conclusions about the modulation of ATRT cell responses to CDK4/6 blockade. The authors highlight a novel mechanism involving the ubiquitin ligase receptor AMBRA1, previously known as a regulator of D-type cyclin stability and the G1 to S phase transition. They provide new insights into AMBRA1's context-specific tumor suppressor role, particularly its regulation of key components of the G2/M phase. The study reveals a previously undescribed role for AMBRA1 in regulating cell cycle progression beyond D-type cyclin stability.
The study demonstrates high scientific rigor and robust independent verification experiments. It is built on an outstanding design for bioinformatic analysis, complemented by in vitro and in vivo verification. Some examples include the use of genome-wide CRISPR-Cas9 knockout screens followed by chemogenetic CRISPR screens to identify cell line dependencies. The authors employ eighteen genome-wide CRISPR-Cas9 knockout screens in six ATRT cell lines, targeting 19,114 genes, providing a comprehensive approach given that the cell line panel covers alterations in various cell cycle regulators. Additionally, the library of drug candidates is tested in seven ATRT cell lines and twelve non-ATRT control cancer cell lines, enabling robust identification of drugs with a preferred activity profile in ATRT cells. Chemogenetic CRISPR-Cas9 screens are conducted in both gain-of-function and loss-of-function modes to identify modulators of the response to CDK4/6 inhibition, ultimately identifying the ubiquitin ligase receptor AMBRA1. The authors also explore proteins interacting with AMBRA1 to elucidate the underlying mechanism.
The authors of this study present a comprehensive approach to discovering and validating molecular-guided treatment strategies for ATRT. Conceptually, this strategy has the potential to inform research on other diseases lacking actionable genetic mutations, aiding in molecular-based patient stratification and molecular-driven therapies. The findings are of significant interest to the readership due to their high scientific value and should be highly prioritized for publication.

Minor suggestions:
- On page 14, when the authors discuss dependency, it would be clearer to use the term "dependency score." As the authors explain, "CDK4 dependency is associated with high CCND2/cyclin D2 expression." Referring to the score will enhance clarity.
- The first paragraph of the Discussion should focus on the scientific results rather than the methodology, as this is not a methods development paper.
- "location-related epigenetic or transcriptional features defining ATRT subgroups"... There may be epigenetic or transcriptional features related to the molecular mechanisms that the authors discovered. It might be more accurate to say "rather than a consequence of age and epigenetic or transcriptional features defining ATRT subgroups in previous studies," considering the authors analyzed correlations with DNA methylation patterns and SMARC status.

**Reviewer 2**

**Were you able to assess all statistics in the manuscript, including the appropriateness of statistical tests used?**

Yes, and I have assessed the statistics in my report.

**Comments to author:**

This manuscript presents an in-depth functional analysis of atypical teratoid rhabdoid tumors (ATRT), an incurable pediatric brain tumor. By combining genome-wide CRISPR knockout (KO) and small molecule drug screening in various ATRT cell lines, the authors identified a preferential dependency of ATRT on CDK4/6 inhibitors. Furthermore, the study demonstrated that diverging cell cycle programs across cell lines predispose them to CDK4/6 inhibitors. Mechanistically, the authors identified AMBRA1 as a potential cell type-specific tumor suppressor gene (TSG) by regulating aurora kinase (AURKA).

Given the difficulty in treating ATRT and the extensive data generated, this study is valuable. However, the current version seriously suffers from presentation and logical flow issues.

Major concerns:

1. The manuscript is too long and complicated. The authors should streamline the flow.
a. The conclusion of Fig. 2 is expected and could be shortened. The synthetic lethality of MDM2 and MDM4 is not mentioned in subsequent sections. Additionally, Fig. 2B and Fig. 2C provide similar information; one could be moved to supplementary figures.
b. Some figures in Fig. 5A-C are recommended to be moved to supplementary figures.
c. Fig. 4 can be substantially simplified as Fig. 3B already indicates the chemical insensitivities among different ATRT subgroups.
d. To me, the content related to AMBRA1 and AURKA, specifically Figs. 7-9, seems more interesting. The material related to Figs. 1-6 could be shortened.

2. Logical Gaps and Ambiguities:
a. In Fig. 3B, a known ATRT cell line, BT12, clusters with non-ATRT cell lines. Authors should provide an explanation.
b. Fig. 3D-E shows that EGFR inhibitors and CDK4/6 inhibitors seem equally efficient. Authors should explain why they focus solely on CDK inhibitors.
c. In Fig. 5F, the dependency of the CHLA02 cell line on CDK is not mentioned in the main text or legend. What information does this cell line convey?
d. In Fig. 6A (two right panels), the effects of CCNE1 and CCND1 are opposite. However, in Page 16, Lines 369-370 and Fig. 6B-C, the authors argue that these two genes have similar roles.
e. In Fig. 9B, the authors argue that CHLA06 and BT16 sgAMBRA1 data bars are different. However, their patterns are quite similar despite different statistical signals. More replicate experiments are needed to confirm this contrast.

3. The authors argued for synthetic lethality of AURKA and CDK4/6 inhibition upon loss of AMBRA1. It is important to determine how often AMBRA1 is lost in ATRT patients. If genome sequencing data from projects like TCGA shows frequent loss of AMBRA1 in patients, the authors' discovery would be more significant for treatment insights.

Minor concerns:

1. Page 5, Line 108: "next generating sequencing" should be "next generation sequencing".
2. Are the different ATRT cell lines varied in terms of cell-of-origin?
3. In Fig. 2E, the CPM method may be outdated for transcriptome quantification; TPM is a better parameter.
4. For Fig. 3B, it would be better to mark drugs and cytotoxic agents in different colors.
5. For Fig. 4C, the y-axis label should be corrected from GRAOC ◊ GRiAOC
6. Fig. 5D shows that CHLA266 is not significantly dependent on D-type cyclin, making it inappropriate to state in Lines 310-312 that "all ATRT cell lines ...".
7. Page 13, Line 301: "response the" should be "response to the".
8. Page 18, Line 409: "cells lines" should be "cell lines".
9. Instead of merely citing DGIdb, the Methods section should include an analytical detail explaining whether the connected gene/drug pair was built upon specific tissue/cell type.

**Reviewer 3**

**Were you able to assess all statistics in the manuscript, including the appropriateness of statistical tests used?**

No, I do not feel adequately qualified to assess the statistics.

**Comments to author:**

In the manuscript entitled "Functional screening reveals genetic dependencies and diverging cell cycle control in atypical teratoid rhabdoid tumors," Merck et al. used CRISPR-based genetic screens to find vulnerabilities in atypical teratoid rhabdoid tumors (ATRTs). The authors then validated certain hits with small molecule drug library and homed in on G1-cell cycle regulators, with a focus on CDK4/6 inhibitors. The authors further show that 'ubiquitin ligase substrate receptor AMBRA1 acts as a context-specific inhibitor of cell cycle progression.' Overall, this work is of value to the field and will be of interest to readers of Genome Biology after addressing the following comments.

Major comments:

Based on their known importance, it would be likely that some of the 9 compounds tested for toxicity in vitro (ex. The inhibitors of CDK4/6, MAPK, and EGF) would have clinical or preclinical in vivo toxicity information available that should be noted/compared to.

To make the claim that mutated KRAS/BRAF tumors are likely sensitive to AMBRA1 loss, the authors should include an analysis of available large-scale data sets as support.

AlphaFold multimer structures should be used with some caution. As a 'negative control,' the authors should predict the co complex of AMBRA and a cyclin protein homolog that AMBRA is not expected to interact with. If there is a similar interaction as seen with the included AMBRA interaction partners, then those predicted interactions cannot be trusted.

Minor comments:

(1) It's unclear of the subtype of the seven selected ATRT cell lines when they are introduced in the text. Please make note of this or move S1H to Fig1. As the authors also stress clinical relevance throughout, ATRT subtypes should be specified when discussing therapeutic response (e.g., p14 for CDK4/6 dependent cell lines).
(2) The word 'signaling' is often capitalized, which appears to be a typo.
(3) It is unclear what figures 2B and 2C are supposed to inform the reader of.

**Authors Response**

**Point-by-point responses to the reviewers’ comments:**

Reviewer #1:

Atypical teratoid rhabdoid tumors (ATRT) are fast-growing tumors typically found in the brain stem or cerebellum and are characterized by a lack of actionable mutations. The authors of this study uncover a preferential sensitivity of ATRT cells to the inhibition of specific dependencies, including differential dependencies for cell cycle regulators upstream pRB. Mechanistically, they identify diverging roles for G1 phase cyclins in regulating cell cycle progression, finding significant correlations between low CDK6 expression and high cyclin D2 expression, as well as changes in the expression levels of E- and D-type cyclins upon CDK4/6 inhibition. These observations lead to important conclusions about the modulation of ATRT cell responses to CDK4/6 blockade. The authors highlight a novel mechanism involving the ubiquitin ligase receptor AMBRA1, previously known as a regulator of D-type cyclin stability and the G1 to S phase transition. They provide new insights into AMBRA1's context-specific tumor suppressor role, particularly its regulation of key components of the G2/M phase. The study reveals a previously undescribed role for AMBRA1 in regulating cell cycle progression beyond D-type cyclin stability.

The study demonstrates high scientific rigor and robust independent verification experiments. It is built on an outstanding design for bioinformatic analysis, complemented by in vitro and in vivo verification. Some examples include the use of genome-wide CRISPR-Cas9 knockout screens followed by chemogenetic CRISPR screens to identify cell line dependencies. The authors employ eighteen genome-wide CRISPR-Cas9 knockout screens in six ATRT cell lines, targeting 19,114 genes, providing a comprehensive approach given that the cell line panel covers alterations in various cell cycle regulators. Additionally, the library of drug candidates is tested in seven ATRT cell lines and twelve non-ATRT control cancer cell lines, enabling robust identification of drugs with a preferred activity profile in ATRT cells. Chemogenetic CRISPR-Cas9 screens are conducted in both gain-of-function and loss-of-function modes to identify modulators of the response to CDK4/6 inhibition, ultimately identifying the ubiquitin ligase receptor AMBRA1. The authors also explore proteins interacting with AMBRA1 to elucidate the underlying mechanism.

The authors of this study present a comprehensive approach to discovering and validating molecular-guided treatment strategies for ATRT. Conceptually, this strategy has the potential to inform research on other diseases lacking actionable genetic mutations, aiding in molecular-based patient stratification and molecular-driven therapies. The findings are of significant interest to the readership due to their high scientific value and should be highly prioritized for publication.

*Response:*

*We thank this reviewer for this very positive and appreciative overall assessment.*

Minor suggestions:

- On page 14, when the authors discuss dependency, it would be clearer to use the term "dependency score." As the authors explain, "CDK4 dependency is associated with high CCND2/cyclin D2 expression." Referring to the score will enhance clarity.

*Response:*

*We are grateful for this very helpful feedback and fully agree with this reviewer that referring to dependency scores enhances clarity, in particular when correlating these scores with gene/protein expression. We have thus followed this reviewer’s advice and now use the term “dependency scores” in this context, and this has been incorporated into the Results section on page 14 as follows:*

*“While CDK4/CDK4 levels were not associated with CDK4 essentiality, CDK4 dependency scores showed strong negative correlation with CCND2/cyclin D2 expression (i.e. ATRT cells dependent on CDK4 show high CCND2/cyclin D2 expression), with CDK4 dependent cells showing no detectable CDK6 protein expression. In contrast, CDK6 dependency scores showed strong negative correlation with CDK6/CDK6 expression, and were positively correlated with CCND2/cyclin D2 levels. Notably, these correlations are conserved across human cancer cell lines, where CDK6 expression is positively correlated with CDK4 dependency scores, while showing strong negative correlation with CDK6 dependency scores [25].”*

*Furthermore, we have adjusted the text in the Results section on pages 8 and 9 accordingly:*

*“**Of note, while we found both positive as well as negative correlations of gene expression features with gene dependency scores, our data revealed exclusive positive correlations of promoter methylation and dependency scores, where low promoter methylation levels that lead to higher gene expression are associated with gene dependency. Direct correlation of either gene expression or promoter methylation with gene dependency scores for context-specific fitness genes revealed a significant shift of correlation coefficients towards a more negative (gene expression) or positive (methylation) distribution as compared to the null distribution (Fig. 1G).”*

- The first paragraph of the Discussion should focus on the scientific results rather than the methodology, as this is not a methods development paper.

*Response:*

*We thank this reviewer for this comment, and fully agree that the Discussion section was too repetitive in terms of methodological detail. We have thus streamlined the first paragraph of the Discussion in the revised manuscript on pages 23 and 24 to focus on our own scientific results, and this paragraph reads as follows:*

*“In this study, we applied a functional genomics approach to ATRT, an incurable pediatric brain tumor, in order to define genetic vulnerabilities that can guide targeted therapies for this tumor entity. We here provide evidence for a particular sensitivity of ATRT to CDK4/6 inhibition, and investigate genetic modulators of response to CDK4/6 inhibitors. Surprisingly, our study reveals a very interesting and novel context-specific tumor suppressive activity of the autophagy regulator AMBRA1 in ATRT that depends on the negative regulation of G2/M phase mediators.”*

- "location-related epigenetic or transcriptional features defining ATRT subgroups"... There may be epigenetic or transcriptional features related to the molecular mechanisms that the authors discovered. It might be more accurate to say "rather than a consequence of age and epigenetic or transcriptional features defining ATRT subgroups in previous studies," considering the authors analyzed correlations with DNA methylation patterns and SMARC status.

*Response:*

*We agree with this reviewer and have incorporated the reference to previous studies within the Discussion section on pages 24 and 25 as follows:*

*“Together, these data suggest that chemical vulnerabilities in ATRT are a result of entity-specific molecular mechanisms, rather than a consequence of age and epigenetic or transcriptional features defining ATRT subgroups in previous studies. Yet, all these studies including ours are limited by the number of available cell lines representing individual ATRT subgroups, and novel model systems are warranted to further examine potential subgroup-specific differences.”*

Reviewer #2: This manuscript presents an in-depth functional analysis of atypical teratoid rhabdoid tumors (ATRT), an incurable pediatric brain tumor. By combining genome-wide CRISPR knockout (KO) and small molecule drug screening in various ATRT cell lines, the authors identified a preferential dependency of ATRT on CDK4/6 inhibitors. Furthermore, the study demonstrated that diverging cell cycle programs across cell lines predispose them to CDK4/6 inhibitors. Mechanistically, the authors identified AMBRA1 as a potential cell type-specific tumor suppressor gene (TSG) by regulating aurora kinase (AURKA).

Given the difficulty in treating ATRT and the extensive data generated, this study is valuable. However, the current version seriously suffers from presentation and logical flow issues.

Major concerns:

1. The manuscript is too long and complicated. The authors should streamline the flow.

a. The conclusion of Fig. 2 is expected and could be shortened. The synthetic lethality of MDM2 and MDM4 is not mentioned in subsequent sections. Additionally, Fig. 2B and Fig. 2C provide similar information; one could be moved to supplementary figures.

*Response:*

*We agree with this reviewer and now provide a shorter and more focused presentation of our results. In particular, we have merged previous Fig. 1 and Fig. 2 to now be shown in new Fig. 1, and have moved supporting panels from previous Fig. 1 and Fig. 2 to corresponding supplements in Fig. S2E, Fig. S3B,C and Fig. S4A,B,C. Showing all data on genetic dependencies and potential molecular predictors together in Fig. 1 greatly enhances the flow of the revised version of our manuscript.*

*The synthetic lethality of MDM2 and MDM4 in TP53 wild-type cancer cells served validation purposes of our screening approach in the original manuscript, recapitulating previously shown genetic interactions in human cancer cells [1], and has thus not been mentioned in subsequent sections. These findings provide supporting information with regard to the quality of our screens, and have thus been moved to Fig. S4A of the revised version of the manuscript.*

b. Some figures in Fig. 5A-C are recommended to be moved to supplementary figures.

*Response:*

*Again, we are grateful for this comment and agree with this reviewer. We have moved panels A and B from previous Fig. 5 to the supplements section in Fig. S7C,D. Due to the extensive rearrangement of figures during the revision, data originally shown in previous Fig. 5 is now depicted in Fig. 3.*

c. Fig. 4 can be substantially simplified as Fig. 3B already indicates the chemical insensitivities among different ATRT subgroups.

*Response:*

*Similar to our response to comment 1a above, we have followed this reviewer’s advice to simplify and streamline the flow of the paper. We have thus merged data regarding drug screening and associations with ATRT subgroups (previously shown in Fig. 3 and Fig. 4) to now be shown in new Fig. 2. Consequently, we have moved supporting panels from previous Fig. 3 and Fig. 4 to the supplements section in Fig. S5E,F and Fig. S6A-C.*

d. To me, the content related to AMBRA1 and AURKA, specifically Figs. 7-9, seems more interesting. The material related to Figs. 1-6 could be shortened.

*Response:*

*We thank this reviewer for this important comment.*

*While the aspects of our work focusing on the tumor suppressive functions of AMBRA1 are certainly of interest to the wider cancer research community, we are convinced that the wealth of information regarding genetic and chemical vulnerabilities in ATRT (Fig. 1-6 of the initial submission) is equally interesting to the ATRT research community. As suggested by this reviewer, we present all experiments within the first part more concisely in the revised version of the manuscript. In fact, we have simplified and shortened the first part of the manuscript on genetic and chemical vulnerabilities to now be presented in Fig. 1-4, while the second half of the manuscript (Fig. 5-8) is focusing on our intriguing findings on AMBRA1 tumor suppressive activity. We are convinced that this re-organisation of our revised manuscript has significantly improved the flow of the manuscript.*

2. Logical Gaps and Ambiguities:

a. In Fig. 3B, a known ATRT cell line, BT12, clusters with non-ATRT cell lines. Authors should provide an explanation.

*Response:*

*We do agree with this reviewer that BT12 cells appear to have a unique sensitivity profile when compared to all other ATRT cell lines based on GRi_AOC_, which can be regarded as kind of an average over the three drug concentrations employed in our drug screen. However, we want to emphasize at this stage that BT12 cells cluster with other ATRT cell lines when only considering the highest (1µM) concentration (Fig. S5B).*

*In terms of ATRT molecular subgroups, BT12 cells were most similar to CHLA06, CHLA266, and BT16 cells, and this is in line with previous reports [2]. However, on the global transcriptomic level, we here show that BT12 cells differ greatly from other ATRT lines on principal component 1 (Fig. S7G,H), and this might explain the distinct sensitivity profile. We note the differences in clustering and a potential association with a distinct gene expression profile on page 10 within the Results section as follows:*

*“Variable clustering of BT12 cells to either ATRT or non-ATRT cells at different drug concentrations might suggest distinct sensitivities of this cell line from other ATRT models (Additional file 1: Fig. S5B), and this is in line with a distinct gene expression profile in BT12 cells (Additional file 1: Fig. S7H).”*

b. Fig. 3D-E shows that EGFR inhibitors and CDK4/6 inhibitors seem equally efficient. Authors should explain why they focus solely on CDK inhibitors.

*Response:*

*While indeed EGFR inhibition may also represent an efficacious treatment option for ATRT according to our data, we had to prioritize a drug class in order to focus our experimental strategy of the present study. Furthermore, receptor tyrosine kinases had already been described before as targetable vulnerabilities in rhabdoid tumors [3]. On the other hand, it has long been appreciated that loss of SMARCB1 deregulates the* *cyclin D-CDK4/6-Rb axis in G1 phase of the cell cycle [4-6], suggesting that CDK4/6 inhibitors might directly inhibit a critical signaling axis relevant for ATRT. We added the following statement to the Results section to highlight this notion on page 12 as follows:*

*“Loss of SMARCB1 in rhabdoid tumors has been associated with deregulation of the cyclin D-CDK4/6-RB axis [30-32], and CDK4/6 inhibitors were among the inhibitors with the most promising activity profile in ATRT cells.”*

c. In Fig. 5F, the dependency of the CHLA02 cell line on CDK is not mentioned in the main text or legend. What information does this cell line convey?

*Response:*

*We thank this reviewer for this question. In fact, all CHLA02 screens performed poorly in all of our screen quality metrics. Thus, we decided to omit them from further screens analysis. We have attributed this poor performance to the fact that CHLA02 have the highest doubling time of all ATRT cell lines used in this study. Based on these observations, none of the correlation analyses such as those performed in new Fig. 3E include the CHLA02 cell line. However, we decided to include this line for the investigation of cyclin D and CDK4/6 protein levels in Fig. 3D for completeness, as it has also been included for all drug screening approaches in our study including those using our drug library (Fig. 2), as well as those focusing in particular on CDK4/6 inhibitors (Fig. S7). We now specifically note that there are no dependency profiles available for this cell line by amending the figure legend for Fig. 3D as follows:*

*“Note that profiles for CDK4 or CDK6 dependency were not available for CHLA02 cells.”*

d. In Fig. 6A (two right panels), the effects of CCNE1 and CCND1 are opposite. However, in Page 16, Lines 369-370 and Fig. 6B-C, the authors argue that these two genes have similar roles.

*Response:*

*We thank this reviewer for this important point, and we fully agree that this needs clarification. We do want to point out that data analysis now shown in Fig. 4A (previously Fig. 6A) illustrates consistent changes in screens treated with either abemaciclib or palbociclib, indicated as “CDK4/6 inhibitors” condition. On the other hand, phenotypic resistance effects in dose response or clonogenicity assays were only assessed for abemaciclib. Furthermore, while the screen data clearly favor a more cell cycle associated effect of CCND1 activation in CHLA06 cells rather than resistance (Fig. 4A, two right panels), it should be noted that CCND1 was slightly enriched in the CDK4/6 inhibitors vs. pDNA comparison (y axis on scatter plot), indicative of a potential resistance effect. Together, while all top hits in the screens behaved as expected in downstream validation, hits with a smaller effect size in screening such as CCND1 in this case might be more vulnerable to technical variation, both in the screens as well as in downstream validation. Still, the effect size of the resistance effect, in particular in clonogenicity assays, was much more pronounced for CCNE1 gain of function (Fig. 4C), which correlates well with the results from screening. Yet, we think this is an important point, and we modified the sentence on page 15 in the Results section as follows:*

*“Activation of CCNE1, and to a lesser extent CCND1 while not being a clear screen hit for a buffering effect, conferred resistance in CHLA06 but none of the other cell lines (Fig. 4B,C; Additional file 1: Fig. S8E).”*

e. In Fig. 9B, the authors argue that CHLA06 and BT16 sgAMBRA1 data bars are different. However, their patterns are quite similar despite different statistical signals. More replicate experiments are needed to confirm this contrast.

*Response:*

*We thank this reviewer for this important point. We added new data to this analysis which is now shown in Fig. 6C of the revised manuscript.*

*We would like to point out that our intention in the initial manuscript was to demonstrate that AURKA gain-of-function in both CHLA06 and BT16 cells enhanced proliferation on a sgAMBRA1 background. The corresponding sentence on lines 511 -513 of the initial manuscript was:*

*“…overexpression of AURKA enhanced proliferation only in AMBRA1 knockout cells which show uniform upregulation of D-type cyclins.”*

*In our view, this strongly suggested that AURKA, while not being able to significantly enhance cell proliferation in control CHLA06 and BT16 cells, it significantly enhances proliferation in cells that show upregulation of D-type cyclins, which is true both for CHLA06-sgAMBRA1 and BT16-sgAMBRA1 cells. As noted by this reviewer, this was shown already by our previous experiment where “patterns are quite similar” for both CHLA06 and BT12 sgAMBRA1 cells, though not significant for CHLA06-sgAMBRA1 cells.*

*We have now performed two additional iterations of this experiment, and together these data strongly support our previous data that overexpression of AURKA only enhances proliferation in ATRT cells that show strong upregulation of D type cyclins, i.e. both CHLA06-sgAMBRA1 and BT16-sgAMBRA1 cells (Fig. 6C). These data indicate that the effect is independent from the “AMBRA1 responder “phenotype, as long as D type cyclins are stabilized due to AMBRA1 loss.*

*We apologize for the lack of clarity in the initial manuscript in this regard. We have re-written our text within the Results section of the revised manuscript on pages 19 and 20 accordingly:*

*“These data suggest a model in which AMBRA1, beyond previously described actions on D-type stability alone, acts as a tumor suppressor by simultaneously regulating several cell cycle regulators at once. Consistent with this model, overexpression of AURKA enhanced proliferation both in AMBRA1-deleted ATRT responder (CHLA06) as well as non-responder (BT16) cells, while this was not the case in corresponding control cells (Fig. 6B,C). Together, stabilization of D-type cyclins as a result of AMBRA1 loss in combination with AURKA overexpression led to an increase of G2/M phase cells in both CHLA06 and BT16 cells (Fig. 6D).”*

*Figure 6:* ***C*** *Effect of gain of AURKA on the proliferation of ATRT cells on control and sgAMBRA1 background.*

3. The authors argued for synthetic lethality of AURKA and CDK4/6 inhibition upon loss of AMBRA1. It is important to determine how often AMBRA1 is lost in ATRT patients. If genome sequencing data from projects like TCGA shows frequent loss of AMBRA1 in patients, the authors' discovery would be more significant for treatment insights.

*Response:*

*We thank this reviewer for this important point. ATRT is a rare tumor and not well represented in public databases such as TCGA. Thus, we first check the studies from Johann et al. 2016 and Tochia et al. 2016 [7, 8], to our knowledge to biggest ATRT cohort with a total of n=383 cases with available sequencing data. However, we did not find any evidence for AMBRA1 alterations in ATRT.*

*We next interrogated the TCGA PanCancer Atlas Studies (n=10,967 tumor samples) for AMBRA1 alterations, which however does not include ATRT cases. We find that overall frequency of AMBRA1 alterations is low, with a maximum of roughly 10% in endometrial carcinoma and melanoma. However, to our surprise, even cancer types with well investigated tumor suppressive activity of AMBRA1 such as lung adenocarcinoma [9] only showed a low incidence of roughly 2% of AMBRA1 alterations, even though this tumor entity is overrepresented in the Atlas Studies (n=566). However, downregulation of tumor suppressive activity might also be accomplished by other mechanisms such as copy number variations or, as previously shown for AMBRA1 in diffuse large B-cell lymphoma, promoter hypermethylation [10]. We added these findings to the Results section on page 18:*

*“In line with that, genetic alterations of both AMBRA1 and BRAF are prevalent in melanoma (Additional file 1: Fig. 10B), while no AMBRA1 alterations have been identified in ATRT so far [9, 11].”*

Minor concerns:

1. Page 5, Line 108: "next generating sequencing" should be "next generation sequencing".

*Response:*

*This has been corrected accordingly in the revised version of the manuscript.*

2. Are the different ATRT cell lines varied in terms of cell-of-origin?

*Response:*

*The cell of origin for ATRT is under constant debate, and several potential cells of origin have been nominated including neural progenitors, neural crest, mid/hindbrain progenitors, as well as primordial germ cells [7, 11-14]. While there is some evidence for subgroup-specific origins (e.g. MYC rhabdoid tumors might derive from primordial germ cells [14]), even though we have good subgroup assignments for our ATRT cell lines, there is too much uncertainty about the cell of origin to draw conclusions.*

*Our new analyses regarding genetic interaction analyses from the DepMap for AMBRA1 (Fig. 5D-F; Fig. S10) strongly suggest a potential neural crest origin as one potential requirement for AMBRA1 acting as a tumor suppressor. While this would nicely explain tumor suppressor activity in melanoma and rhabdoid tumors, DepMap data does not recapitulate previous findings of KRAS-mediated suppressor activity in lung adenocarcinoma [9].*

3. In Fig. 2E, the CPM method may be outdated for transcriptome quantification; TPM is a better parameter.

*Response:*

*We agree with this reviewer and have adjusted our analyses. We believe that the CPM (counts per million) normalization method was inadequate, but TPM (transcripts per kilobase million) has limitations for comparisons across samples as well [15]. We now use DESeq2’s median of ratios normalization method [16]. Additionally, we performed the DESeq2 implemented regularized-logarithm transformation (rld) of counts. While this transformation does not differ much from ordinary log2 transformation for genes with high counts, the counts for low expressed genes are shrunken towards the genes’ average across all samples. Of note, these modifications did not change any of the previously made claims. We have added these new data in Fig. 3E and Fig. S4C.*

*Figure 3:* ***E*** *Correlation analyses to illustrate CDK4 or CDK6 dependency prediction by CCND2 and CDK6 mRNA expression. Dependency for the gene on the y axis is indicated by red color.*

*Figure S4:* ***C*** *Scatter plots illustrating the correlation of gene dependency scores and expression for selected genes known to be context-specific essentials across cancer cell lines according to DepMap (FANCI) or genes with distinct essentiality profiles in our study of ATRT cell lines (MYC, CDK6, FGFR2).*

4. For Fig. 3B, it would be better to mark drugs and cytotoxic agents in different colors.

*Response:*

*We agree with this reviewer, and have now colored cytotoxic drugs in new Fig. 2B (previous Fig. 3B) according to the previously established color coding from Fig. 2D for cytotoxic agents.*

5. For Fig. 4C, the y-axis label should be corrected from GRAOC to GRiAOC.

*Response:*

*This mistake has been corrected in what is now Fig. S6B.*

6. Fig. 5D shows that CHLA266 is not significantly dependent on D-type cyclin, making it inappropriate to state in Lines 310-312 that "all ATRT cell lines ...".

*Response:*

*We agree with this reviewer and apologize sorry for this inconvenience. While indeed all ATRT cell lines investigated in this study depend on either CDK4 or CDK6, in fact CHLA266 cells do not require any of the D-type cyclins at an FDR cutoff of less than 20%. We have thus clarified the corresponding sentence within the Results section on page 13 as follows:*

*“All ATRT cell lines depend either on CDK4 or CDK6 expression in a mutually exclusive manner, a pattern recapitulated by most human cancer cell lines [25], and most require expression of at least one out of three D-type cyclins (Fig. 3B; Additional file 1: Fig. S7F).”*

7. Page 13, Line 301: "response the" should be "response to the".

*Response:*

*This has been corrected accordingly in the revised version of the manuscript.*

8. Page 18, Line 409: "cells lines" should be "cell lines".

*Response:*

*This has been corrected accordingly in the revised version of the manuscript*.

9. Instead of merely citing DGIdb, the Methods section should include an analytical detail explaining whether the connected gene/drug pair was built upon specific tissue/cell type.

*Response:*

*Drug-gene interactions within DGIdb have been mined from several resources such as DrugBank, PharmGKB, ChEMBL, Drug Target Commons, and others [17]. As such, while certain drug-gene interactions might specifically derive from tissue-specific databases or papers focusing on specific tumor entities such as lung cancer [18], DGIdb consolidates and organizes drug-gene interactions in a tissue/cell type agnostic manner. Furthermore, DGIdb organizes the druggable genome in two classes: genes with known drug interactions, as well as genes that are in potentially druggable categories. In our study, we used all genetic dependencies identified in ATRT cell lines to identify genes that have a known drug interaction. This is now outlined in the Methods section on page 46 as follows:*

*“Based on all genetic dependencies identified in any of the ATRT cell lines, we used the Drug Gene Interaction database (DGIdb) to identify potential chemical vulnerabilities [27]. DGIdb consolidates and organizes the druggable genome in a tissue/cell type agnostic manner in two classes: genes with known drug interactions, as well as genes that are in potentially druggable categories. All drug gene interactions with a potential small molecule inhibitor or antagonist were considered.”*

Reviewer #3:

In the manuscript entitled "Functional screening reveals genetic dependencies and diverging cell cycle control in atypical teratoid rhabdoid tumors," Merck et al. used CRISPR-based genetic screens to find vulnerabilities in atypical teratoid rhabdoid tumors (ATRTs). The authors then validated certain hits with small molecule drug library and homed in on G1-cell cycle regulators, with a focus on CDK4/6 inhibitors. The authors further show that 'ubiquitin ligase substrate receptor AMBRA1 acts as a context-specific inhibitor of cell cycle progression.' Overall, this work is of value to the field and will be of interest to readers of Genome Biology after addressing the following comments.

*Response:*

*We thank this reviewer for this very encouraging and positive feedback.*

Major comments:

Based on their known importance, it would be likely that some of the 9 compounds tested for toxicity in vitro (ex. The inhibitors of CDK4/6, MAPK, and EGF) would have clinical or preclinical in vivo toxicity information available that should be noted/compared to.

*Response:*

*We agree that additional references with regard to clinical or preclinical toxicity profiles for most promising compounds from our drug library will be helpful for the reader, and have thus added a short comment to the Discussion section of the revised manuscript on page 24. Of note, we find that both CDK4/6 as well as EGFR inhibitors have been intensively studied in clinical trials [19-23], providing evidence for acceptable toxicity profiles for these drugs. MAPK inhibitors, the other class of drugs that is represented by more than one compound in our library, are less well studied in a clinical setting, but show promising results in preclinical model systems [24, 25]. We have added these references to the Discussion section as follows on page 24:*

*“Among others, these include several classes of inhibitors that did not show any neurotoxicity in our study and present manageable toxicity profiles in the clinic for CDK4/6 [52-54] and EGFR inhibitors [55, 56], while others such as MAPK inhibitors show promising results in preclinical research [57, 58].”*

To make the claim that mutated KRAS/BRAF tumors are likely sensitive to AMBRA1 loss, the authors should include an analysis of available large-scale data sets as support.

*Response:*

*We agree with this reviewer and performed a deeper characterization of the context-specific tumor suppressive role of AMBRA1. Based on this reviewer`s suggestion, we had a closer look at AMBRA1 gene knockout effects in the Broad’s dependency map. We find that AMBRA1 knockout does not show a significant effect in the majority of cell lines (Fig. S10A). However, cell lines from distinct primary diseases show a significant deviation from the rest of cell lines, and melanoma and rhabdoid cancer cell lines suggest a tumor suppressive role for AMBRA1 (Fig. 5D). Next, we assessed genetic interaction of AMBRA1 using the top 100 correlated genes. Here, we specifically focused on genes that show a skewed t-distribution in gene effects across cancer cell lines [26], thus have context-specific gene effects. While AMBRA1 shows direct correlation with several well-known tumor suppressors such as TP53, intriguingly AMBRA1 has inverse correlations with neural crest and melanocyte markers SOX10 and MITF, and the melanoma-enriched oncogene BRAF (Fig. 5E). Melanocytes derive from the neural crest, and this structure has been suggested as a potential origin for rhabdoid tumors as well [11-13], suggesting that neural crest origin might predict tumor suppressive activity of AMBRA1. Furthermore, AMBRA1 and BRAF genetic alterations are prevalent in several shared entities, most notably melanoma (Fig. S10B), and oncogenic signaling of BRAF in melanoma is correlated with AMBRA1 tumor suppressive activity (Fig. 5F). While melanoma cell lines mostly depend on SOX10 and MITF, both rhabdoid cell lines from DepMap as well as ATRT cell lines do* *not (Fig. S10C,D).*

*Figure 5:* ***D*** *Effect of AMBRA1 knockout in 1,150 human cancer cell lines from DepMap. Boxes illustrate primary diseases in which the knockout effect of AMBRA1 significantly differed from the all other cell lines. HNSCC: Head and Neck Squamous Cell Carcinoma.* ***E*** *The top 100 pre-computed genetic associations for AMBRA1 in DepMap. Selected genes that show a skewed gene effect distribution across human cancer cell lines are indicated.* ***F*** *Correlation of AMBRA1 and BRAF gene knockout effects in DepMap, highlighting melanoma and rhabdoid cancer cell lines.*

*With regard to KRAS signaling, DepMap data did not support any tumor suppressive role of AMBRA1 in entities known to be driven by oncogenic KRAS, including lung adenocarcinoma (Fig. S10E). This is in contrast to the previously described interaction of oncogenic KRAS and AMBRA1 in a mouse model of lung adenocarcinoma [9].We conclude that DepMap recapitulates at least in part context-specific tumor suppressive activity of AMBRA1, and this activity might in part be associated with tumor origin from the neural crest. We have added these new data to the Results section on page 18 as follows:*

*“In order to put these findings into a broader context, we interrogated the DepMap (https://depmap.org/) for knockout effects and potential genetic interactions of AMBRA1 across 1,150 human cancer cell lines. Indeed, while knockout of AMBRA1 did not affect the vast majority of cell lines, individual primary diseases showed a unique response pattern, and knockout effects in melanoma and rhabdoid cancer cell lines suggested a tumor suppressive role for AMBRA1 (Fig. 5D; Additional file 1: Fig. S10A). In line with these data, we found a strong direct correlation of AMBRA1 with several tumor suppressors including TP53 across DepMap, while inverse correlation was seen for selectively essential transcription factors defining neural crest (SOX10) and melanocyte (MITF) lineage (Fig. 5E). In melanoma, tumor suppressive activity of AMBRA1 was correlated with oncogenic BRAF signaling (Fig. 5F), corroborating previous results [43]. In line with that, genetic alterations of both AMBRA1 and BRAF are prevalent in melanoma (Additional file 1: Fig. 10B), while no AMBRA1 alterations have been identified in ATRT so far [9, 11]. Interestingly, even though rhabdoid tumors have been suggested to potentially derive from neural crest cells similar to melanocytes [44-46], neither rhabdoid cell lines from DepMap nor ATRT cell lines from our study were dependent on SOX10 or MITF (Additional file 1: Fig. S10C,D). Furthermore, while context-dependent tumor suppressive activity of AMBRA1 has been associated with oncogenic KRAS signaling in lung adenocarcinoma [42], we did not find any correlation of KRAS and AMBRA1 in tumors which show recurrent oncogenic KRAS mutations (Additional file 1: Fig. 10E). Together, these analyses reveal context-specific tumor suppressor activity of AMBRA1 which might be related to descent from the neural crest lineage.”*

*Figure S10: AMBRA1 gene effect associations from DepMap and mutation frequency.* ***A*** *AMBRA1 knockout effect across 1,150 human cancer cell lines from DepMap.* ***B*** *Frequency of AMBRA1 (left) and BRAF (right) alterations in 10,967 tumor tissue samples from the TCGA PanCancer Atlas Studies. The top 10 tumor entities are shown.* ***C*** *Correlation of SOX10 or MITF and AMBRA1 gene knockout effects in melanoma and rhabdoid cancer cell lines. Data for all other cell lines in DepMap are shown as comparison.* ***D*** *Scatter plot illustrating the effect of SOX10 and MITF knockout in ATRT cell lines.* ***E*** *Correlation of KRAS and AMBRA1 knockout effects in lung, pancreatic, and colorectal adenocarcinoma cell lines from DepMap.*

*Furthermore, we discuss these findings in detail within the Discussion section on pages 28 and 29 as follows:*

*“AMBRA1 has been described as a tumor suppressor in diffuse large B-cell lymphoma (DLBCL) [40], lung adenocarcinoma (LUAD) [41, 42], melanoma [43], and these entities may harbor mutations in AMBRA1. While it is surprising that ATRT do not seem to harbor AMBRA1 mutations [9, 11], both loss-of-function studies in ATRT cells as well as genetic interaction analyses from DepMap performed in this study strongly support a tumor suppressive role of AMBRA1 in neural crest derivatives including melanoma and rhabdoid tumors. In addition to a potential lineage association, oncogenic KRAS signaling is the best predictor for a tumor suppressive role of AMBRA1 in LUAD [42]. While this was not recapitulated by data from DepMap, we show here that dependency on oncogenic BRAF signaling correlates with AMBRA1 tumor suppressive activity in melanoma. In fact, all genetic mouse models describing tumor suppressive activity of AMBRA1 so far have been generated on a mutated KRAS/BRAF/MAPK signaling background, and mutations of the KRAS/BRAF/MAPK pathway are common in DLBCL, LUAD and melanoma [77, 78]. Both oncogenic BRAF and KRAS signaling has recently been shown to act through regulation of kinases involved in mitosis including aurora kinase A and B [79, 80], and mitotic regulators are enriched for synthetic lethalities in KRAS mutated tumors [81].”*

AlphaFold multimer structures should be used with some caution. As a 'negative control,' the authors should predict the co complex of AMBRA and a cyclin protein homolog that AMBRA is not expected to interact with. If there is a similar interaction as seen with the included AMBRA interaction partners, then those predicted interactions cannot be trusted.

*Response:*

*We agree with this reviewer that AlphaFold predictions should only be used as an indicator to support protein-protein interactions, and we provide more direct analyses (affinity purifications) to investigate potential interactions throughout the revised manuscript. Based on these valuable comments of this reviewer, we have now added additional AlphaFold multimer predictions that potentially serve as suitable “negative controls” for AMBRA1-specific interactions. We have here focused on cyclin A2 and cyclin B1, as these two cyclins were unaffected by loss of AMBRA1 in all ATRT cell lines investigated (Fig. 6A). Using intrinsic model accuracy estimates (pTM scores), we find that complex predictions for these two cyclins (pTM=0.54) are lower than for any other predicted AMBRA1 substrate (pTM range 0.68-0.59) investigated in our study. In particular, per-residue confidence metrics (pLDDT) suggest lower complex accuracy at the potential interaction surfaces of cyclin A2 and cyclin B1 with the AMBRA1-WD40 domain as compared to AURKB. Together, these data are in line with our observations of changes on the protein level upon loss of AMBRA1, and provide further evidence for substrate selectivity of AMBRA1.*

*These new analyses are now shown in Fig. S12B.*

*Figure S12:* ***B*** *Protein complex structure prediction using AlphaFold2_mmseqs2 for AMBRA1^WD40^ with AURKB as compared to negative controls CCNA2 and CCNB1. Top, cartoon illustration of predicted heteromeric protein complexes. Per-residue confidence pLDDT metric is color-coded. pTM scores for all complexes are indicated. Bottom, pairwise PAE scores for all protein complexes shown.*

*We have incorporated these new findings within the Results section on page 20 as follows:*

*“Of note, model accuracy estimates were higher for substrates shown to be affected by loss of AMBRA1 (pTM range 0.68-0.59) than estimates for cyclin genes unaffected by ablation of AMBRA1 (pTM=0.54 for cyclin A2 and cyclin B1) (Additional file 1: Fig. S12B).”*

Minor comments:

(1) It's unclear of the subtype of the seven selected ATRT cell lines when they are introduced in the text. Please make note of this or move S1H to Fig1. As the authors also stress clinical relevance throughout, ATRT subtypes should be specified when discussing therapeutic response (e.g., p14 for CDK4/6 dependent cell lines).

*Response:*

*We apologize for our lack of clarity. Now, we clearly indicate subgroup assignments for the seven ATRT cell lines as inferred from our integrated analyses using both gene expression as well as DNA methylation profiling on page 7 within the Results section as follows:*

*“Of note, our integrated analyses led to distinct subgroup assignments for these seven cell lines to ATRT-SHH (CHLA02, CHLA04, CHLA05) or ATRT-TYR/MYC subgroups (CHLA06, CHLA266, BT12, BT16) (Additional file 1: Fig. S1C-H), being in line with previous subgroup assignments for these lines [12]”*

(2) The word 'signaling' is often capitalized, which appears to be a typo.

*Response:*

*We are sorry for this mistake in the previous manuscript, which we corrected in the revised version.*

(3) It is unclear what figures 2B and 2C are supposed to inform the reader of.

*Response:*

*We apologize for the lack of clarity. We now explained our approach to identify potential associations of gene dependency with gene promoter methylation or gene expression levels in the revised manuscript in more detail on pages 8 and 9. Sparse projection to latent structures (sPLS) is a robust multivariate projection-based method that can be used to explain or explore the relationship between continuous datasets [27]. In this study, we used sPLS as an exploratory approach to reveal potential associations between gene promoter methylation, gene expression, and gene dependency. It is well appreciated that promoter methylation impacts on the level of gene expression, and we recapitulate this by extracting relevant correlations when comparing these two datasets (Fig. 1F, left panel). Of note, we find the same degree of association for gene expression and gene dependency (Fig. 1F, middle panel), suggesting that gene expression is a primary predictor for gene dependency. Last, as highlighted in the initial text, we find clear a clear indication that low promoter methylation (i.e. leading to high gene expression) strongly correlates with gene dependency (Fig. 1F, right panel). Last, these observations were recapitulated using multiblock sPLS, methodically similar to sPLS, but able to correlated more than two continuous datasets at once. While we did not use sPLS for feature prediction due to small number of ATRT cell lines available for screening, these exploratory approaches strongly highlight to potential of predictive markers to inform on genetic dependencies in ATRT. We have modified the text in the Results section on pages 8 and 9 in order to highlight our rationale, and reference appropriate literature on the sPLS method as follows:*

*“We therefore performed sparse projection to latent structures (sPLS) in an unsupervised fashion to explore the relationship of DNA promoter methylation, gene expression, and gene dependency scores in ATRT cells [26]. Top selected features showed strong clustering among the first two components in pairwise comparisons and revealed relevant associations both in pairwise as well as multiblock comparisons (Fig. 1F; Additional file 1: Fig. S4B). Of note, while we found both positive as well as negative correlations of gene expression features with gene dependency scores, our data revealed exclusive positive correlations of promoter methylation and dependency scores, where low promoter methylation levels, arguably leading to higher gene expression, are associated with gene dependency.”*

*References*

*1.	McDonald ER, 3rd, de Weck A, Schlabach MR, Billy E, Mavrakis KJ, Hoffman GR, Belur D, Castelletti D, Frias E, Gampa K et al:* ***Project DRIVE: A Compendium of Cancer Dependencies and Synthetic Lethal Relationships Uncovered by Large-Scale, Deep RNAi Screening****. Cell 2017,* ***170****(3):577-592 e510.*

*2.	Ho B, Johann PD, Grabovska Y, De Dieu Andrianteranagna MJ, Yao F, Fruhwald M, Hasselblatt M, Bourdeaut F, Williamson D, Huang A et al:* ***Molecular subgrouping of atypical teratoid/rhabdoid tumors-a reinvestigation and current consensus****. Neuro Oncol 2020,* ***22****(5):613-624.*

*3.	Oberlick EM, Rees MG, Seashore-Ludlow B, Vazquez F, Nelson GM, Dharia NV, Weir BA, Tsherniak A, Ghandi M, Krill-Burger JM et al:* ***Small-Molecule and CRISPR Screening Converge to Reveal Receptor Tyrosine Kinase Dependencies in Pediatric Rhabdoid Tumors****. Cell Rep 2019,* ***28****(9):2331-2344 e2338.*

*4.	Tsikitis M, Zhang Z, Edelman W, Zagzag D, Kalpana GV:* ***Genetic ablation of Cyclin D1 abrogates genesis of rhabdoid tumors resulting from Ini1 loss****. Proc Natl Acad Sci U S A 2005,* ***102****(34):12129-12134.*

*5.	Fujisawa H, Misaki K, Takabatake Y, Hasegawa M, Yamashita J:* ***Cyclin D1 is overexpressed in atypical teratoid/rhabdoid tumor with hSNF5/INI1 gene inactivation****. J Neurooncol 2005,* ***73****(2):117-124.*

*6.	Versteege I, Medjkane S, Rouillard D, Delattre O:* ***A key role of the hSNF5/INI1 tumour suppressor in the control of the G1-S transition of the cell cycle****. Oncogene 2002,* ***21****(42):6403-6412.*

*7.	Torchia J, Golbourn B, Feng S, Ho KC, Sin-Chan P, Vasiljevic A, Norman JD, Guilhamon P, Garzia L, Agamez NR et al:* ***Integrated (epi)-Genomic Analyses Identify Subgroup-Specific Therapeutic Targets in CNS Rhabdoid Tumors****. Cancer Cell 2016,* ***30****(6):891-908.*

*8.	Johann PD, Erkek S, Zapatka M, Kerl K, Buchhalter I, Hovestadt V, Jones DTW, Sturm D, Hermann C, Segura Wang M et al:* ***Atypical Teratoid/Rhabdoid Tumors Are Comprised of Three Epigenetic Subgroups with Distinct Enhancer Landscapes****. Cancer Cell 2016,* ***29****(3):379-393.*

*9.	Chaikovsky AC, Li C, Jeng EE, Loebell S, Lee MC, Murray CW, Cheng R, Demeter J, Swaney DL, Chen SH et al:* ***The AMBRA1 E3 ligase adaptor regulates the stability of cyclin D****. Nature 2021,* ***592****(7856):794-798.*

*10.	Simoneschi D, Rona G, Zhou N, Jeong YT, Jiang S, Milletti G, Arbini AA, O'Sullivan A, Wang AA, Nithikasem S et al:* ***CRL4(AMBRA1) is a master regulator of D-type cyclins****. Nature 2021,* ***592****(7856):789-793.*

*11.	Han ZY, Richer W, Freneaux P, Chauvin C, Lucchesi C, Guillemot D, Grison C, Lequin D, Pierron G, Masliah-Planchon J et al:* ***The occurrence of intracranial rhabdoid tumours in mice depends on temporal control of Smarcb1 inactivation****. Nat Commun 2016,* ***7****:10421.*

*12.	Vitte J, Gao F, Coppola G, Judkins AR, Giovannini M:* ***Timing of Smarcb1 and Nf2 inactivation determines schwannoma versus rhabdoid tumor development****. Nat Commun 2017,* ***8****(1):300.*

*13.	Custers L, Khabirova E, Coorens THH, Oliver TRW, Calandrini C, Young MD, Vieira Braga FA, Ellis P, Mamanova L, Segers H et al:* ***Somatic mutations and single-cell transcriptomes reveal the root of malignant rhabdoid tumours****. Nat Commun 2021,* ***12****(1):1407.*

*14.	Graf M, Interlandi M, Moreno N, Holdhof D, Gobel C, Melcher V, Mertins J, Albert TK, Kastrati D, Alfert A et al:* ***Single-cell transcriptomics identifies potential cells of origin of MYC rhabdoid tumors****. Nat Commun 2022,* ***13****(1):1544.*

*15.	Zhao S, Ye Z, Stanton R:* ***Misuse of RPKM or TPM normalization when comparing across samples and sequencing protocols****. RNA 2020,* ***26****(8):903-909.*

*16.	Love MI, Huber W, Anders S:* ***Moderated estimation of fold change and dispersion for RNA-seq data with DESeq2****. Genome Biol 2014,* ***15****(12):550.*

*17.	Cotto KC, Wagner AH, Feng YY, Kiwala S, Coffman AC, Spies G, Wollam A, Spies NC, Griffith OL, Griffith M:* ***DGIdb 3.0: a redesign and expansion of the drug-gene interaction database****. Nucleic Acids Res 2018,* ***46****(D1):D1068-D1073.*

*18.	Simon GR, Somaiah N:* ***A tabulated summary of targeted and biologic therapies for non-small-cell lung cancer****. Clin Lung Cancer 2014,* ***15****(1):21-51.*

*19.	Turner NC, Huang Bartlett C, Cristofanilli M:* ***Palbociclib in Hormone-Receptor-Positive Advanced Breast Cancer****. N Engl J Med 2015,* ***373****(17):1672-1673.*

*20.	Dickler MN, Tolaney SM, Rugo HS, Cortes J, Dieras V, Patt D, Wildiers H, Hudis CA, O'Shaughnessy J, Zamora E et al:* ***MONARCH 1, A Phase II Study of Abemaciclib, a CDK4 and CDK6 Inhibitor, as a Single Agent, in Patients with Refractory HR(+)/HER2(-) Metastatic Breast Cancer****. Clin Cancer Res 2017,* ***23****(17):5218-5224.*

*21.	Rugo HS, Huober J, Garcia-Saenz JA, Masuda N, Sohn JH, Andre VAM, Barriga S, Cox J, Goetz M:* ***Management of Abemaciclib-Associated Adverse Events in Patients with Hormone Receptor-Positive, Human Epidermal Growth Factor Receptor 2-Negative Advanced Breast Cancer: Safety Analysis of MONARCH 2 and MONARCH 3****. Oncologist 2021,* ***26****(3):e522.*

*22.	Miller VA, Hirsh V, Cadranel J, Chen YM, Park K, Kim SW, Zhou C, Su WC, Wang M, Sun Y et al:* ***Afatinib versus placebo for patients with advanced, metastatic non-small-cell lung cancer after failure of erlotinib, gefitinib, or both, and one or two lines of chemotherapy (LUX-Lung 1): a phase 2b/3 randomised trial****. Lancet Oncol 2012,* ***13****(5):528-538.*

*23.	Reddick SJ, Campagne O, Huang J, Onar-Thomas A, Broniscer A, Gajjar A, Stewart CF:* ***Pharmacokinetics and safety of erlotinib and its metabolite OSI-420 in infants and children with primary brain tumors****. Cancer Chemother Pharmacol 2019,* ***84****(4):829-838.*

*24.	Nichols RJ, Haderk F, Stahlhut C, Schulze CJ, Hemmati G, Wildes D, Tzitzilonis C, Mordec K, Marquez A, Romero J et al:* ***RAS nucleotide cycling underlies the SHP2 phosphatase dependence of mutant BRAF-, NF1- and RAS-driven cancers****. Nat Cell Biol 2018,* ***20****(9):1064-1073.*

*25.	Frank KJ, Mulero-Sanchez A, Berninger A, Ruiz-Canas L, Bosma A, Gorgulu K, Wu N, Diakopoulos KN, Kaya-Aksoy E, Ruess DA et al:* ***Extensive preclinical validation of combined RMC-4550 and LY3214996 supports clinical investigation for KRAS mutant pancreatic cancer****. Cell Rep Med 2022,* ***3****(11):100815.*

*26.	Pacini C, Dempster JM, Boyle I, Goncalves E, Najgebauer H, Karakoc E, van der Meer D, Barthorpe A, Lightfoot H, Jaaks P et al:* ***Integrated cross-study datasets of genetic dependencies in cancer****. Nat Commun 2021,* ***12****(1):1661.*

*27.	Le Cao KA, Martin PG, Robert-Granie C, Besse P:* ***Sparse canonical methods for biological data integration: application to a cross-platform study****. BMC Bioinformatics 2009,* ***10****:34.*

**Second round of review**

**Reviewer 2**

I appreciate the authors' thorough responses to each of my point. With the explanations and additional experimental data, the revised manuscript is much more readable and comprehensive. The authors have conducted systematic work on atypical teratoid rhabdoid tumors, which will be undoubtedly valuable for the field. I only have two concerns now.

One major concern is related to the synthetic lethality between AURKA and CDK4/6 inhibition upon the loss of AMBRA1. The synthetic lethality is only significantly demonstrated in AMBRA1 knockout conditions of AMBRA1-responder cell lines (Fig. 8C). Given the potentially low natural occurrence of AMBRA1 loss in ATRT tumors, the broader applicability of these methods may be limited. As the authors mentioned in their response, the downregulation and methylation of AMBRA1, rather than genetic alterations, might drive tumor development. Verification of this hypothesis through RNAi or computational inference would be compelling. I certainly recognize the substantial effort already invested in this manuscript. So if authors could not perform additional experiments or analyses to strengthen this final section, please tone down the writing.

As one minor suggestion, it would be helpful to annotate the positive controls (dasatinib, nilotinib, dorsomorphin, DAPT, ML329) in Fig 2B using different colors or labels to make the conclusions easier to follow.

**Reviewer 3**

The authors have addressed all prior comments and the manuscript should be ready for publication in Genome Biology.

**Authors Response**

**Point-by-point responses to the reviewers’ comments:**

Reviewer #2:

I appreciate the authors' thorough responses to each of my point. With the explanations and additional experimental data, the revised manuscript is much more readable and comprehensive. The authors have conducted systematic work on atypical teratoid rhabdoid tumors, which will be undoubtedly valuable for the field.

*Comment: We thank this reviewer for this overall very positive comment.*

I only have two concerns now.

One major concern is related to the synthetic lethality between AURKA and CDK4/6 inhibition upon the loss of AMBRA1. The synthetic lethality is only significantly demonstrated in AMBRA1 knockout conditions of AMBRA1-responder cell lines (Fig. 8C). Given the potentially low natural occurrence of AMBRA1 loss in ATRT tumors, the broader applicability of these methods may be limited. As the authors mentioned in their response, the downregulation and methylation of AMBRA1, rather than genetic alterations, might drive tumor development. Verification of this hypothesis through RNAi or computational inference would be compelling. I certainly recognize the substantial effort already invested in this manuscript. So if authors could not perform additional experiments or analyses to strengthen this final section, please tone down the writing.

*Comment: We thank this reviewer for this important point. We have revised the writing in both the Results and Discussion section. We now refrain from referring to a cellular model within the Results section, which now reads as follows on page 23:*

*“Simultaneous blockade of cell cycle progression in G1 and G2/M phase using CDK4/6 and AURKA inhibitors showed synergistic interaction only in AMBRA1 responder cells with an AMBRA1 knockout (Fig. 8C), further suggesting that tumor cells in which AMBRA1 fulfills a tumor suppressive role might be particularly sensitive to AURKA inhibition.”*

*Furthermore, we now discuss this synthetic lethality in more detail within the Discussion section, emphasizing the limitations of our analyses, and discussing future directions. This part reads as follows on pages 29 and* *30:*

*“Both oncogenic BRAF and KRAS signaling has recently been shown to act through regulation of kinases* *involved in mitosis including aurora kinase A and B [79, 80], and based on our findings of an AMBRA1-dependent regulation of mitotic kinases, it seems plausible that tumors with high activity of mitotic regulators might be particularly vulnerable to loss of AMBRA1. However, further genetic and biochemical studies will be necessary to decipher the exact molecular mechanism of cell type-specific selectivity of AMBRA1-dependent regulation of mitotic regulators.*

*Conversely, KRAS mutated tumors have been shown to present synthetic lethalities particularly for mitotic regulators [81], and we here find that ATRT cells with an oncogenic loss AMBRA1 are particularly sensitive to AURKA inhibition. Also, while only tested in a small number of ATRT models, oncogenic loss of AMBRA1 might also predispose to synthetic lethality of CDK4/6 and AURKA inhibition, likely a result of the concerted regulation of these important G1/S and G2/M phase mediators by AMBRA1. However, ATRT cell do not typically present alterations of AMBRA1, and other potential mechanisms of AMBRA1 downregulation need to be determined. Furthermore, the generalisability of this synthetic lethality needs to be further investigated in tumors that frequently present AMBRA1 alterations.”*

As one minor suggestion, it would be helpful to annotate the positive controls (dasatinib, nilotinib, dorsomorphin, DAPT, ML329) in Fig 2B using different colors or labels to make the conclusions easier to follow.

*Comment: We have revised this accordingly, now indicating small molecule drugs serving as positive controls in blue in Figure 2B*.

Reviewer #3:

The authors have addressed all prior comments and the manuscript should be ready for publication in Genome Biology.

*Comment: We thank this reviewer for this positive comment, much appreciated.*
